# Cyclopeptide Derivatives from the Sponge-Derived Fungus *Acremonium persicinum* F10

**DOI:** 10.3390/md19100537

**Published:** 2021-09-24

**Authors:** Yingxin Li, Zhiyong Li

**Affiliations:** Marine Biotechnology Laboratory, State Key Laboratory of Microbial Metabolism, School of Life Sciences and Biotechnology, Shanghai Jiao Tong University, Shanghai 200240, China; liyingxinhappy@163.com

**Keywords:** *Acremonium persicinum*, cyclopeptides, siderophore, acremonpeptides, marine sponge-derived fungus, anti-fungal activity

## Abstract

Cyclopeptides usually play a pivotal role, either in the viability or virulence of fungi. Two types of cyclopeptides, six new hydroxamate siderophore cyclohexapeptides (**1**–**6**), including acremonpeptides E and F, and their complexes with aluminum and ferric ions; one new cyclic pentapeptolide, aselacin D (**9**); together with a known compound, aselacin C (**10**), were isolated and characterized from the sponge-derived fungus *Acremonium persicinum* F10. In addition, two new siderophore analogues chelating gallium ions (Ga^3+^), Ga (III)-acremonpeptide E (**7**) and Ga (III)-acremonpeptide F (**8**), using isolated acremonpeptides E and F, were prepared. The planar structures of **1**–**10** were elucidated by HRESIMS and (1D and 2D) NMR. The absolute configurations of amino acids were determined by means of the advanced Marfey’s method and X-ray single-crystal diffraction analysis. X-ray fluorescence (XRF) spectrometer was performed to disclose the elements of compound **1**, indicating the existence of aluminum (Al). Al (III)-acremonpeptides E (**1**), Ga (III)-acremonpeptides E (**5**), Al (III)-acremonpeptide F (**7**), and Ga (III)-acremonpeptide F (**8**) displayed high in vitro anti-fungal activities, which are comparable to amphotericin B, against *Aspergillus fumigatus* and *Aspergillus niger.*

## 1. Introduction

Siderophores from bacteria, fungi, and plants [1,2], assembled by non-ribosomal peptide synthetases (NRPSs), are high-affinity iron chelators responsible for iron acquisition and storage. Siderophores usually provide a “Trojan horse” strategy for humans to exploit new antibiotics using a combination a transporter and antibacterial or bactericidal moieties, such as cefiderocol, the recent Food and Drug Administration (FDA)-approved siderophore-conjugated antibiotic [3]. Hydroxamate-based peptidyl siderophores are the major type in fungi, including the extracellular siderophore, the depsipeptides and coprogen family, and the intracellular siderophore, the ferrichrome family, which enable the uptake of iron from surrounding environment [4]. Among them, ASP2397, a previously reported hydroxamate-based peptidyl siderophore, is a novel natural compound from *Acremonium persicinum* MF-347833, exhibiting potent fungicidal activity against the invasive *Aspergillus* genus with unique bacteriostatic mechanism [5,6]. Eight naturally occurring derivatives of ASP2397 have been isolated from *Acremonium persicinum* (Figure S1), some of which exhibit antifungal and antiviral activities, indicating their potentials as drug leads [5,7].

Marine-derived fungi are valuable resources for the exploration of structurally novel and bioactive compounds and drug leads [8,9,10,11]. In particular, chemical studies of marine sponge-derived fungi have afforded a variety of bioactive secondary metabolites [12,13,14]. Here, we report seven new cyclopeptide compounds from *Acremonium persicinum* F10 derived from marine sponge *Phakellia fusca* in the South China Sea.

**Figure 1:**
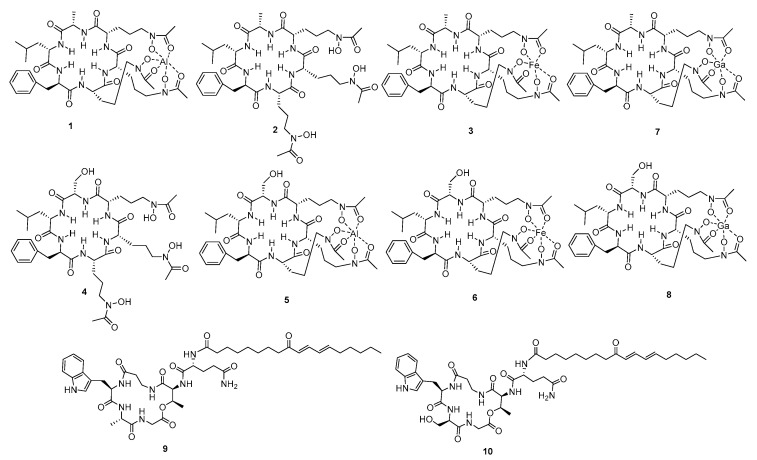
Chemical structures of compounds **1**–**10.**

Originally, sponge-derived fungal strains were screened by adopting the OSMAC (one strain many compounds) approach; as a result, we found that *Acremonium persicinum* F10 displayed the richest metabolites in the HPLC chemical profiles when statically cultured on rice medium. Consequently, three new cyclopeptides (**2**, **4**, **9**), four Al^3+^ and Fe^3+^ complexes of **2** and **4,** and a known compound **10** were isolated by scale-up fermentation of *A. persicinum* F10. In addition, two new chelates (compounds **7** and **8**) with Ga^3+^ were synthesized in vitro by two new ligands (compounds **2** and **4**) (Figure 1). Compounds **1**, **5**, **7**, and **8** exhibited excellent anti-fungal activities, comparable with positive control amphotericin B (Minimum Inhibitory Concentration, MIC at 1 μM). Meanwhile, all these compounds showed no cytotoxicity to normal cell (human embryonic lung fibroblast, MRC-5) at the concentration of 30 μM.

## 2. Results and Discussion

### 2.1. Structure Elucidation of Compounds ***1**–**10***

Al (III)-acremonpeptide E (**1**) was isolated as a white needle-like crystal. The molecular formulation was unable to be deduced by element composition of C, H, O, N, or S from the HRESIMS data *m*/*z* 872.4123 [M + H]^+^. X-ray fluorescence (XRF) spectrometry was performed to check the elements of compound **1**, and the existence of aluminum (Al) in **1** was confirmed by comparing with the control (Figure S2).

**Figure 2:**
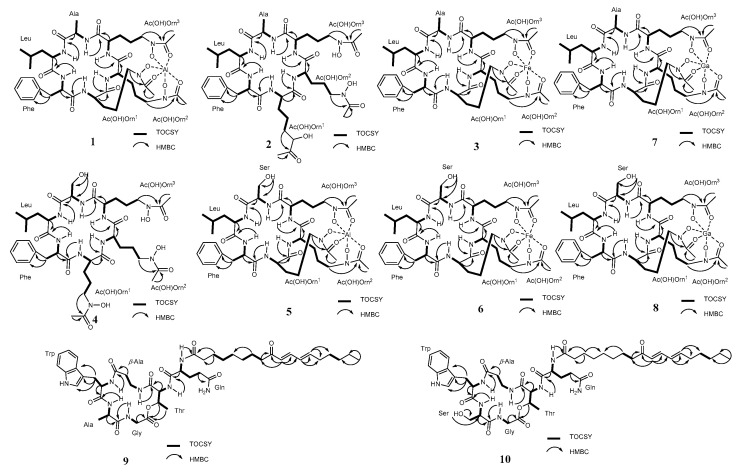
Key COSY and HMBC correlations of compounds **1**–**10.**

The molecular formula of **1** was finally deduced as C_39_H_58_N_9_O_12_Al (Calcd. for C_39_H_59_N_9_O_12_Al, *m*/*z* 872.4099 [M + H]^+^) in combination with the NMR data. The ^1^H and ^13^C spectra (Table 1) displayed resonances for six NH doublets (*δ*_H_ 10.10, 8.95, 8.44, 8.27, 7.49, and 6.28), six *α*-H (*δ*_H_ 3.87‒4.79), nine carbonyls (*δ*_C_ 174.6, 172.3, 171.1, 169.6, 169.4, 169.1, 161.7, 161.5, and 161.4), together with six *α*-methine carbon signals at 57.9, 55.7, 53.8, 52.5, 52.2, and 46.7, indicating at least six amino acid residues in compound **1**. A detailed analysis of the total correlation spectroscopy (TOCSY), heteronuclear single quantum coherence (HSQC), and heteronuclear multiple-bond correlation (HMBC) data allowed for the construction of an alanine (Ala), a leucine (Leu), a phenylalanine (Phe), and three ornithine (Orn) residues (Figure 2). HMBC correlations of three methyl signals with the remaining three carbonyls (*δ*_H_ 2.05/*δ*_C_ 161.5, *δ*_H_ 2.09/*δ*_C_ 161.7, and *δ*_H_ 2.09/*δ*_C_ 161.3), together with HMBC correlations of the methylenes in the Orn to these three carbonyls (*δ*_H_ 3.69/*δ*_C_ 161.5, *δ*_H_ 4.02/*δ*_C_ 161.7, and *δ*_H_ 3.41/*δ*_C_ 161.3) indicated that each Orn was modified by one acetyl group via an amide bond. The remaining three oxygens and aluminum were deduced to be located in 5-N atom of Orn to form *N*^5^-acetyl-*N*^5^-hydroxyornithine, then chelating with aluminum by detailed review of the literature concerning related structures [15]. Given that 12 of the 13 degrees of unsaturation were attributed to nine carbonyl carbons and a benzene ring, the absence degree of unsaturation suggest that **1** might be a cyclopeptide. The amino acid sequence in **1** of AcN(OH)Orn^1^-AcN(OH)Orn^2^- AcN(OH)Orn^3^-Ala-Leu-Phe was established by the HMBC correlations of Phe-NH (*δ*_H_ 8.95)/Leu-CO (*δ*_C_ 172.3), Leu-NH (*δ*_H_ 8.27)/Ala-CO (*δ*_C_ 171.1), Ala-NH (*δ*_H_ 7.49)/AcN(OH)Orn^3^-CO (*δ*_C_ 169.4), AcN(OH)Orn^3^-NH (*δ*_H_ 6.28)/AcN(OH)Orn^2^-CO (*δ*_C_ 174.6), and AcN(OH)Orn^2^-NH (*δ*_H_10.10)/AcN(OH)Orn^1^-CO (*δ*_C_ 169.1), which was further verified by ESI-MS/MS fragments at *m*/*z* 700.3237 [M‒AcN(OH)Orn + H]^+^, *m*/*z* 672.3278 [M‒AcN(OH)Orn-Ala + H]^+^, *m*/*z* 516.2032 [M‒AcN(OH)Orn-Ala-Leu + H]^+^, and *m*/*z* 369.1337 [M‒AcN(OH)Orn-Ala-Leu-Phe + H]^+^ (Figure 2 and Figure S10). The planar structure of **1** was further confirmed by X-ray diffraction analysis with Cu K*α* radiation accompanied by the Flack parameter of -0.10 (5), which also determined the absolute configuration assignments of _L_-Ala, _L_-Leu, _D_-Phe, _L_-Orn^1^, _L_-Orn^2^, and _L_-Orn^3^ in **1**. Thus, compound **1** was identified and named Al (III)-acremonpeptide E.

**Figure 3:**
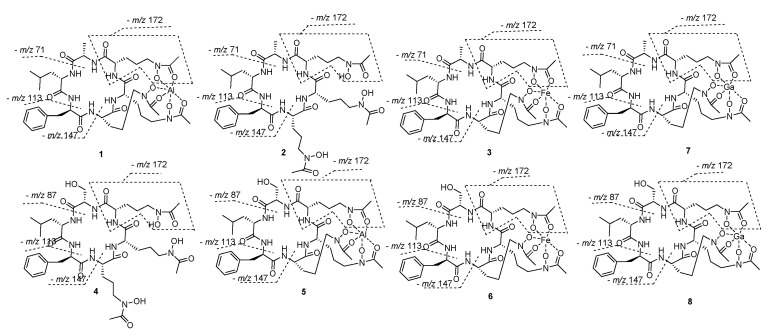
Fragmentation structure of compounds **1**–**8** by HRESIMS/MS.

Acremonpeptide E (**2**) was obtained as a faint yellow solid with a molecular formula of C_39_H_61_N_9_O_12_, on the basis of its high resolution electrospray ionization mass spectroscopy (HRESIMS) data, which suggested 14 degrees of unsaturation. Comparison of the ^1^H NMR and ^13^C NMR data of compound **2** (Table 1) with those of **1** suggested that both compounds shared similar structural features, except obvious shifts of the three acetyl group carbonyls (from *δ*_C_ 161.5, 161.7, and 161.3 in **1** to 170.8 in **2**) and methyls (from *δ*_C_ 15.9, 16.2, and 15.3 in **1** to 20.8 in **2**) in AcN(OH)Orn residues, forming overlapped carbons. These similar shifts were also observed in compound Al(III)-acremonpeptide D and acremonpeptide D or compound ASP2397 and AS2488059, indicating compound **2** was the ligand compound of **1** by deleting Al (III). As expected, detailed analysis of its TOCSY correlations allowed for the construction of an alanine (Ala), a leucine (Leu), a phenylalanine (Phe), and three *N*^5^-acetyl-*N*^5^-hydroxyornithines in **2** (Figure 2). Further, the amino acid sequence in **2** of AcN(OH)Orn^1^-AcN(OH)Orn^2^- AcN(OH)Orn^3^- Ala-Leu-Phe was established by the HMBC correlations as described in compound **1**. This assignment was also verified by the HRMS/MS fragment ion series at *m*/*z* 676.3670 [M‒AcN(OH)Orn + H]^+^, *m*/*z* 605.3288 [M‒AcN(OH)Orn-Ala + H]^+^, *m*/*z* 492.2444 [M‒AcN(OH)Orn-Ala-Leu + H]^+^, and *m*/*z* 345.1782 [M‒AcN(OH)Orn-Ala-Leu-Phe + H]^+^ (Figure 3 and Figure S21). The absolute configuration of the amino acid residues in **2** was determined using advanced Marfey’s method. The hydrolysates of **2** were derivatized with 1-fluoro-2,4-dinitrophenyl-5-_L_-leucinamide (_L_-FDLA) and analyzed by ultra performance liquid chromatography mass spectrometric (UPLC–MS). By comparing _L_-FDLA derivatives of amino acid from compound **2** with standard amino acid, we determined the _L_-Ala, _L_-Leu, and _D_-Phe residues in **2** (Figure S22), identical to compound **1** (Figure S11). The absolute configuration of AcN(OH)Orn units of compound **2** was deduced as the same as that of compound **1** with _L_-Orn residues, owing to a shared biosynthetic pathway. On the basis of these data, we established the structure of compound **2**, which was the ligand compound of **1,** acremonpeptide E.

**Figure 4:**
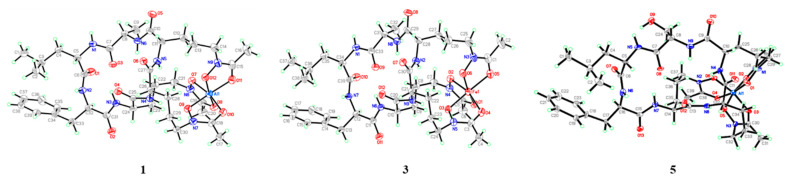
X-ray Oak Ridge thermal ellipsoid plot (ORTEP) drawings of compounds **1**, **3**, and **5.**

Fe (III)-acremonpeptide E (**3**), obtained as orange-red acicular crystal, had the HRESIMS data *m*/*z* 901.3745 [M + H]^+^ and *m*/*z* 923.3516 [M + Na]^+^. The existence of iron in compound **3** was deduced by disturbed ^1^H and ^13^C NMR records (Figures S25 and S26) in combination with conjectural molecular formula C_39_H_58_N_9_O_12_Fe (Calcd. for C_39_H_59_N_9_O_12_Fe, *m*/*z* 901.3633 [M + H]^+^). The same losing fragment AcN(OH)Orn (–*m*/*z* 172), Ala (–*m*/*z* 71), Leu (–*m*/*z* 113), and Phe (–*m*/*z* 113) in HRMS/MS data of compounds **3** and **1** indicated that they may possess the same ligand (Figure 3 and Figure S28). A high-quality crystal of **3** was subjected to single-crystal X-ray diffraction analysis with Cu K*α* (Figure 4) (Flack parameter = 0.04 (8)), suggesting the planar structure and absolute configuration of compound **3** were identical to **1**, except the substitution of metal ion Al (III) in **1** by Fe (III) in **3**. Accordingly, the structure of compound **3**, which was named Fe (III)-acremonpeptide E, was corroborated.

The molecular formula of acremonpeptide F (**4**), a faint yellow solid, was determined as C_39_H_61_N_9_O_13_ by the molecular ion at *m*/*z* 864.4471 [M + H]^+^ (Calcd. for 864.4467) in the HRESIMS data. The ^1^H NMR and ^13^C NMR data of **4** (Table 2) was similar to that of compound **2** (Table 2). Comparing the 1D NMR data of compound **4** with those of **2**, we found that the presence of an oxygen methylene group (*δ*_H_ 3.55 and 3.58, *δ*_C_ 61.0) and the absence of a methyl group (*δ*_H_ 1.24, *δ*_C_ 18.1) in **4** was the major difference. Detailed analysis of TOCSY and HMBC correlations demonstrated that the only discrepancy in compound **4** was a Ser residue for the Ala moiety substitution relative to **2**, which was supported by the HRESIMS/MS fragments at *m*/*z* 692.3599 [M‒AcN(OH)Orn + H]^+^ and *m*/*z* 605.3245 [M‒AcN(OH)Orn-Ser + H]^+^ (Figure 2, Figure 3, and Figure S38). The amino acid sequence of **4** was confirmed as AcN(OH)Orn^1^-AcN(OH)Orn^2^-AcN(OH)Orn^3^-Ala-Leu-Phe on the basis of HMBC corrections of Phe-NH (*δ*_H_ 8.87)/Leu-CO (*δ*_C_ 172.2), Leu-NH (*δ*_H_ 7.07)/Ser-CO (*δ*_C_ 169.6), Ser-NH (*δ*_H_ 7.58)/AcN(OH)Orn^3^-CO (*δ*_C_ 171.7), AcN(OH)Orn^3^-NH (*δ*_H_ 8.31)/AcN(OH)Orn^2^-CO (*δ*_C_ 171.7), and AcN(OH)Orn^2^-NH (*δ*_H_ 7.78)/AcN(OH)Orn^1^-CO (*δ*_C_ 171.8) (Figure 2). The absolute configuration of the amino acid residues in **4** was determined to be _L_-Ser, _L_-Leu, _D_-Phe, and three of _L_-AcN(OH)Orn^3^ according to Marfey’s method and a shared biogenesis. Thus, the structure of **4** was established and named acremonpeptide F.

Al (III)-acremonpeptide F (**5**), obtained as a white solid with an [M+H]^+^ ion at *m*/*z* 888.4073, had a molecular formular of C_39_H_58_N_9_O_13_Al (Calcd. for C_39_H_59_N_9_O_13_Al, *m*/*z* 888.4048 [M + H]^+^), as designated by the HRESIMS and ^13^C NMR data. The 1D NMR and 2D NMR spectra indicated that compound **5** possessed a similar structure to compound **4**, except that the overlapped carbon signals in **4** were clearly separated in **5** from the ^13^C NMR, which was also observed between compounds **1** and **2**. Therefore, we speculated that **5** was the siderophore-metal (III) complex of **4**. The crystal of compound **5** was obtained by repeated recrystallization from n-hexane-chloroform (1:1) and subjected to single-crystal X-ray diffraction analysis with Cu Kα (Figure 4) (Flack parameter = 0.02 (6)), further confirming its planar structure and absolute configuration as *cyclo*-(_L_-AcN(OH)Orn^1^-_L_-AcN(OH)Orn^2^- _L_-AcN(OH)Orn^3^- _L_-Ser-_L_-Leu-_D_-Phe) chelating with Al (III).

Fe (III)-acremonpeptide F (**6**) was obtained as an orange-red solid. NMR spectra indicated the possible presence of Fe (III) in compound **6** as compound **3**. The HRESIMS data of compound **6** displayed an [M + H]^+^ ion at *m*/*z* 917.3520, corresponding to a molecular formula of C_39_H_59_N_9_O_12_Fe, which indicated that compound **6** may be the complex of **4** by chelating Fe (III). Its planar structure was further verified by the ESIMS/MS fragments of *m*/*z* 745.2768 [M‒AcN(OH)Orn + H]^+^, *m*/*z* 658.2411 [M‒AcN(OH)Orn-Ser + H]^+^, *m*/*z* 545.1639 [M‒AcN(OH)Orn-Ser-Leu + H]^+^, and *m*/*z* 398.0899 [M‒AcN(OH)Orn-Ser-Leu-Phe + H]^+^ (Figure 3). The absolute configuration of the amino acid residues of **6** was established by advanced Marfey’s method and a shared biogenesis, confirming _L_-Ser, _L_-Leu, _D_-Phe, and three _L_-AcN(OH)Orn residues in **6**.

Ga (III)-acremonpeptide E (**7**) and Ga (III)-acremonpeptide F (**8**) were obtained as white powders prepared from acremonpeptide E (**2**) and F (**4**) with Ga_2_(SO_4_)_3_·H_2_O, respectively. The structures of compounds **7** and **8** were further identified under the guidance of HRESIMS and NMR data (Figures S54–S60 for **7** and Figures S63–S69 for **8**).

Aselacin D (**9**) was isolated as a faint yellow solid, and its molecular formula was assigned as C_46_H_66_N_8_O_10_ on the basis of HRESIMS. The ^13^C NMR and distortionless enhancement by polarization transfer (DEPT) spectra revealed 46 resonances, including 3 methyl, 17 methylene, 14 methine, 3 quaternary, and 9 carbonyl carbons. The presence of signals in the amide NH and α-amino acid protons in ^1^H NMR spectrum and carbonyl groups of its ^13^C NMR data (Table 3) indicated the peptidic nature of this molecule. Interpretation of the TOCSY and HMBC correlations suggested the existence of Gly, Ala, Trp, Thr, and Gln residues, five common amino acid residues. In addition, two methylene groups (*δ*_H_ 2.54, *δ*_H_ 2.28, and *δ*_C_ 34.2/*δ*_H_ 3.47, *δ*_H_ 3.01, and *δ*_C_ 36.5) coupled to the carbonyl carbon *δ*_C_ 171.7 revealed the presence of a *β*-Ala residue. Whereafter, detailed 1D and 2D NMR data revealed that the remaining proton and carbon signals accounted for an aliphatic chain containing a diene and two carbonyl groups (Table 3). The HMBC correlations of Gly-NH (*δ*_H_ 7.81)/Ala-CO (*δ*_C_ 172.8), Ala-NH (*δ*_H_ 8.97)/Trp-CO (*δ*_C_ 173.6), Trp-NH (*δ*_H_ 7.91)/ *β*-Ala -CO (*δ*_C_ 171.7), *β*-Ala-NH (*δ*_H_ 7.34)/Thr-CO (*δ*_C_ 168.4), Thr-NH (*δ*_H_ 8.40)/Gln-CO (*δ*_C_ 173.0), and Gln-NH (*δ*_H_ 8.45)/fatty acid-CO (*δ*_C_ 173.8) confirmed the sequence of Gly-Ala-Trp-*β*Ala-Thr-Gln in this compound and the connection of aliphatic chain with amino group via the Gln residue. Furthermore, a cyclic depsipeptide, formed between Thr and Gly residues, was supported by the key correlations of Thr-3 (*δ*_H_ 5.40)/Gly (*δ*_C_ 168.2) and Thr-4 (*δ*_H_ 1.05)/Gly (*δ*_C_ 168.2). The absolute configuration of each amino acid residues was confirmed using advanced Marfey’s method. LC–MS analysis of the hydrolysate’s derivatives of each amino acid residue and comparison with the retention times of the standards assigned the _L_-Ala, _L_-Thr, _D_-Trp, and _D_-Gln (detected as _D_-Glu) in **9** (Figure S79). Finally, the configuration of *E*,*E*-diene in aliphatic chain was established from their proton-proton coupling constants of 15.6 Hz.

Aselacin C (**10**) was obtained as a faint yellow solid, and its molecular formula C_46_H_66_N_8_O_11_ was suggested by the HRESIMS data at *m*/*z* 907.4929 [M + H]^+^. ^1^H and ^13^C NMR data indicated that compound **10** shared high similar structural features with compound **9**, except for an oxygen methylene group (*δ*_C_ 60.4) instead of the methyl (*δ*_C_ 16.2) at Ala, suggesting that the Ala in **9** was displaced by Ser residue in **10**. A literature survey indicated **10** to be identical to the known compound aselacin C [16]. The application of advanced Marfey’s analysis supported the absolute configuration as depicted.

Invasive aspergillosis usually leads to a severe life-threatening infection, especially for immunocompromised patients [17,18]. Recently, ASP2397 (also known as VL-2397 under clinical trial), a hydroxamate-containing siderophore isolated from fungus *Acremonium persicinum*, exhibited potent antifungal activities [6,19]. Hydroxamic acids usually possess a formula RC(O)N(OH)R’ and can be regard as a type of N-hydroxy amides [20]. Here, elucidation of acremonpeptide E; acremonpeptide F; and their complexes with Al^3+^, Fe^3+^, and Ga^3+^ (**1**–**8**) further enrich the chemical structural diversity of the hydroxamate siderophore family.

### 2.2. Biological Evaluation of These Compounds

Hydroxamate-containing compounds **1**–**8** were evaluated for antifungal activities against *Aspergillus fumigatus* and *Aspergillus niger*. We found that compounds **1**, **5**, **7**, and **8** showed obvious antifungal activities against *A. fumigatus* and *A. niger* with MIC values ranging from 1 to 3 μM, which is comparable to the positive control amphotericin B (Table S1).

In line with the previous report, the free acremonpeptides or Fe (III)-acremonpeptides failed to show antifungal activities in biological evaluation [6]. We speculate that the tested fungi may use the free acremonpeptides or Fe (III)-acremonpeptides as a vector to take in Fe^3+^ for survival, but this could be blocked by acremonpeptides chelating other ions, which will occupy the transport receptor of absorption.

The cytotoxic assays indicated that compounds **1**–**10** were inactive against non-small cell lung cancer cell line A549, small cell lung cancer cell lines H446 and H1688, and human embryonic lung fibroblast cell MRC-5 at concentrations up to 30 μM. This result is consistent with the deductive mechanism of action for this class of compounds, targeting Sit1 of *Aspergillus* genus, which is lacking in mammalian cells, indicating the potential druggability of these compounds [21]. Aselacins D (**9**) and C (**10**) were not tested for the antifungal activity for the ullage of samples.

## 3. Experimental Section

### 3.1. Fungal Material and Fermentation

The fungal strain *Acremonium persicinum* F10 was isolated from the fresh inner issue of marine sponge *Phakellia fusca* collected at a depth of 10–20 m near the Yongxin Island (112°20′ E, 16°50′ N) in the South China Sea in June 2013. This fungus was identified as *Acremonium persicinum* on the basis of morphological characteristics and sequence analysis of the ITS region (GenBank, accession no. MH882418). A voucher specimen was preserved at Marine Biotechnology Laboratory, School of Life Sciences and Biotechnology, Shanghai Jiao Tong University, Shanghai, China. The fungus *A. persicinum* F10 was cultured on potato dextrose agar (PDA) for 7 days. The spores of *A. persicinum* F10 were inoculated into 50 mL of seed medium (PDB) in a 250 mL Erlenmeyer flask and incubated on a rotary shaker at 25 °C (150 rpm) for 48 h. Then, 5% (*v*/*v*) seed cultures were transferred into 200 mL production medium (rice 80 g/L, peptone 6 g/L in artificial seawater [22] at pH 7.0) in 60 × 1 L Erlenmeyer flasks under static conditions at room temperature. 

### 3.2. Compound Preparation 

After 40 days of fermentation, the rice medium was smashed and extracted by ethyl acetate to yield 50.1 g of ethyl acetate extract. Preparative medium-phased liquid chromatography (MPLC) was performed on a flash purification system (Bonna Agela Technologies Corporation, Tianjin, China). High-performance liquid chromatography (HPLC) was carried out on an Agilent 1200 liquid chromatography system equipped with a diode array detector (DAD) detector. 1-Fluoro-2,4-dinitrophenyl-5-_L_-/_D_-leucinamide (_L_-/_D_-FDLA) and m-chloroperbenzoic acid were purchased from Sigma-Aldrich Chemical Corporation.

The extract was subjected to MPLC with silica gel column eluted by gradients of CH_2_Cl_2_/MeOH (100:0, 98:2, 95:5, 92:8, 90:10, 80:20, 0:100, *v*/*v*) to afford seven fractions, Fr.A1–Fr.A7, respectively. Subsequently, Fr.A4 (CH_2_Cl_2_/MeOH = 92:8) and Fr.A5 (CH_2_Cl_2_/MeOH = 90:10) were further isolated using Sephadex LH-20 column chromatography with methanol as eluent, giving fractions (Fr.A4B1-B3) and (Fr.A5B1-B3), respectively.

The subfraction Fr.A4B2 and Fr.A5B2 were combined and purified by semipreparative HPLC with an RP-C18 column (Eclipse XDB-C18 5 μm, 9.4 × 250 mm) eluting by 25% ACN/H_2_O, at a flow rate of 3.5 mL/min (UV at 210 nm), to obtain six compounds (**1**–**6**): compound **1** (22.3 mg, retention time, *t*_R_ 29.0 min), **2** (105.6 mg, *t*_R_ 20.7 min), **3** (48.2 mg, *t*_R_ 39.0 min), **4** (37.9 mg, *t*_R_ 17.7 min), **5** (24.9 mg, *t*_R_ 31.5 min), and **6** (37.9 mg, *t*_R_ 37.1 min). The eluant was collected, -dried, and further purified by HPLC using 52% ACN/H_2_O (4.0 mL/min) to isolate **9** (1.2 mg, retention time, *t*_R_, 8.9 min) and **10** (13.2 mg, *t*_R_ 10.0 min).

### 3.3. Spectrum Analysis 

UV spectra were measured on a UV–VIS spectrophotometer (UV/EV300, Thermo scientic, Waltham, MA, USA). Optical rotations were recorded on a P-2000 digital polarimeter (Jasco, Japan) with a 1.0 mL cell. IR spectra were recorded on a KBr pellets using a Fourier transform infrared spectrometer (FT-IR) (Nicolet 6700, Thermo Nicolet Co., Waltham, MA, USA). XRF spectra were obtained by X-ray fluorescence spectrometer (XRF-1800, Shimadzu Inc., Tokyo, Japan). Mass spectra were measured on a positive ion mode using LC–HRMS with a Waters ACQUITY UPLC system (Waters Inc., Milford, MA, USA) coupled with a Waters Micromass Q-TOF Premier Mass Spectrometer, which was equipped with an electrospray interface. The NMR data were collected by a Bruker Avance III 600 MHz spectrometer (600 MHz, Bruker Co., Ltd., Karlsruhe, Germany) at 600 MHz for ^1^H nuclei and 150 MHz for ^13^C nuclei. Chemical shifts are expressed in *δ* (ppm) and referenced to the solvent residual peak. 

Al (III)-acremonpeptide E (**1**): white needle crystals; [*α*]${}_{D}^{25}$ + 31.7 (*c* 0.3, MeOH); UV (MeOH) *λ*_max_ (log *ε*): 208 (4.48) nm; IR (KBr) *ν*_max_ 3425, 2965, 1648, 1401 cm^−1^; ^1^H and ^13^C NMR data, Table 1; HRESIMS *m*/*z* 872.4123 [M + H]^+^ (Calcd. for C_39_H_59_N_9_O_12_Al, *m*/*z* 872.4099 [M + H]^+^). 

Acremonpeptide E (**2**): light yellow solid; [*α*]${}_{D}^{25}$ − 14.4 (*c* 1.0, MeOH); UV (MeOH) *λ*_max_ (log *ε*): 212 (4.20) nm; IR (KBr) *ν*_max_ 3424, 2931, 1643, 1527, 1415, 1007, 832 cm^−1^; ^1^H and ^13^C NMR data, Table 1; HRESIMS *m*/*z* 848.4519 [M + H]^+^ (Calcd. for C_39_H_62_N_9_O_12_, *m*/*z* 848.4518 [M + H]^+^).

Fe (III)-acremonpeptide E (**3**): orange needle crystals; [*α*]${}_{D}^{25}$ + 161.0 (*c* 0.3, MeOH); UV (MeOH) *λ*_max_ (log *ε*): 208 (4.12), 428 (2.97) nm; IR (KBr) *ν*_max_ 3443, 2960, 2934, 1646, 1578, 1518, 1452 cm^−1^; ^1^H and ^13^C NMR data, Table 1; HRESIMS *m*/*z* 901.3745 [M + H]^+^ (Calcd. for C_39_H_59_N_9_O_12_Fe, *m*/*z* 901.3633 [M + H]^+^).

Acremonpeptide F (**4**): light yellow solid; [*α*]${}_{D}^{25}$ − 0.60 (*c* 0.5, MeOH); UV (MeOH) *λ*_max_ (log *ε*): 206 (4.11) nm; IR (KBr) *ν*_max_ 3425, 2960, 2928, 1646, 1519, 1401, 1007, 832 cm^−1^; ^1^H and ^13^C NMR data, Table 2; HRESIMS *m*/*z* 864.4471 [M + H]^+^ (Calcd. for C_39_H_62_N_9_O_13_, *m*/*z* 864.4677 [M + H]^+^).

Al (III)-acremonpeptide F (**5**): white needle crystals; [*α*]${}_{D}^{25}$ + 66.6 (*c* 0.5, MeOH); UV (MeOH) *λ*_max_ (log *ε*): 206 (4.19) nm; IR (KBr) *ν*_max_ 3425, 2960, 2928, 1646, 1519, 1401, 1007, 832 cm^−1^; ^1^H and ^13^C NMR data, Table 2; HRESIMS *m*/*z* 888.4073 [M + H]^+^ (Calcd. for C_39_H_59_N_9_O_13_Al, *m*/*z* 888.4048 [M + H]^+^).

Fe (III)-acremonpeptide F (**6**): orane solid; [*α*]${}_{D}^{25}$ + 166.4 (*c* 0.05, MeOH); UV (MeOH) *λ*_max_ (log *ε*): 212 (4.26), 368 (3.86) nm; IR (KBr) *ν*_max_ 3415, 2928, 1630, 1551, 1401, 1007, 832 cm^−1^; ^1^H and ^13^C NMR data, Table 2; HRESIMS *m*/*z* 917.3520 [M + H]^+^ (Calcd. for C_39_H_59_N_9_O_13_Fe, *m*/*z* 917.3582 [M + H]^+^).

Ga (III)-acremonpeptide E (**7**): white solid; [*α*]${}_{D}^{25}$ + 105.8 (*c* 0.5, MeOH); UV (MeOH) *λ*_max_ (log *ε*): 210 (4.15) nm; IR (KBr) *ν*_max_ 3425, 2961, 1647, 1517, 1401, 1006, 832 cm^−1^; ^1^H and ^13^C NMR data, Table 1; HRESIMS *m*/*z* 914.3547 [M + H]^+^ (Calcd. for C_39_H_59_N_9_O_12_Ga, *m*/*z* 914.3539 [M + H]^+^).

Ga (III)-acremonpeptide F (**8**): colorless oil; [*α*]${}_{D}^{25}$ + 123.0 (*c* 0.5, MeOH); UV (MeOH) *λ*_max_ (log *ε*): 206 (4.20) nm; IR (KBr) *ν*_max_ 3425, 2962, 1646, 1520, 1401, 1007, 832 cm^−1^; ^1^H and ^13^C NMR data, Table 2; HRESIMS *m*/*z* 930.3498 [M + H]^+^ (Calcd. for C_39_H_59_N_9_O_13_Ga, 930.3488).

Aselacin D (**9**): light yellow solid; [*α*]${}_{D}^{25}$ − 3.0 (*c* 0.05, MeOH); UV (MeOH) *λ*_max_ (log *ε*): 206 (4.25), 274 (4.01) nm; IR (KBr) *ν*_max_ 3425, 2929, 1633, 1553, 1401, 1007, 832 cm^−1^; ^1^H and ^13^C NMR data, Table 3; HRESIMS *m*/*z* 891.4996 [M + H]^+^ (Calcd. for C_46_H_67_N_8_O_10_, *m*/*z* 891.4980 [M + H]^+^).

### 3.4. In Vitro Chelate Compounds Synthesis 

Here, in vitro chelation reactions of ligand compounds **2** and **4** with ferric, gallium, and aluminum ions were carried out. Firstly, 0.5 mL of each aqueous solution (0.01 mmol) of FeCl_3_·6H_2_O, Ga_2_(SO_4_)_3_·H_2_O, and Al_2_(SO_4_)_3_·6H_2_O was stirred at 120 rpm. Then, equivalent volume of compounds **2** or **4** were dissolved in methanol and added in previous aqueous solution to continue stirring for 3 h. Finally, the reaction mixtures were analyzed by UPLC–MS. 

### 3.5. X-ray Crystallographic Analysis

Colorless crystals of **1**, **3**, and **5** were obtained by diffusing n-hexane into a chloroform solution. Single-crystal X-ray diffraction data were collected on a Bruker D8 VENTURE diffractometer using graphite-monochromated Cu K*α* radiation (λ = 1.54178 Å). The parameters in Common Intermediate Format (CIF) format for **1**, **3**, and **5** are available from the Cambridge Crystallographic Data Center (CCDC) under the deposition number CCDC 1906911 for **1**, 1906910 for **3**, and 1906912 for **5**, respectively (accessed on April 1, 2019). Copies of the data can be obtained free of charge via the Internet at www.ccdc.cam.ac.uk, accessed on 14 May 2021.

Crystal data for compound **1**: The molecular structure comprises one molecule of **1** and two molecules of water. C_39_H_58_AlN_9_O_12_, 2 (H_2_O), *M* = 903.92, white (block), monoclinic, space group *P*2(1), *a* = 12.2490 (3) Å, *b* = 11.9399 (3) Å, *c* = 16.7735 (5) Å, crystal size: 0.60 × 0.50 × 0.40 mm. *V* = 2410.97 (11) Å^3^, *Z* = 2, μ (Cu K*α*) = 0.960 mm^−1^, and *F*(000) = 320.0, 36,004 reflections measured, of which 8023 unique (*R*_int_ (*R* factor for symmetry-equivalent intensities) = 0.0399) were used in all calculations. The final R indices (all data) gave *R*_1_ = 0.0419, w*R*_2_ = 0.1176, and the Flack parameter = −0.10 (5).

Crystal data for compound **3***:* The molecular structure comprises one molecule of **3** and two molecules of water. C_39_H_58_FeN_9_O_12_, 2 (H_2_O), *M* = 932.79, orange (block), monoclinic, space group *P*2(1), *a* = 12.1986 (5) Å, *b* = 11.9950 (5) Å, *c* = 16.7889 (7) Å, crystal size: 0.50 × 0.40 × 0.30 mm. *V* = 2419.29 (17) Å^3^, *Z* = 2, μ (Cu K*α*) = 3.088 mm^−1^, and *F*(000) = 986.0, 28,046 reflections measured, of which 7271 unique (*R*_int_ (*R* factor for symmetry-equivalent intensities) = 0.0290) were used in all calculations. The final R indices (all data) gave *R*_1_ = 0.0802, w*R*_2_ = 0.2329, and the Flack parameter = 0.04(8).

Crystal data for compound **5***:* The molecular structure comprises one molecule of **5**. C_39_H_58_AlN_9_O_13_, *M* = 888.40, colorless (block), monoclinic, space group *P*2(1), *a* = 12.207 (3) Å, *b* = 12.049 (4) Å, *c* = 16.512 (7) Å, crystal size: 0.22 × 0.20 × 0.18 mm. *V* = 2392.3(14) Å^3^, *Z* = 2, μ (Cu K*α*) = 0.942 mm^−1^, and *F*(000) = 944.0, 17,328 reflections measured, of which 5253 unique (*R*_int_ (*R* factor for symmetry-equivalent intensities) = 0.0290) were used in all calculations. The final R indices (all data) gave *R*_1_ = 0.1164, w*R*_2_ = 0.2371, and the Flack parameter = 0.02 (6).

### 3.6. Acid Hydrolysis of Compounds ***1**–**6***, and ***9***

Approximately 0.6 mg of each of compounds **1**–**6** was hydrolyzed with 6 N HCl (1 mL) for 16 h at 110 °C. For analysis of compound **9**, 3.0% *v*/*v* triisopropylsilane was added, and the mixture was hydrolyzed with 6 N HCl (1 mL) for 2 h at 110 °C. After cooling to room temperature, the hydrolysate mixtures and traces of HCl were evaporated to dryness.

### 3.7. Absolute Configurations of Amino Acids by the Advanced Marfey’s Analysis

Each acid hydrolysate was resuspended in 100 μL of H_2_O. To each half portion (50 μL), we added 1 N NaHCO_3_ (20 μL) and 100 μL of _L_-FDLA (10 mg/mL in acetone). Each mixture was heated to 50 °C for 1 h. The reaction was quenched with 20 μL of 1 N HCl and dried under nitrogen. The residue was redissolved in MeOH and measured by UPLC–MS using an Acquity UPLC BEH C18 column (2.1 × 50 mm, 1.7 μm, 0.5 mL/min). MeCN/H_2_O containing 0.1% formic acid was used as mobile phase with a linear gradient from 10% to 100% over 12 min. Through comparison of the retention times of the _L_- FDLA derivatives of amino acid standard and corresponding amino acids from each compound, we established the absolute configuration (Figures S11, S22, S39, and S79).

### 3.8. Antifungal Activity Assay 

Compounds **1**–**8** were tested for antifungal activities against *Aspergillus fumigatus* ATCC204305 and *Aspergillus niger* ATCC16404 using broth microdilution antifungal susceptibility testing, according to previously published protocol [23,24]. In the experiments, the RPMI-1640 medium containing 10.4 g/L of RPMI-1640 medium (R8755; Sigma, St. Louis, MO, USA), 6.7 g/L of Yeast Nitrogen Base (YNB; Becton, Dickinson and Company, Franklin Lakes, NJ, USA), 1.8% (*w*/*v*) glucose, and 40 mM HEPES (pH = 7.1) was used as culture medium. The inoculum per well for a 96-well microplate was 2 × 10^4^ fresh conidia/mL (150 µL/well), which were incubated for 24 h at 37 ℃. The minimal inhibitory concentration (MIC) in the assay were performed using resazurin at 0.002% (*w*/*v*) of final concentration. Amphotericin B was used as the positive control, exhibiting MICs at 1 μg/mL to both tested fungal strains.

### 3.9. Cytotoxicity Assay

The cytotoxic activity of compounds **1**–**10** against A549, H466, H1688, and MRC-5 cells (all cell lines were obtained from the Cell Bank of the Chinese Academy of Sciences, Shanghai, China) were evaluated by the MTT assay as described previously [25]. Cisplatin was used as the positive control with IC_50_ values of 3.8 μM, 1.9 μM, 3.0 μM, and 23.8 μM, respectively.

## 4. Conclusions

The organic extract of the sponge-derived fungus *Acremonium persicinum* F10 yielded two types of cyclopeptides, including six new hydroxamate siderophore cyclohexapeptides, acremonpeptides E, acremonpeptides F, and their chelates (**1**–**6**); a new cyclic pentapeptolide, aselacin D (**9**); and a known compound, aselacin C (**10**). Analyses of the spectroscopic data of NMR and HRESIMS elucidated the planar structures of these compounds, while the absolute configurations were confirmed using the advanced Marfey’s method and X-ray single-crystal diffraction analysis.

The differences among the new isolated acremonpeptide analogues **2** and **4** and the previously reported findings were varying amino acid replacements or simply different stereocenters (Figure S1), indicating the substrate tolerance of the biosynthesis pathway of the hydroxamate-based peptidyl siderophore from *Acremonium persicinum*.

The compouds **1**, **5**, **7**, and **8** displayed high anti-fungal activities in vitro against *Aspergillus fumigatus* and *Aspergillus niger*, with MICs at 1 μg/mL. Meanwhile, all these compounds showed no cytotoxicity to normal cell (human embryonic lung fibroblast, MRC-5) at the concentration of 30 μM.

## Figures and Tables

**Table 1:** ^13^C NMR (150 MHz, DMSO-*d*_6_) data and ^1^H NMR (600 MHz, DMSO-*d*_6_) data for Al (III)-acremonpeptide E (**1**), acremonpeptide E (**2**), and Ga (III)-acremonpeptide E (**7**).

		1		2		7	
Unit	Pos.	*δ*_C_, Type	*δ*_H_, mult. (*J* in Hz)	*δ*_C_, type	*δ*_H_, mult. (*J* in Hz)	*δ*_C_, Type	*δ*_H_, mult. (*J* in Hz)
Ala	1	171.1, CO		172.5, CO		171.2, CO	
	2	46.7, CH	4.05, m	49.5, CH	4.01, m	46.9, CH	4.05, m
	3	18.9, CH_3_	1.18, d (6.6)	18.1, CH_3_	1.24, d (7.2)	18.9, CH_3_	1.18, d (6.6)
	2-NH		7.49, d (4.8)		7.81, d (6.0)		7.49, d (4.8)
Leu	1	172.3, CO		172.2, CO		172.4, CO	
	2	53.7, CH	3.87, m	52.6, CH	4.12, m	53.9, CH	3.88, m
	3	39.3, CH_2_	1.23, s; 1.18, s	41.2, CH_2_	1.39, m; 1.34, m	39.3, CH_2_	1.23, s; 1.18, s
	4	23.3, CH	0.76, s	24.4, CH	1.02, m	23.3, CH	0.76, s
	5	21.9, CH_3_	0.76, s	22.3, CH_3_	0.73, d (6.6)	21.9, CH_3_	0.76, s
	6	23.2, CH_3_	0.59, s	23.6, CH_3_	0.66, d (6.6)	23.2, CH_3_	0.59, s
	2-NH		8.27, s		7.25, d (2.4)		8.29, s
Phe	1	169.7, CO		171.3, CO		169.6, CO	
	2	55.6, CH	4.34, m	56.3, CH	4.34, m	55.7, CH	4.25, m
	3	35.9, CH_2_	3.35, m 2.70, m	36.5, CH_2_	3.02, dd (13.8, 6.0) 2.81, dd (13.8, 9.6)	35.9, CH_2_	3.35, m 2.70, t (13.2)
	4	139.0, C		138.1, C		139.0, C	
	5	128.9, CH	7.26, m	129.5, CH	7.26, m	129.0, CH	7.27, m
	6	128.0, CH	7.25, m	128.5, CH	7.25, m	128.1, CH	7.25, m
	7	126.7, CH	7.19, m	126.7, CH	7.19 m	126.1, CH	7.18, m
	8	128.0, CH	7.25, m	128.5, CH	7.25, m	128.1, CH	7.25, m
	9	128.9, CH	7.26, m	129.5, CH	7.26, m	129.0, CH	7.27, m
	2-NH		8.95, d (8.4)		8.79, d (6.0)		8.95, d (8.4)
AcN(OH) Orn-1	1	169.1, CO		171.9, CO		169.1, CO	
	2	52.2, CH	4.79, m	53.2, CH	4.11, m	52.3, CH	4.81, m
	3	24.6, CH_2_	1.85, m; 1.69, m	28.7, CH_2_	1.82, m; 1.38, m	24.8, CH_2_	1.83, m; 1.72, m
	4	20.8, CH_2_	1.17, d (6.6)	23.6, CH_2_	1.50, m; 1.59, m	20.8, CH_2_	1.18, d (6.6)
	5	48.3, CH_2_	3.69, m; 3.18, d (13.8)	46.9, CH_2_	3.40, m	48.9, CH_2_	3.75, m; 3.23, d (13.8)
	6	161.5, CO		170.8, CO		161.3, CO	
	7	15.9, CH_3_	2.05, s	20.8, CH_3_	1.98, m	16.6, CH_3_	2.09, s
	2-NH		8.44, d (8.4)		8.47, d (8.4)		8.45, d (8.4)
AcN(OH) Orn-2	1	174.6, CO		172.5, CO		174.5, CO	
	2	57.8, CH	4.22, m	52.8, CH	4.29, m	57.9, CH	4.20, m
	3	24.4, CH_2_	2.70, m; 1.70, m	29.3, CH_2_	1.68, m	24.7, CH_2_	2.58, m; 1.72, m
	4	26.2 CH_2_	1.95, m; 1.59 t (12.6)	24.0, CH_2_	1.59, m	26.2 CH_2_	1.95, m; 1.61, t (12.0)
	5	48.4, CH_2_	4.02, m; 3.69 m	47.1, CH_2_	3.55, m	49.1, CH_2_	4.07, m; 3.75 m
	6	161.7, CO		170.8, CO		161.6, CO	
	7	16.2, CH_3_	2.09, s	20.8, CH_3_	1.98, m	16.9, CH_3_	2.13, s
	2-NH		10.10, d (6.0)		7.73, d (8.4)		10.07, d (6.0)
AcN(OH) Orn-3	1	169.4, CO		171.8, CO		169.4, CO	
	2	52.5, CH	4.07, m	55.7, CH	3.75, s	52.4, CH	4.11, m
	3	26.8, CH_2_	2.08, s; 1.04, q (12.0)	28.0, CH_2_	1.59, m	27.2, CH_2_	2.10, m; 1.00, q (13.2)
	4	21.4, CH_2_	1.70, m; 1.49, m	23.4, CH_2_	1.32, m	21.5, CH_2_	1.71, m; 1.49, m
	5	47.3, CH_2_	3.68, m; 3.41, m	47.0, CH_2_	3.49, m	47.9, CH_2_	3.74, m; 3.44, m
	6	161.3, CO		170.8, CO		161.2, CO	
	7	15.3, CH_3_	2.09, s	20.9, CH_3_	1.98, m	16.0, CH_3_	2.13, s
	2-NH		6.28, d (9.0)		8.11, s		6.22, d (9.0)

**Table 2:** ^13^C NMR (150 MHz, DMSO-*d*_6_) data and ^1^H NMR (600 MHz, DMSO-*d*_6_) data for acremonpeptide F (**4**), Al (III)-acremonpeptide F (**5**), and Ga (III)-acremonpeptide F (**8**).

		4		5		8	
Unit	Pos.	*δ*_C_, Type	*δ*_H_, mult. (*J* in Hz)	*δ*_C_, Type	*δ*_H_, mult. (*J* in Hz)	*δ*_C_, Type	*δ*_H_, mult. (*J* in Hz)
Ser	1	169.6, CO		169.6, CO		169.7, CO	
	2	56.2, CH	4.03, m	53.2, CH	4.06, m	53.2, CH	4.06, m
	3	61.0, CH_2_	3.65, m; 3.58, m	60.8, CH_2_	3.75, m; 3.34, m	60.7, CH_2_	3.78, m; 3.34, m
	3-OH		5.04, brs		5.03, brs		5.05, brs
	2-NH		7.58, s		7.34, d (4.8)		7.34, d (4.2)
Leu	1	171.8, CO		172.6, CO		172.6, CO	
	2	51.9, CH	4.16, m	53.7, CH	3.91, m	53.6, CH	3.92, m
	3	41.1, CH_2_	1.45, m; 1.30, m	39.4, CH_2_	1.23, s; 1.18, s	39.3, CH_2_	1.22, s; 1.18, s
	4	24.1, CH	1.00, m	23.4, CH	0.75, s	23.4, CH	0.76, s
	5	21.8, CH_3_	0.71, d (6.6)	21.9, CH_3_	0.75, s	21.9, CH_3_	0.76, s
	6	23.3, CH_3_	0.65, d (6.6)	23.2, CH_3_	0.59, s	23.2, CH_3_	0.59, s
	2-NH		7.07, s		7.98, d (3.0)		7.97, d (3.0)
Phe	1	171.1, CO		169.5, CO		169.5, CO	
	2	55.9, CH	4.34, m	55.8, CH	4.24, m	55.9, CH	4.25, m
	3	36.0, CH_2_	2.97 m; 2.82, m	36.0, CH_2_	3.35, m; 2.71, m	35.9, CH_2_	3.37, m; 2.72, m
	4	137.4, C		138.9, C		138.9, C	
	5	129.1, CH	7.25, m	129.0, CH	7.27, m	128.9, CH	7.27, m
	6	128.1, CH	7.24, m	128.1, CH	7.26, m	128.1, CH	7.26, m
	7	126.3, CH	7.18 m	126.1, CH	7.18, m	126.1, CH	7.19, m
	8	128.1, CH	7.24, m	128.1, CH	7.26, m	128.1, CH	7.26, m
	9	129.1, CH	7.25, m	129.0, CH	7.27, m	129.0, CH	7.27, m
	2-NH		8.87, d (6.0)		9.03, d (8.4)		9.04, d (7.2)
AcN(OH) Orn-1	1	171.8, CO		169.1, CO		169.1, CO	
	2	52.9, CH	4.06, m	52.3, CH	4.78, m	52.3, CH	4.80, m
	3	28.1, CH_2_	1.79, m; 1.38, m	24.5, CH_2_	1.81, m; 1.69, m	24.7, CH_2_	1.80, m; 1.72, m
	4	23.0, CH_2_	1.32, m	20.7, CH_2_	1.62, m; 1.23, m	20.7, CH_2_	1.64, m; 1.24, m
	5	46.4, CH_2_	3.38, m	48.5, CH_2_	4.03, m; 3.60, m	49.1, CH_2_	4.08, m; 3.71, m
	6	170.3, CO		161.4, CO		161.3, CO	
	7	20.4, CH_3_	1.97, m	15.9, CH_3_	2.04, s	16.6, CH_3_	2.10, s
	2-NH		8.48, s		8.26, d (7.2)		8.28, d (7.2)
AcN(OH) Orn-2	1	171.1, CO		174.8, CO		174.6, CO	
	2	52.1, CH	4.29, m	57.9, CH	4.23, m	57.9, CH	4.20, m
	3	28.9, CH_2_	1.68, m	24.4, CH_2_	2.69, m; 1.69, m	24.7, CH_2_	2.58, m; 1.72, m
	4	23.7, CH_2_	1.59, m	26.3, CH_2_	1.94, m; 1.59, m	26.2, CH_2_	1.96, m; 1.62, m
	5	46.7, CH_2_	3.55, m	48.4, CH_2_	3.63, m; 3.28, m	49.1, CH_2_	3.71, m; 3.32, m
	6	170.3, CO		161.7, CO		161.6, CO	
	7	20.4, CH_3_	1.98, m	16.2, CH_3_	2.08, s	16.9, CH_3_	2.14, s
	2-NH		7.78, s		10.07, d (6.0)		10.06, d (6.0)
AcN(OH) Orn-3	1	171.7, CO		170.3, CO		170.3, CO	
	2	55.8, CH	3.73, s	52.8, CH	4.09, m	52.7, CH	4.13, m
	3	27.5, CH_2_	1.65, m	27.3, CH_2_	2.03, s; 1.10, m	27.3, CH_2_	2.07, s; 1.07, m
	4	23.2, CH_2_	1.62, m; 1.54, m	21.6, CH_2_	1.70, m; 1.51, m	21.8, CH_2_	1.76, m; 1.52, m
	5	46.5, CH_2_	3.49, m	47.3, CH_2_	3.71, m; 3.41, m	47.9, CH_2_	3.74, m; 3.45, m
	6	170.3, CO		161.3, CO		161.1, CO	
	7	20.4, CH_3_	1.97, m	15.4, CH_3_	2.08, s	16.0, CH_3_	2.14, s
	2-NH		8.31, s		6.33, d (9.0)		6.26, d (9.6)

**Table 3:** ^13^C NMR (150 MHz, DMSO-*d*_6_) data and ^1^H NMR (600 MHz, DMSO-*d*_6_) data for aselacins D and C (**9** and **10**).

		9				10	
Unit	Pos.	*δ*_C_, Type	*δ*_H_, mult. (*J* in Hz)	Unit	Pos.	*Δ*_c_, Type	*δ*_H_, mult. (*J* in Hz)
Gly	1	168.2, CO		Gly	1	168.2, CO	
	2	41.7, CH_2_	3.80, dd (17.4, 6.6); 3.50, dd (17.4, 6.6)		2	41.8, CH_2_	3.84, dd (17.4, 6.6); 3.50, dd (17.4, 6.6)
	2-NH		7.81, t (6.0)		2-NH		7.81, s
Ala	1	172.8, CO		Ser	1	170.6, CO	
	2	49.3, CH	4.00, pent (7.2)		2	56.6, CH	4.07, m
	3	16.2, CH_3_	1.05, t (6.6);		3	60.4, CH_2_	3.75, pent (6.0); 3.42, m
					3-OH		4.92, brs
	2-NH		8.97, d (6.0)		2-NH		9.03, s
Trp	1	173.6, CO		Trp	1	174.2, CO	
	2	54.8, CH	4.39, dd (14.4, 6.6)		2	54.3, CH	4.63, dd (14.4, 6.6)
	3	27.1, CH_2_	3.04, dd (14.4, 6.6); 2.98, dd (14.4, 6.6)		3	27.1, CH_2_	3.07, dd (14.4, 6.6); 2.96, dd (14.4, 6.6)
	4	109.1, C			4	109.1, C	
	5	123.8, CH	7.13, s		5	123.8, CH	7.15, s
	6	136.1, C			6	136.1, C	
	7	111.4, CH	7.31, m		7	111.4, CH	7.34, m
	8	121.0, CH	7.03, t (7.2)		8	121.0, CH	7.06, t (7.2)
	9	118.2, CH	6.96, t (7.2)		9	118.2, CH	6.98, t (7.2)
	10	118.2, CH	7.52, d (9.8)		10	118.4, CH	7.60, d (8.4)
	11	127.2, C			11	127.2, C	
	5-NH		10.92, s		5-NH		10.90, s
	2-NH		7.91, d, (6.0)		2-NH		7.79, d, (6.0)
*β*-Ala	1	171.7, CO		*β*-Ala	1	171.7, CO	
	2	34.2, CH_2_	2.54, m; 2.28, m		2	34.2, CH_2_	2.53, m; 2.27, m
	3	36.5, CH_2_	3.47, m; 3.01, m		3	36.5, CH_2_	3.45 m; 3.06, m
	3-NH		7.34, m		3-NH		7.37, m
Thr	1	168.4, CO		Thr	1	168.4, CO	
	2	55.5, CH	4.46, d (10.2)		2	55.6, CH	4.48, m
	3	69.8, CH	5.40, m		3	69.8, CH	5.42, ddd (13.2, 6.6, 2.4)
	4	16.1, CH_3_	1.05, t (6.6)		4	16.1, CH_3_	1.04, d (6.6)
	2-NH		8.39, d (9.6)		2-NH		8.40, d (9.6)
Gln	1	173.0, CO		Gln	1	173.0, CO	
	2	53.8, CH	4.50, dd (13.2, 6.6)		2	53.8, CH	4.51, m
	3	26.8, CH_2_	1.92, m		3	26.8, CH_2_	1.93, m
	4	31.6, CH_2_	2.16, m		4	31.6 CH_2_	2.15, m
	5	173.3, CO			5	173.3, CO	
	5-NH_2_		6.83, s; 7.34, m		5-NH_2_		6.83, s; 7.34, m
	2-NH		8.46, s, (4.2)		2-NH		8.45, s, (6.0)
Fatty acid	1	173.8, CO		Fatty acid	1	173.8, CO	
	2	34.7, CH_2_	2.18, m		2	34.7, CH_2_	2.18, m
	3	25.1, CH_2_	1.47, m		3	25.1, CH_2_	1.46, m
	4	28.5, CH_2_	1.17-1.20, m		4	28.5, CH_2_	1.18-1.21, m
	5	28.5, CH_2_	1.17-1.20, m		5	28.5, CH_2_	1.18-1.21, m
	6	28.5, CH_2_	1.17-1.20, m		6	28.6, CH_2_	1.18-1.21, m
	7	23.8, CH_2_	1.42, m		7	23.8, CH_2_	1.43, m
	8	39.3, CH_2_	2.47, t (7.2)		8	39.3, CH_2_	2.49, t (7.2)
	9	200.2, CO			9	200.2, CO	
	10	128.1, CH	6.05, d (15.6)		10	128.1, CH	6.08, d (15.6)
	11	142.7, CH	7.13, d (15.6)		11	142.7, CH	7.16, d (15.6)
	12	129.0, CH	6.24, d (15.6)		12	129.0, CH	6.25, d (15.6)
	13	145.4, CH	6.25, d (15.6)		13	145.4, CH	6.26, d (15.6)
	14	32.4, CH_2_	2.18, m		14	32.4, CH_2_	2.14, m
	15	27.9, CH_2_	1.39, m		15	27.9, CH_2_	1.39, m
	16	30.8, CH_2_	1.26, m		16	30.8, CH_2_	1.26, m
	17	21.9, CH_2_	1.27, m		17	21.9, CH_2_	1.28, m
	18	13.9, CH_3_	0.86, t (7.2)		18	13.9, CH_3_	0.86, t (6.6)

## Data Availability

All of the data presented in this study are available in Supplementary Material here.

## Supplementary Materials

The following are available online at https://www.mdpi.com/article/10.3390/md19100537/s1, Table S1. Antifungal activities of acremonpeptide E, acremonpeptides F and their chelates (**1**–**8**), Figure S1. Chemical structures of the previously reported derivatives of ASP2397 isolated from *Acremonium persicinum*, Figure S2. Elemental analysis of compound **1** by X-ray fluorescence (XRF), Figures S3–S13. NMR, HRESIMS, UV, and IR spectra of compound **1**, Figures S14–S24. NMR, HRESIMS, UV, and IR spectra of compound **2**, Figures S25–S30. NMR, HRESIMS, UV, and IR spectra of compound **3**, Figures S31–S41. NMR, HRESIMS, UV, and IR spectra of compound **4**, Figures S42–S50. NMR, HRESIMS, UV, and IR spectra of compound **5**, Figures S51–S53. HRESIMS, UV, and IR spectra of compound **6**, Figures S54–S62. NMR, HRESIMS, UV, and IR spectra of compound **7**, Figures S63–S71. NMR, HRESIMS, UV, and IR spectra of compound **8**, Figures S72–S81. NMR, HRESIMS, UV, and IR spectra of compound **9**.
