# Supplementary material for: Cyclopeptide Derivatives from the Sponge-Derived Fungus Acremonium persicinum F10
Source: Mar Drugs. 2021 Sep 24;19(10):537. doi: 10.3390/md19100537 (PMC8537450; doi:10.3390/md19100537)
Supplement: Supplementary file 1 [file marinedrugs-19-00537-s001.zip › Supporting Information/Supplementary materials-revised version.pdf]

Supplementary Materials

Cyclopeptide Derivatives from the Sponge-Derived Fungus *Acremonium persicinum* F10

Yingxin Li, Zhiyong Li*

Marine Biotechnology Laboratory, State Key Laboratory of Microbial Metabolism and School of Life Sciences and Biotechnology, Shanghai Jiao Tong University, 800 Dongchuan Road, Minhang Shanghai 200240, People's Republic of China.

*Corresponding author: Zhiyong Li

E-mail: zyli@sjtu.edu.cn

Tel: 86- 21- 34204036

Fax: 86- 21- 34204036

CONTENTS

Table S1. Antifungal activities of acremonpeptide E, acremonpeptides F and their chelates (**1-8**) …………………………………………………………………………3
Figure S1. Chemical structures of the previously reported derivatives of ASP2397 isolated from *Acremonium persicinum*……………………………………………………..4
Figure S2. Elemental analysis of compound 1 by X-ray fluorescence (XRF).…….…4
Figure S3-13. NMR, HRESIMS, UV, and IR spectra of compound **1**………………..…..5
Figure S14–24. NMR, HRESIMS, UV, and IR spectra of compound **2**…….………...10
Figure S25–30. NMR, HRESIMS, UV, and IR spectra of compound **3**…………......16
Figure S31–41. NMR, HRESIMS, UV, and IR spectra of compound **4**…………......19
Figure S42-50. NMR, HRESIMS, UV, and IR spectra of compound **5**……………...24
Figure S51–53. HRESIMS, UV, and IR spectra of compound **6**…….………..……...29
Figure S54–62. NMR, HRESIMS, UV, and IR spectra of compound **7**…………......30
Figure S63–71. NMR, HRESIMS, UV, and IR spectra of compound **8**…………......35
Figure S72–81. NMR, HRESIMS, UV, and IR spectra of compound **9**…………......39

Table S1. Antifungal activities of acremonpeptide E, acremonpeptides F and their chelates (**1**–**8**)

Strains	Compounds/MIC (μM)								
	**1**	**2**	**3**	**4**	**5**	**6**	**7**	**8**	Amphotericin B
***A. fumigatus* ATCC204305**	1.0	10.0	>30.0	10.0	1.0	>30.0	1.0	1.0	1.0
***A. niger* ATCC16404**	3.0	30.0	>30.0	30.0	3.0	>30.0	1.0	3.0	1.0

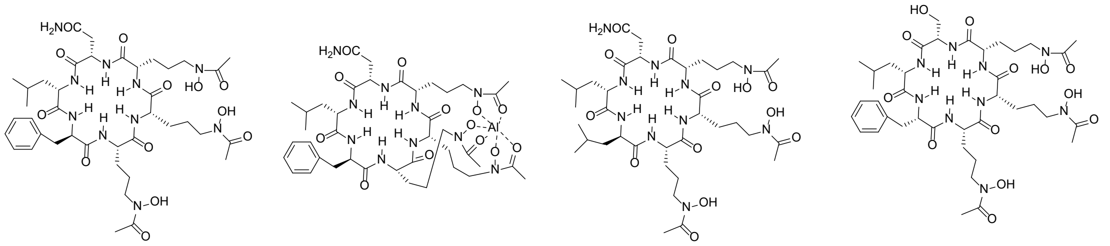

AS2488059 ASP2397 AS2524371 Acremonpeptide A

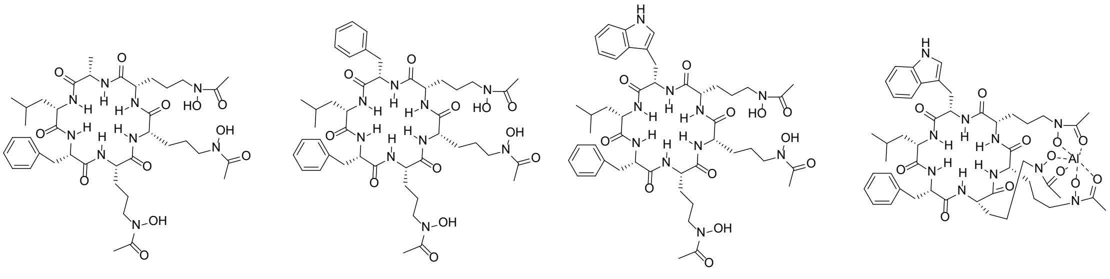

Acremonpeptide B Acremonpeptide C Acremonpeptide D Al (III)-Acremonpeptide D

Figure S1. Chemical structures of the previously reported derivatives of ASP2397 isolated from *Acremonium persicinum*

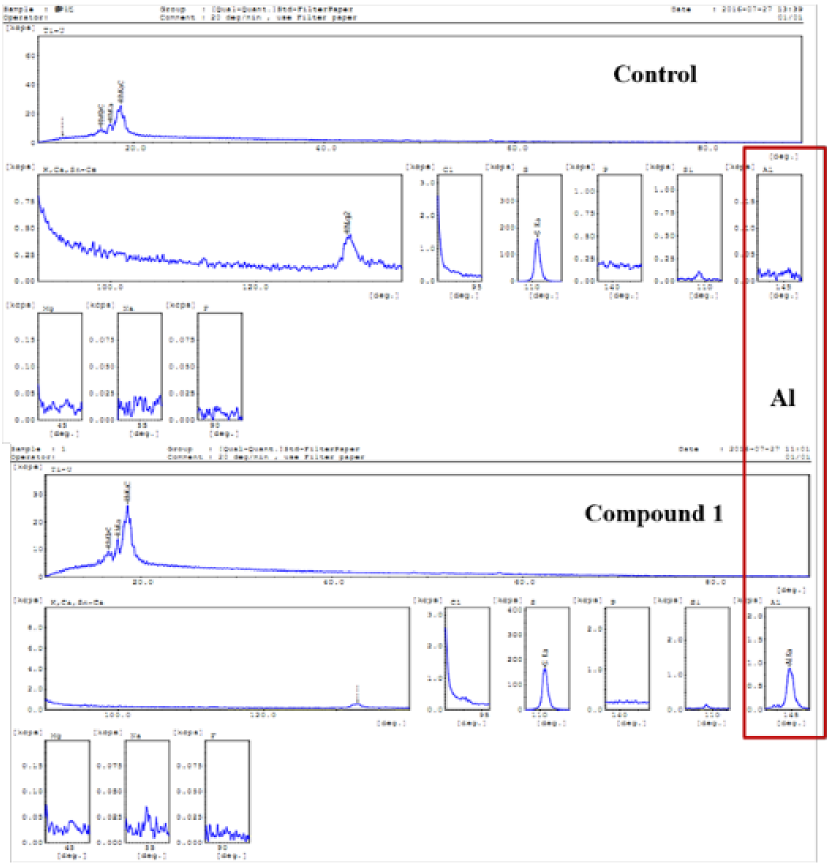

Figure S2. Elemental analysis of compound 1 by X-ray fluorescence (XRF).

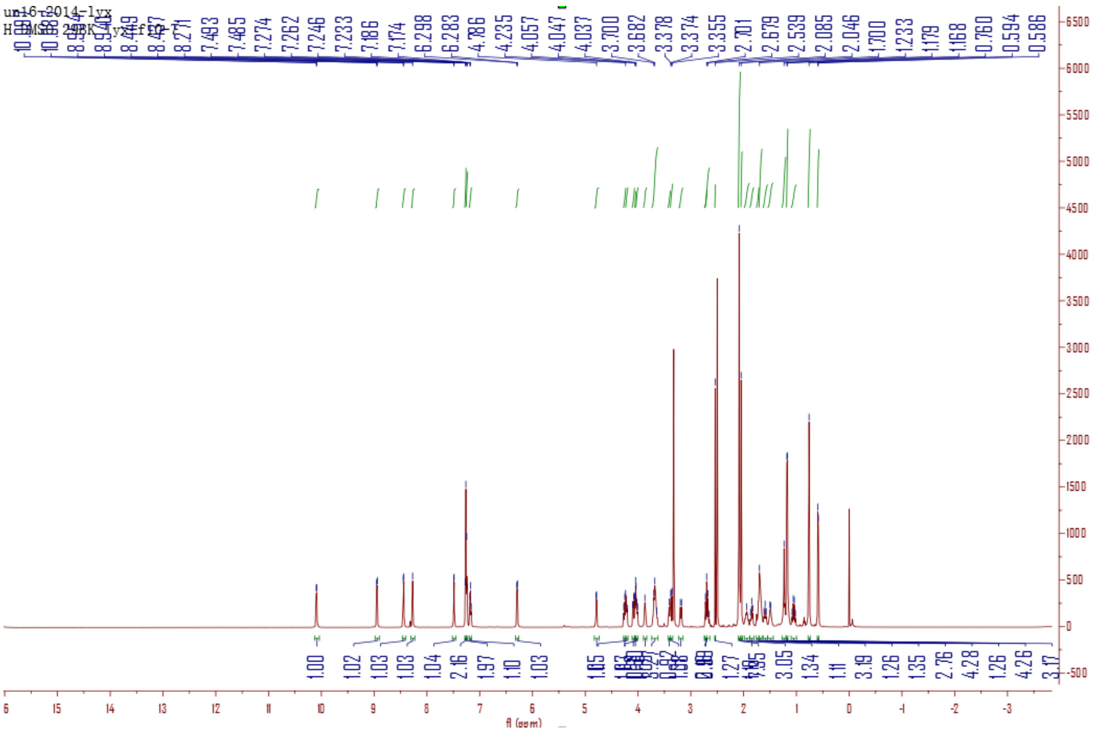

Figure S3. ^{1}H spectrum of Al (III)-acremonpeptide E (**1**) in DMSO-d_6 (600 MHz).

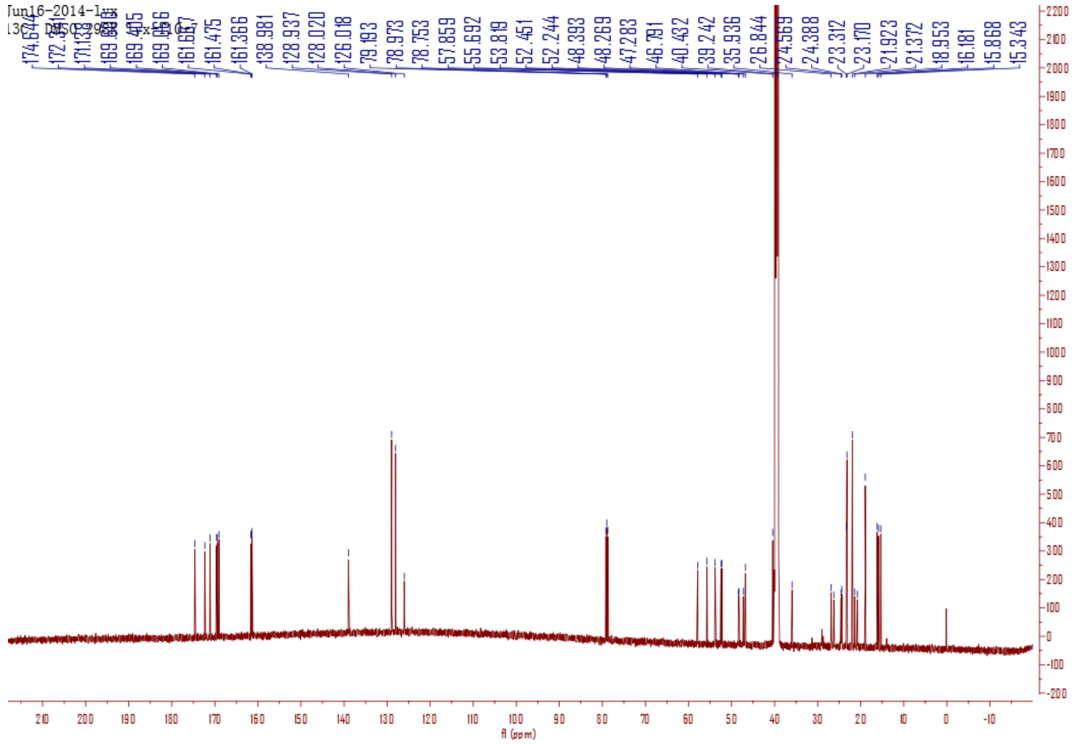

Figure S4. ^{13}C spectrum of Al (III)-acremonpeptide E (**1**) in DMSO-d_6 (150 MHz).

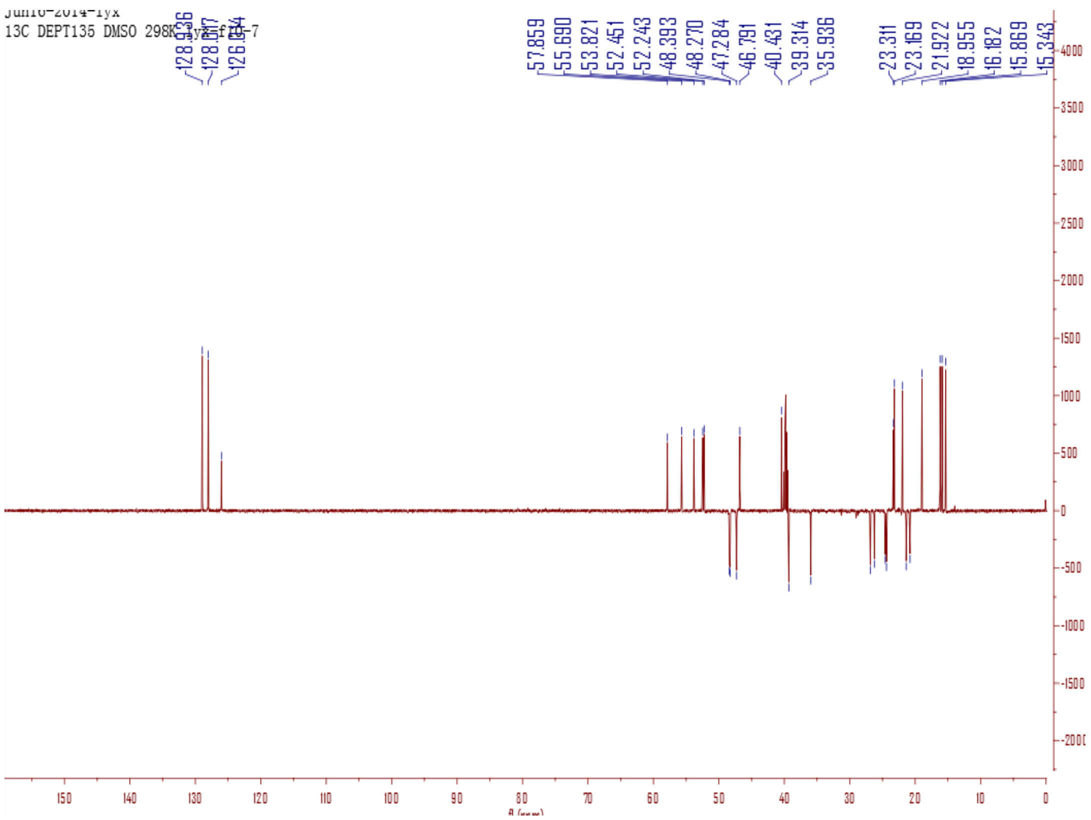

Figure S5. DEPT135 spectrum of Al (III)-acremonpeptide E (**1**) in DMSO-d_6 (150 MHz).

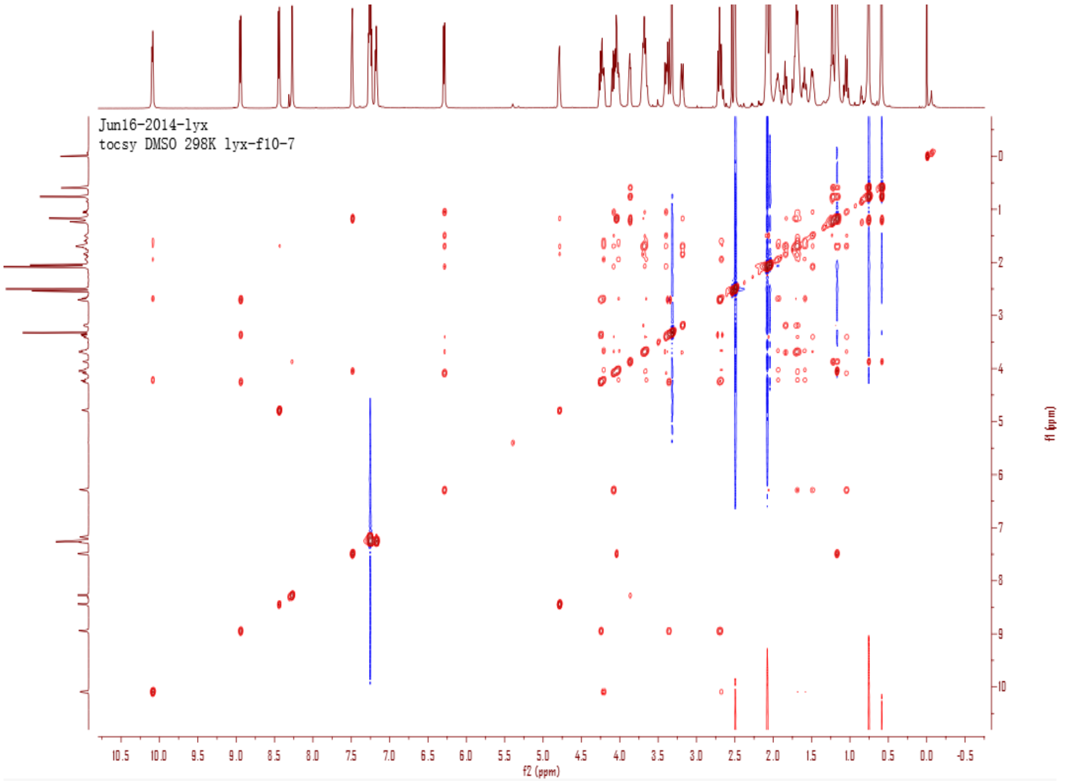

Figure S6. TOCSY spectrum of Al (III)-acremonpeptide E (**1**) in DMSO-d_6

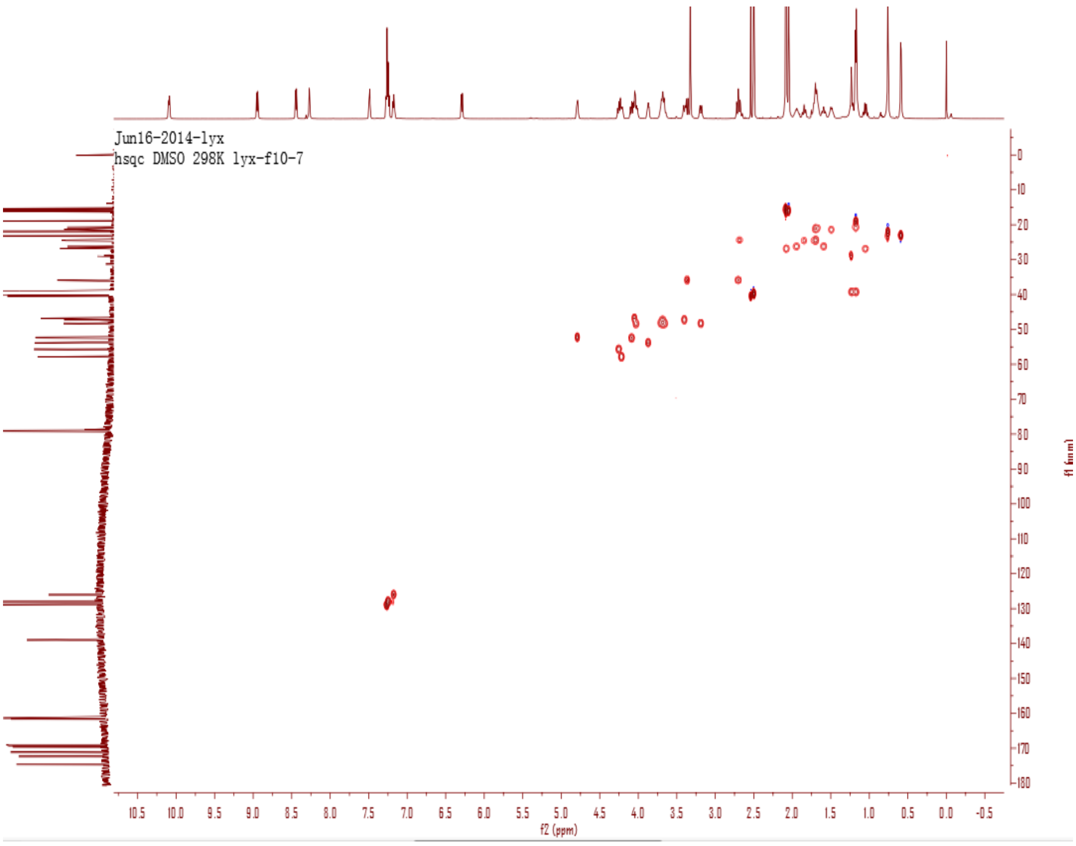

Figure S7. HSQC spectrum of Al (III)-acremonpeptide E (**1**) in DMSO-d_6

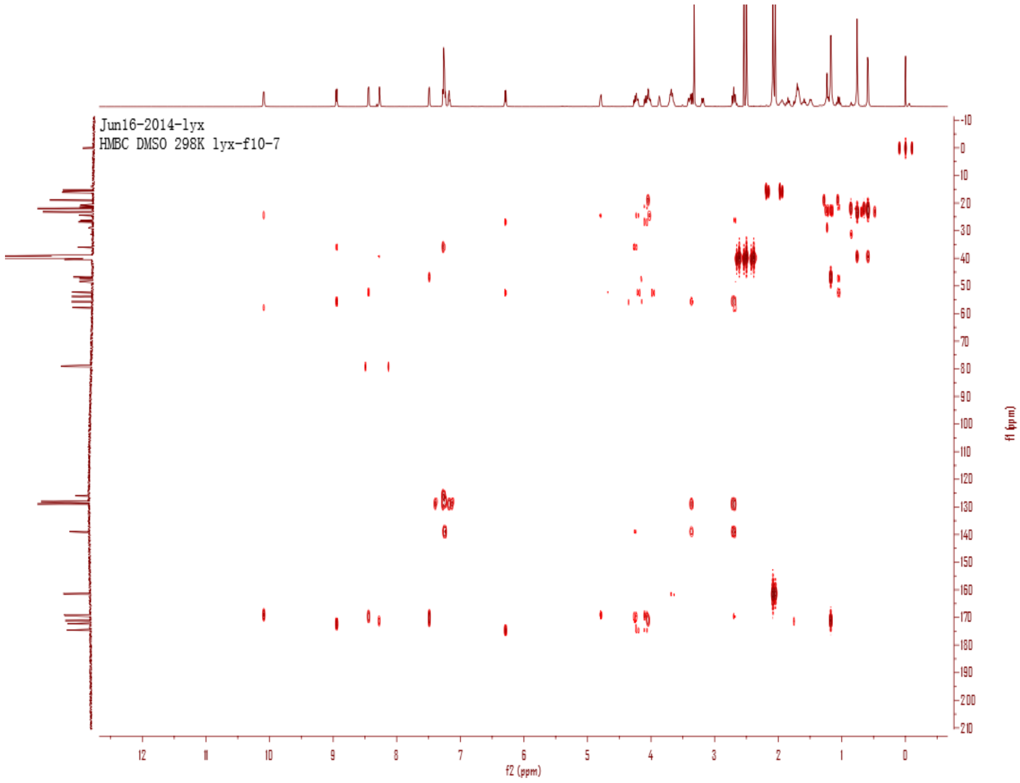

Figure S8. HMBC spectrum of Al (III)-acremonpeptide E (**1**) in DMSO-d_6

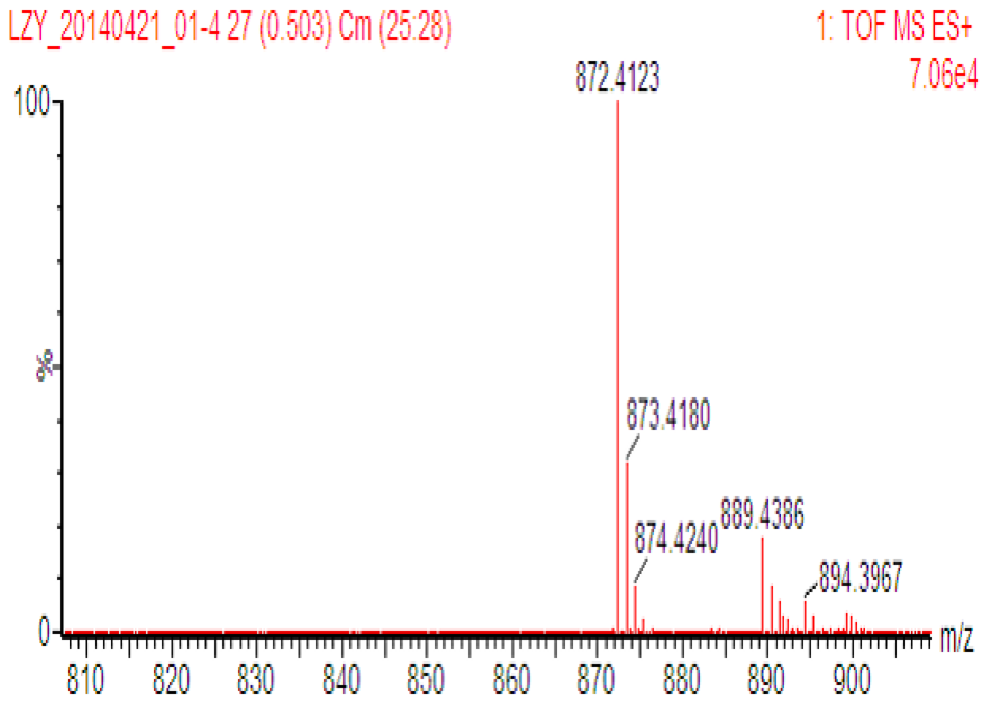

Figure S9. HRESIMS data of Al (III)-acremonpeptide E (**1**)

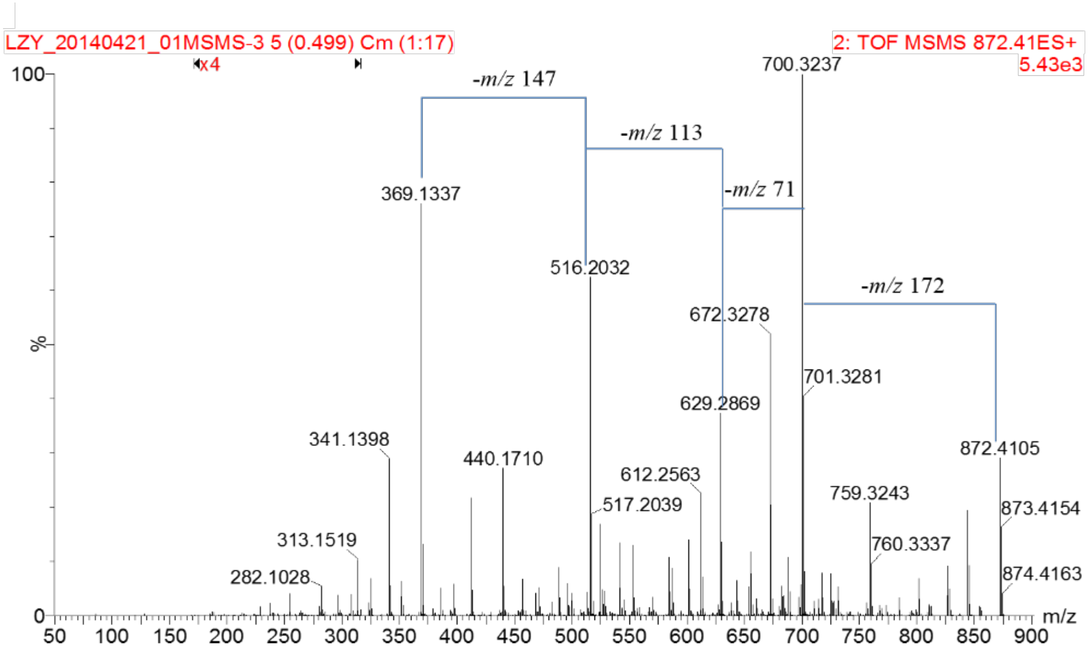

Figure S10. HRESIMS/MS fragmentation ions of Al (III)-acremonpeptide E (**1**)

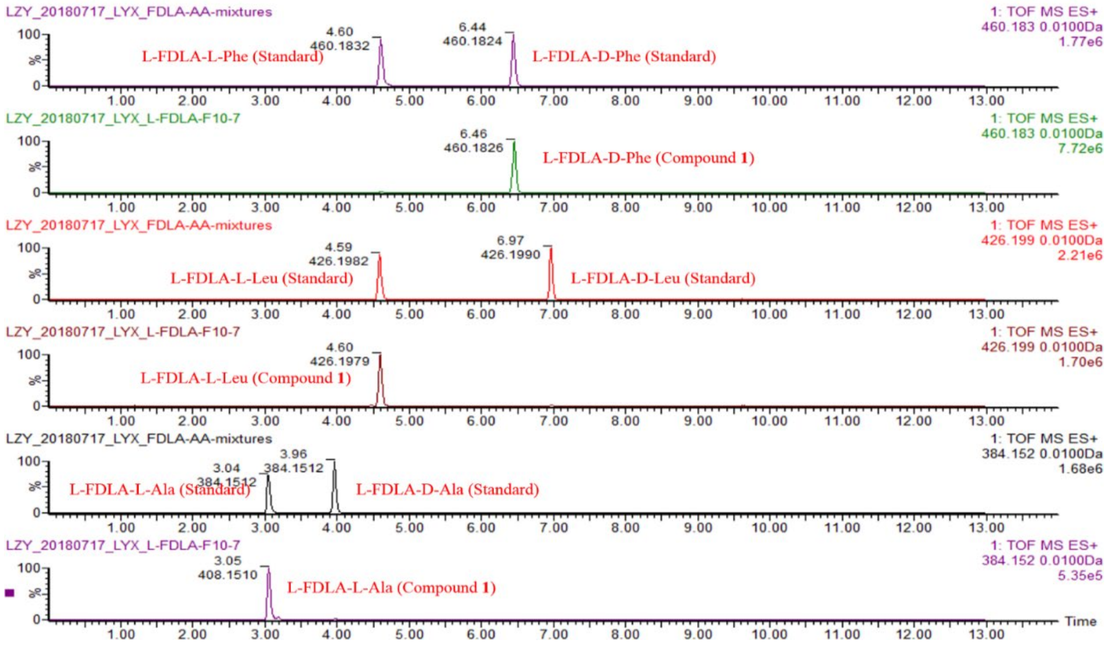

Figure S11. Mass chromatograms of the L-FDLA derivatives of standard amino acids and amino acids from Al (III)-acremonpeptide E (**1**)

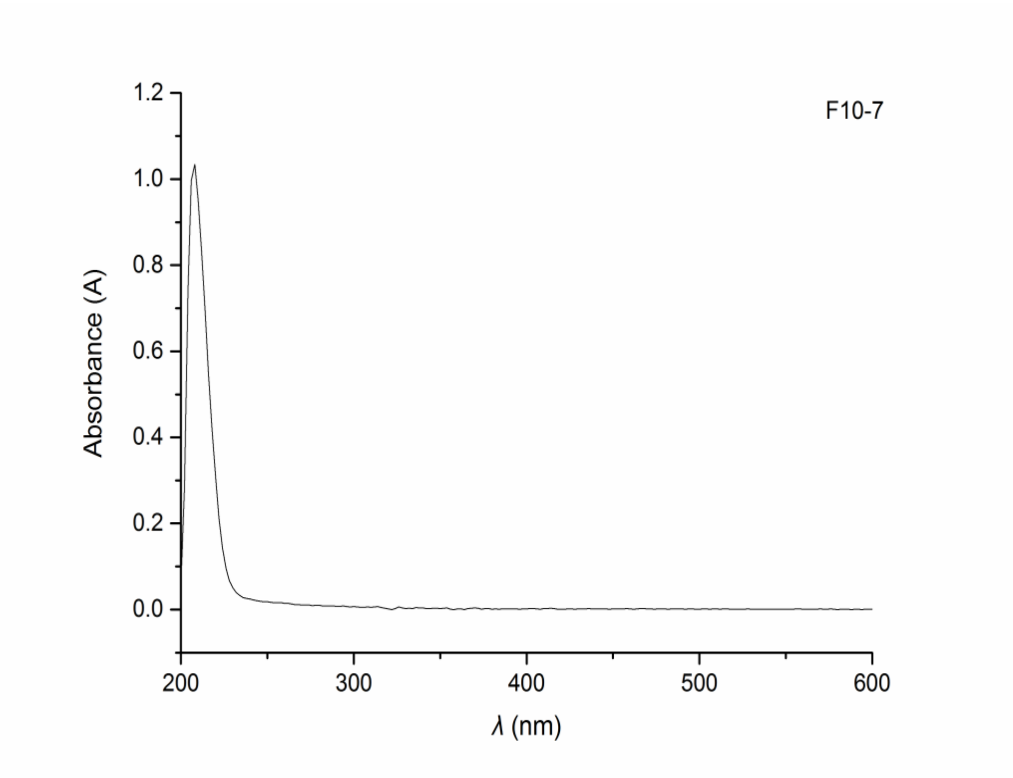

Figure S12. UV spectrum of Al (III)-acremonpeptide E (**1**) in MeOH.

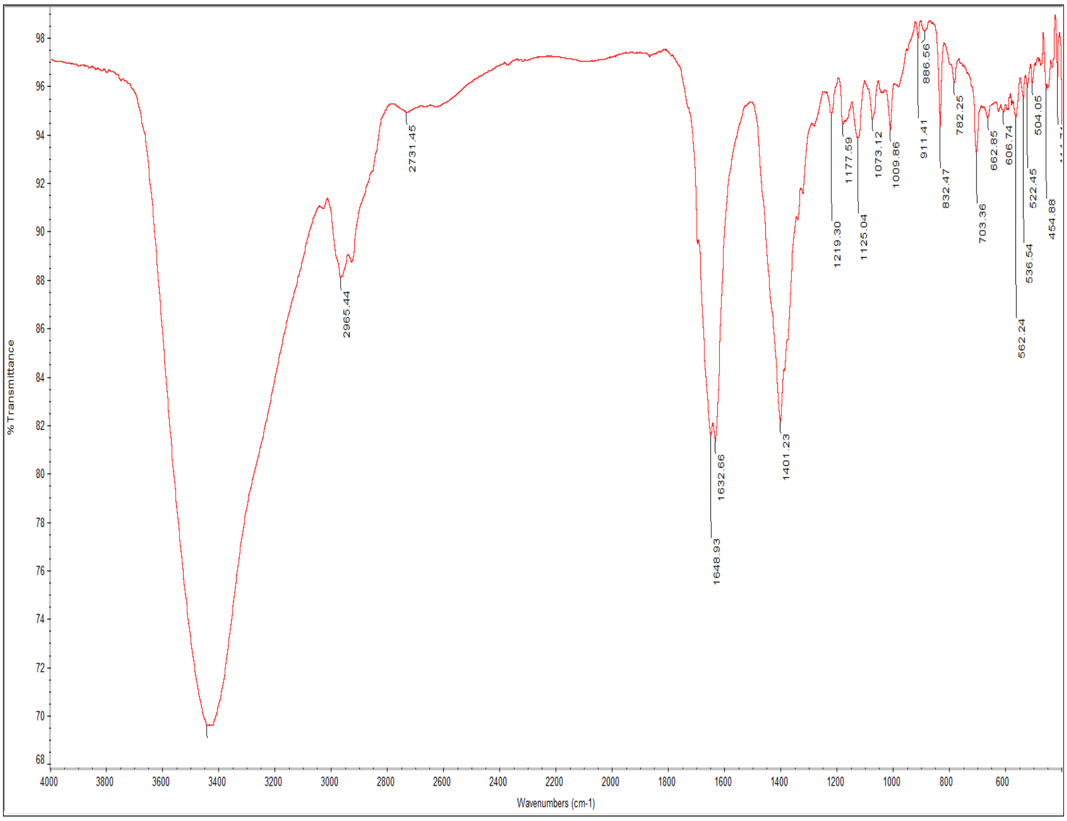

Figure S13. IR spectrum of Al (III)-acremonpeptide E (**1**).

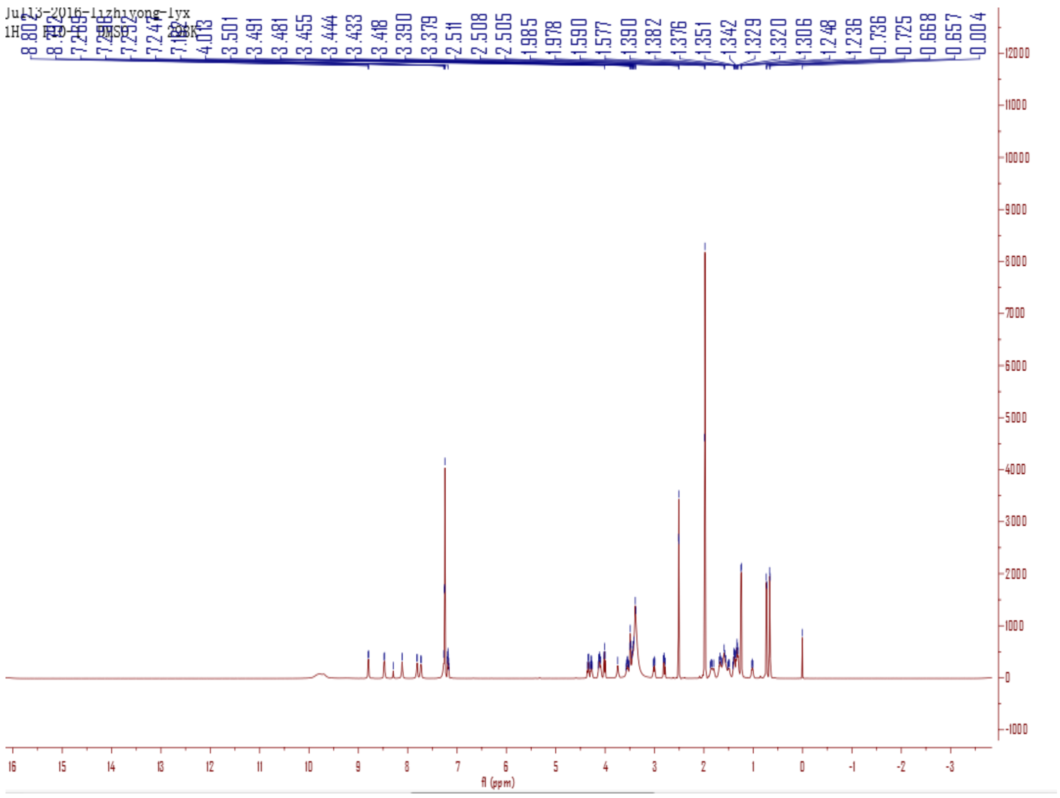

Figure S14. ^{1}H spectrum of acremonpeptide E (**2**) in DMSO-d_6 (600 MHz).

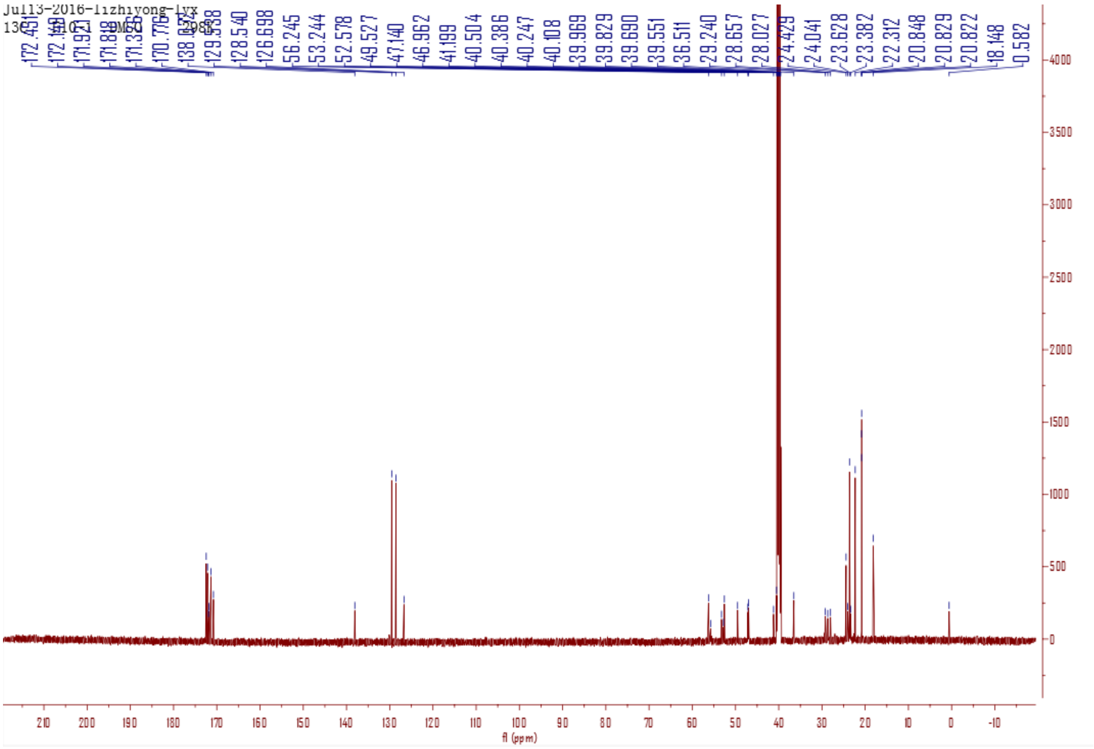

Figure S15. ^{13}C spectrum of acremonpeptide E (**2**) in DMSO-d_6 (150 MHz).

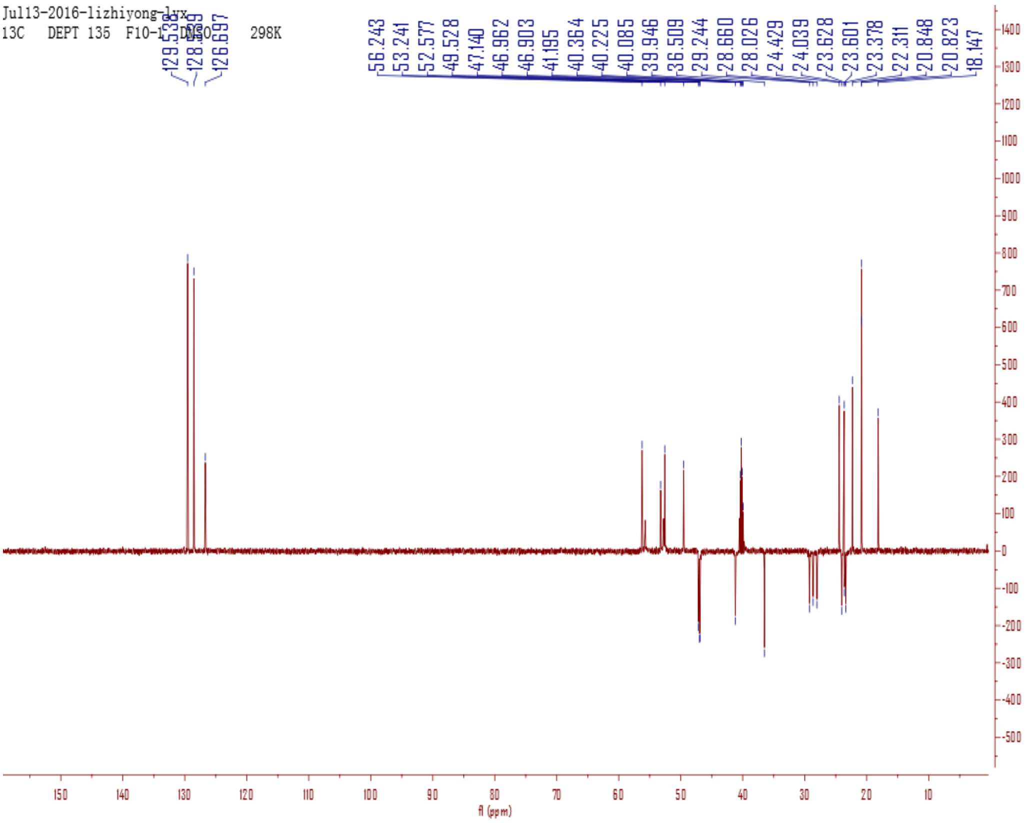

Figure S16. DEPT135 spectrum of acremonpeptide E (**2**) in DMSO-d_6 (150 MHz).

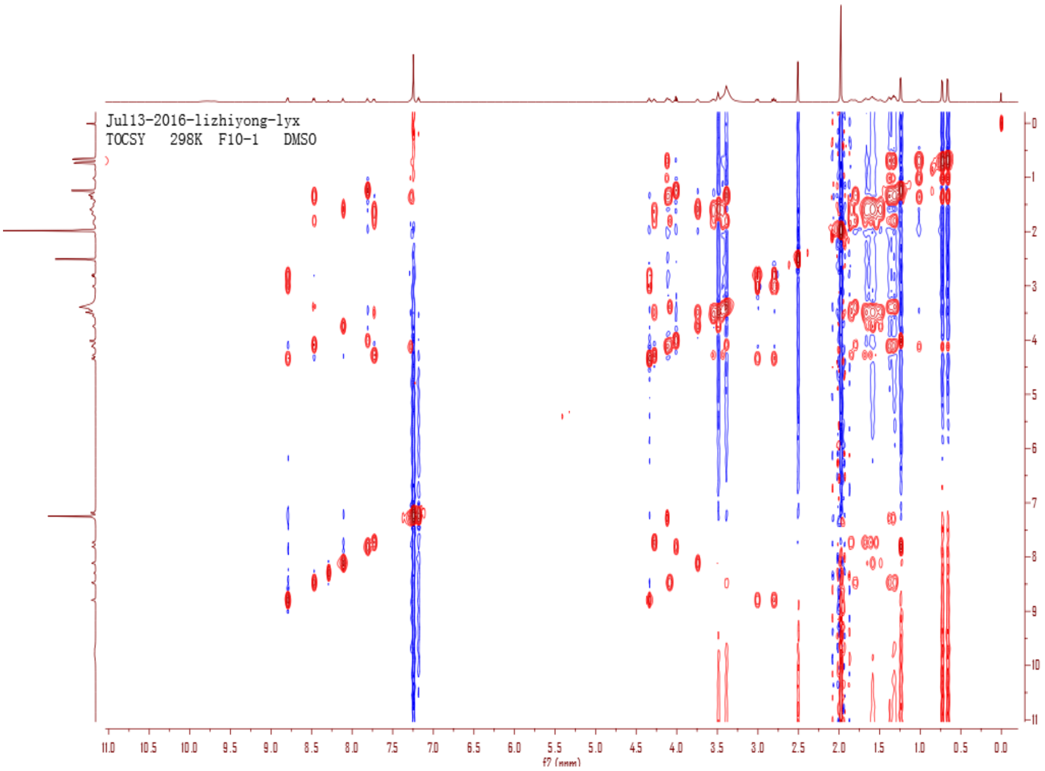

Figure S17. TOCSY spectrum of acremonpeptide E (**2**) in DMSO-d_6.

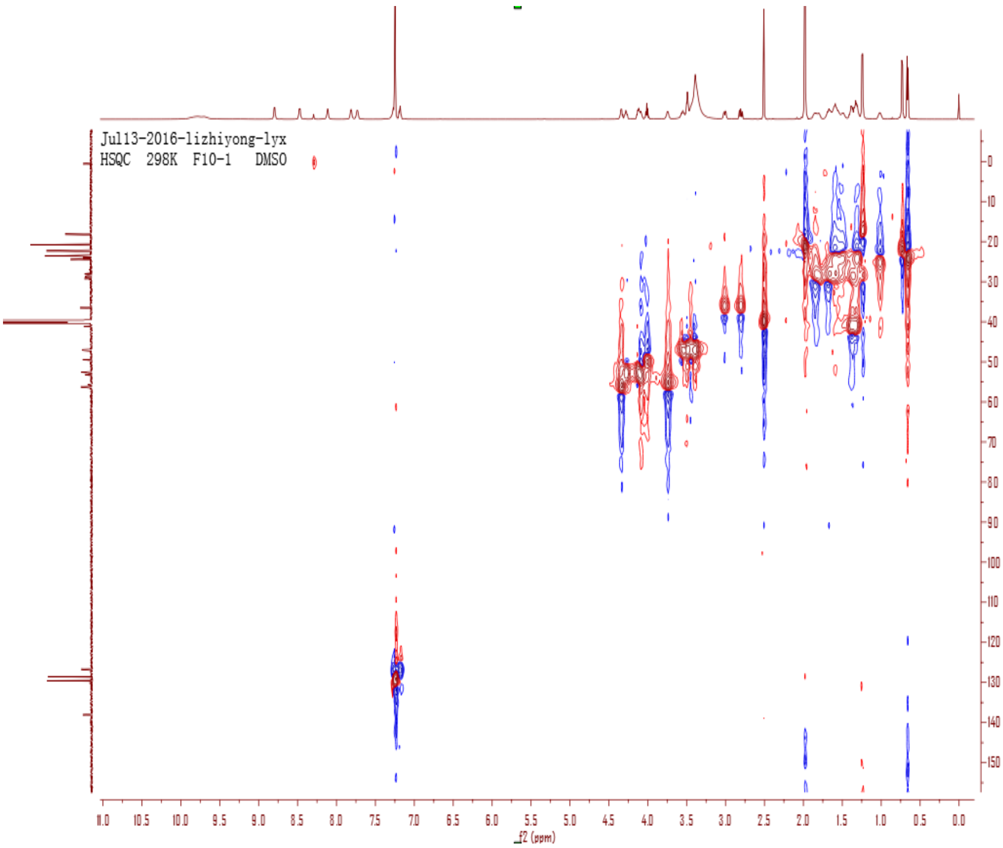

Figure S18. HSQC spectrum of acremonpeptide E (**2**) in DMSO-d_6.

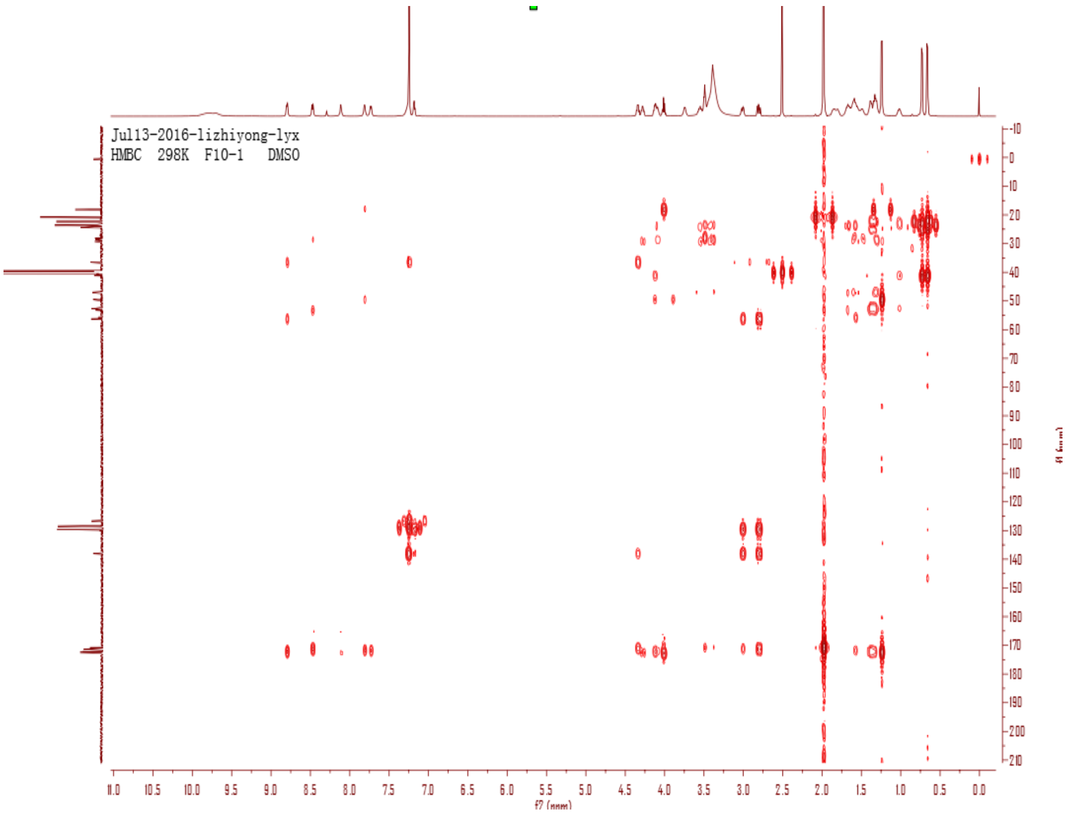

Figure S19. HMBC spectrum of acremonpeptide E (**2**) in DMSO-d_6.

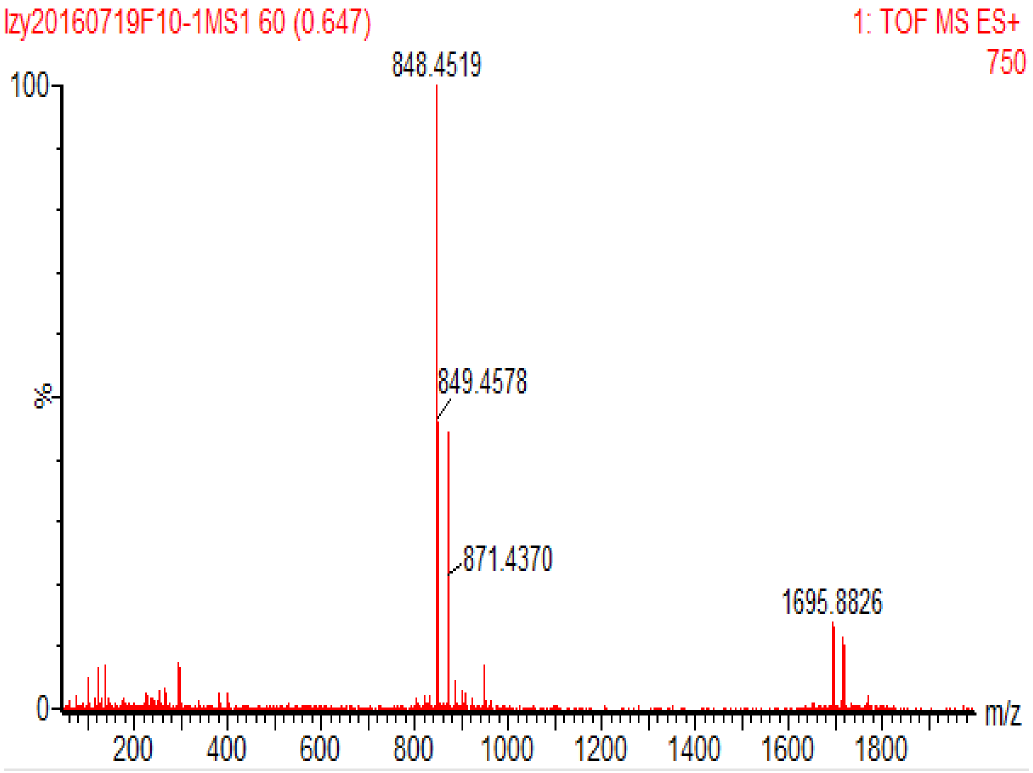

Figure S20. HRESIMS data of acremonpeptide E (**2**).

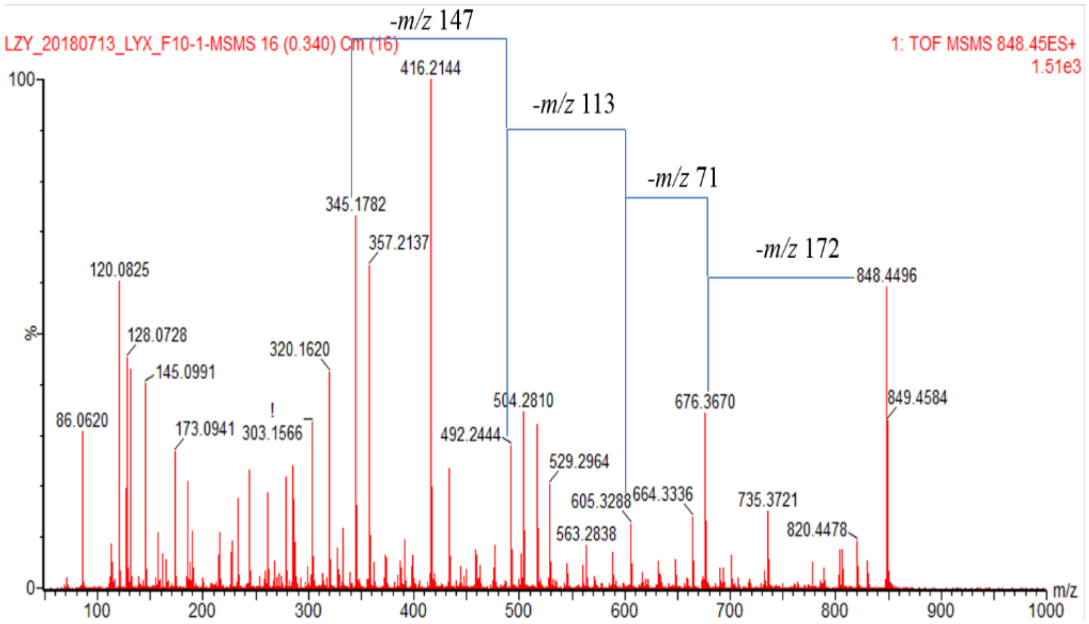

Figure S21. HRESIMS/MS fragmentation ions of acremonpeptide E (**2**)

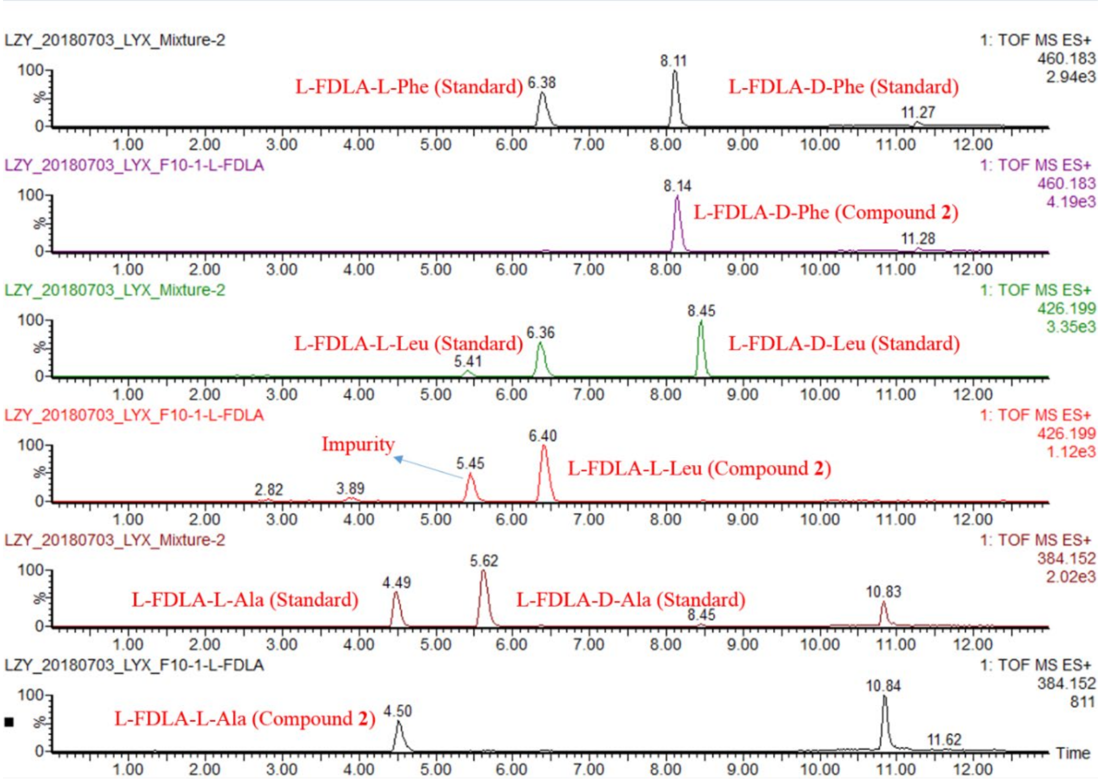

Figure S22. Mass chromatograms of the L-FDLA derivatives of standard amino acids and amino acids from acremonpeptide E (**2**)

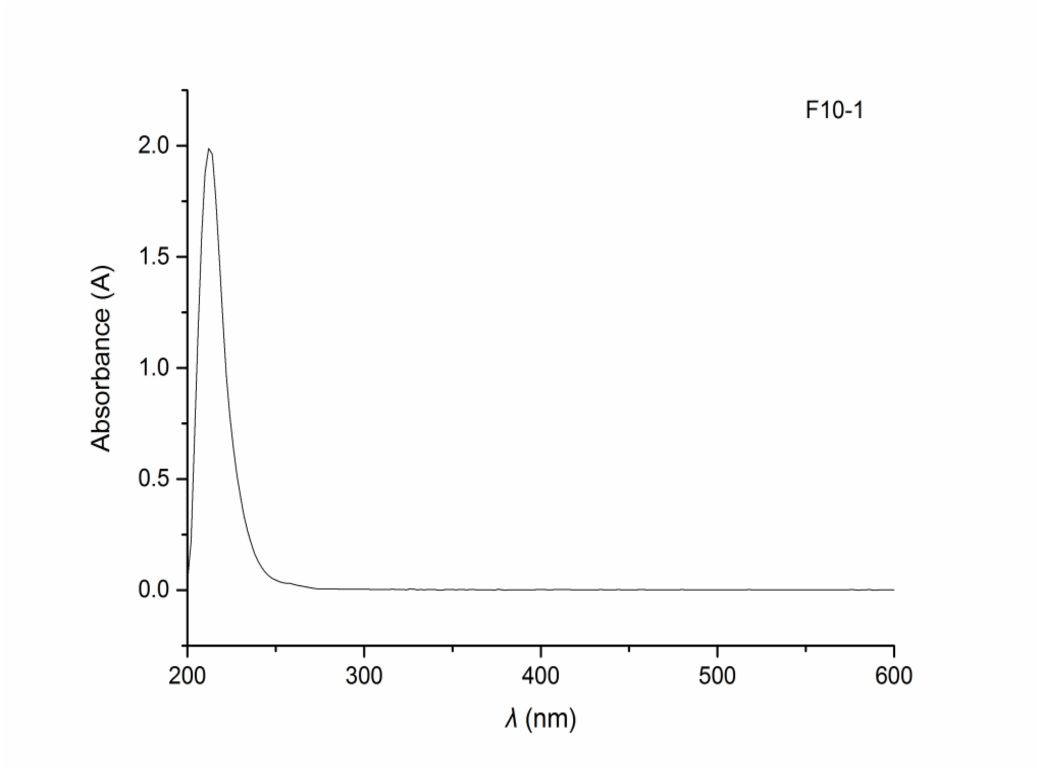

Figure S23. UV spectrum of acremonpeptide E (**2**) in MeOH.

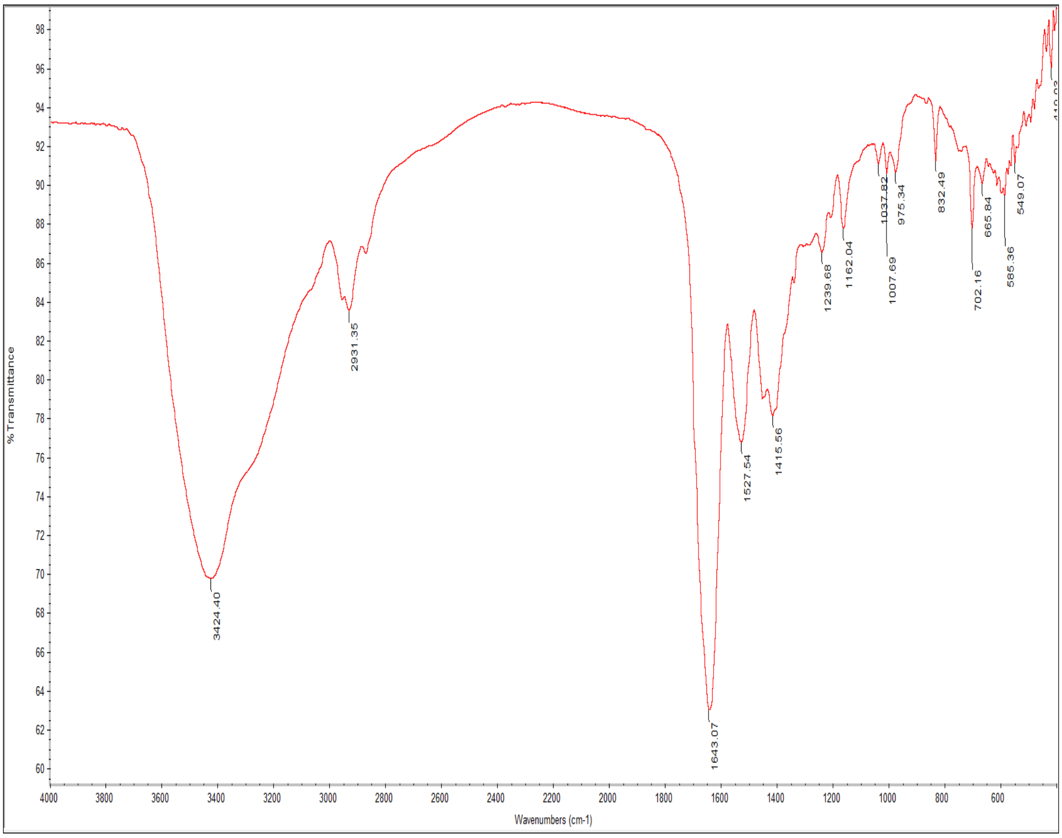

Figure S24. IR spectrum of acremonpeptide E (**2**).

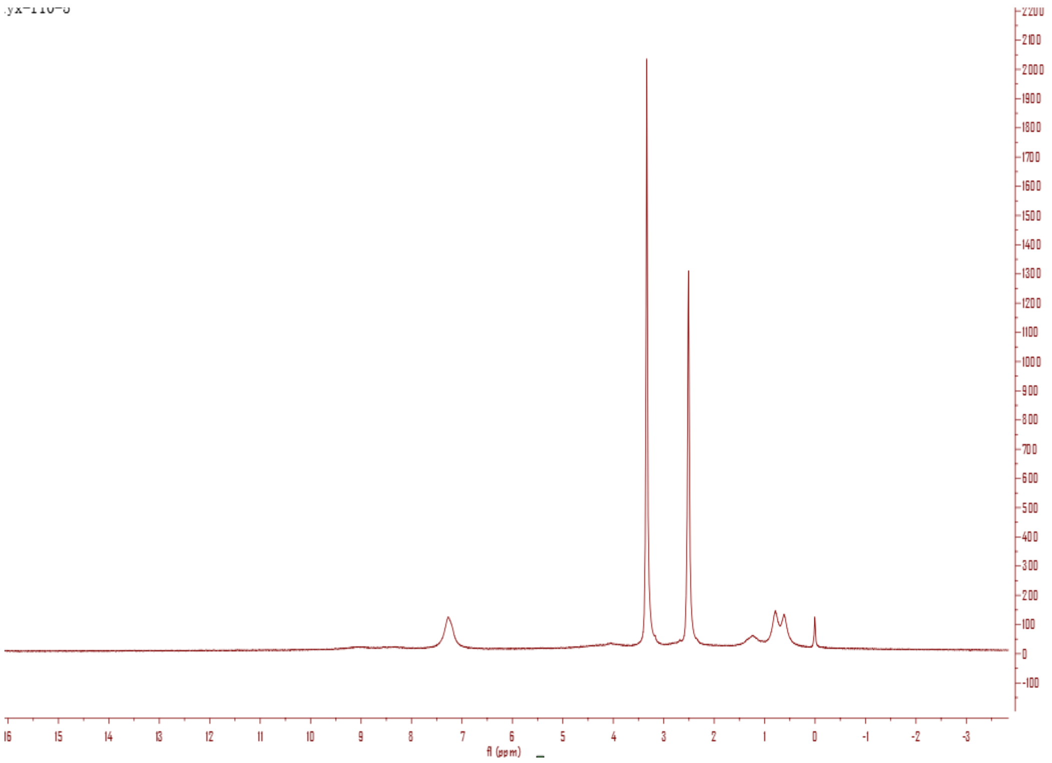

Figure S25. ^{1}H spectrum of Fe (III)-acremonpeptide E (**3**) in DMSO-d_6 (600 MHz).

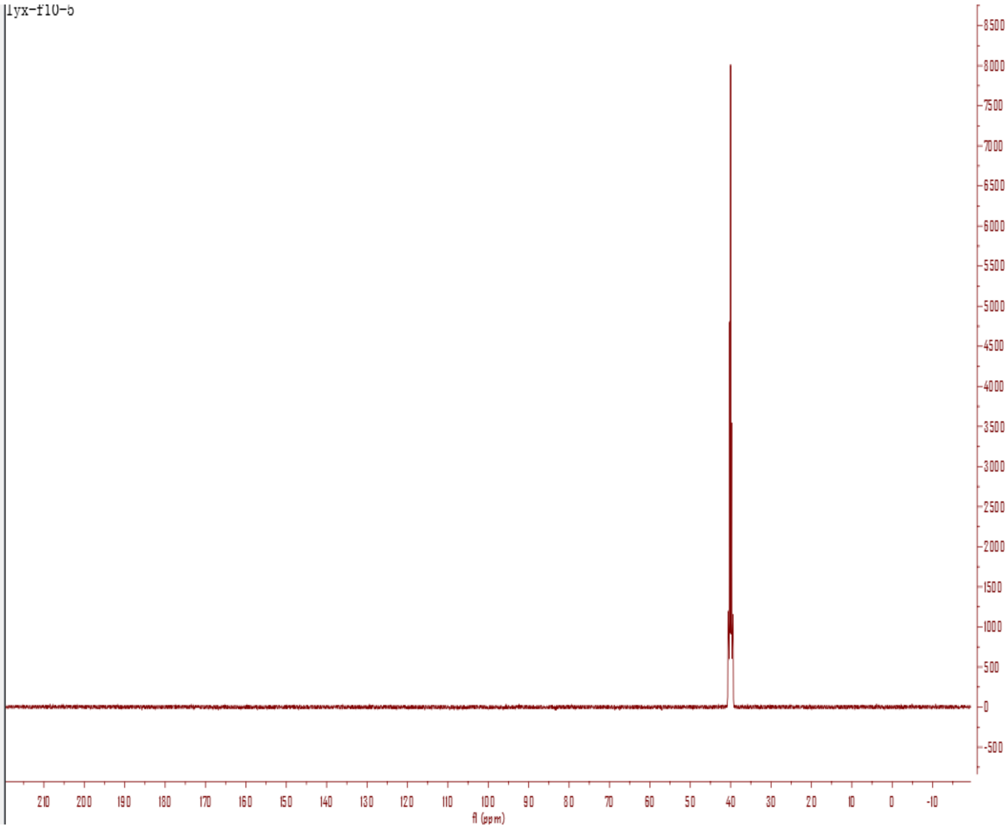

Figure S26. ^{13}C spectrum of Fe (III)-acremonpeptide E (**3**) in DMSO-d_6 (150 MHz).

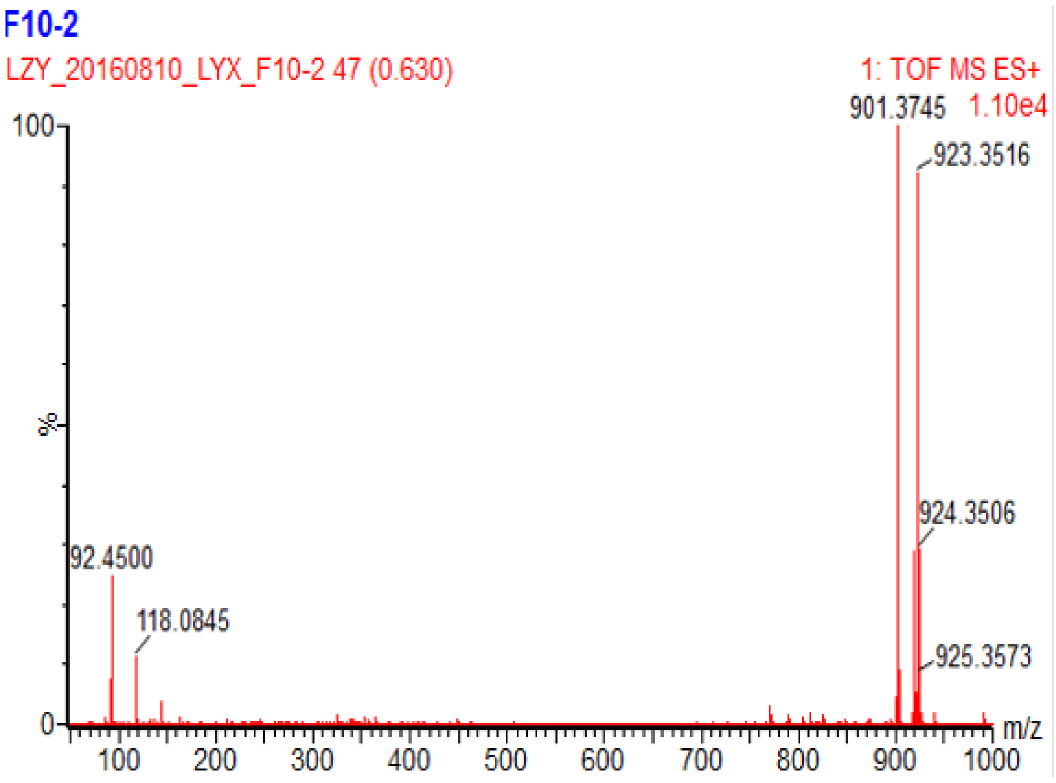

Figure S27. HRESIMS data of Fe (III)-acremonpeptide E (**3**).

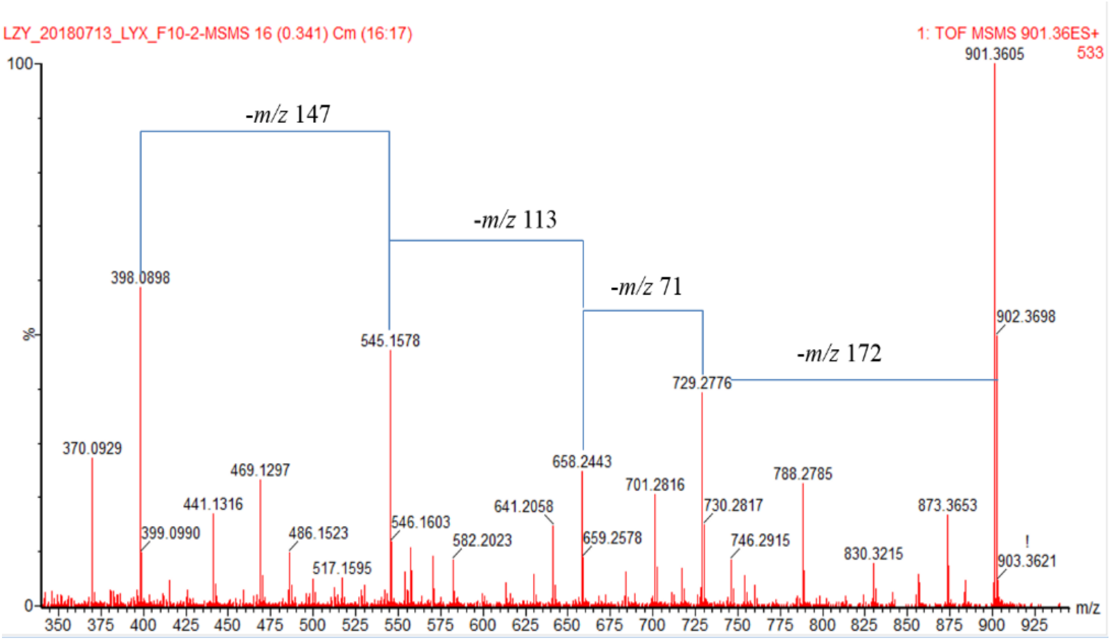

Figure S28. HRESIMS/MS fragmentation ions of Fe (III)-acremonpeptide E (**3**).

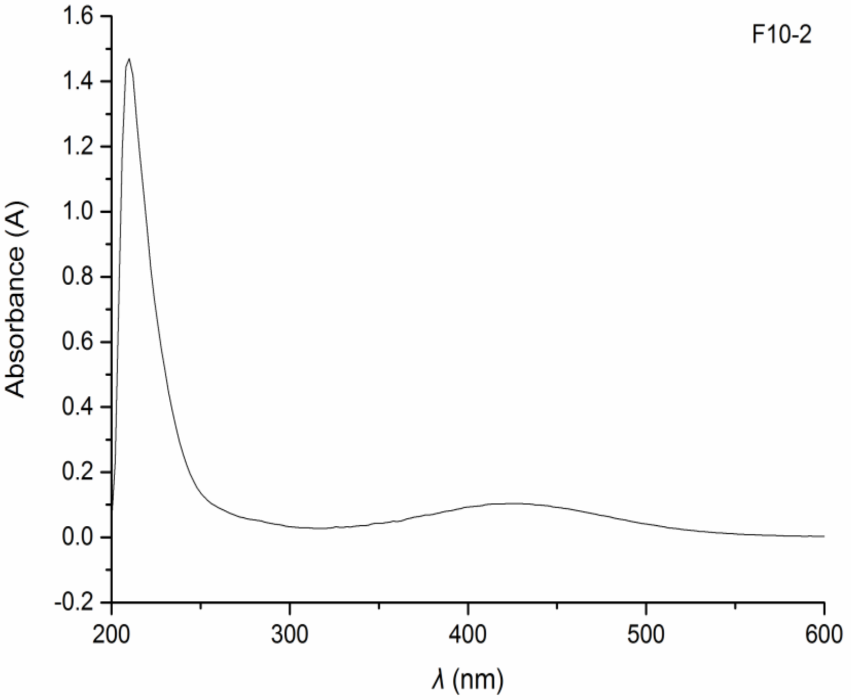

Figure S29. UV spectrum of Fe (III)-acremonpeptide E (**3**) in MeOH.

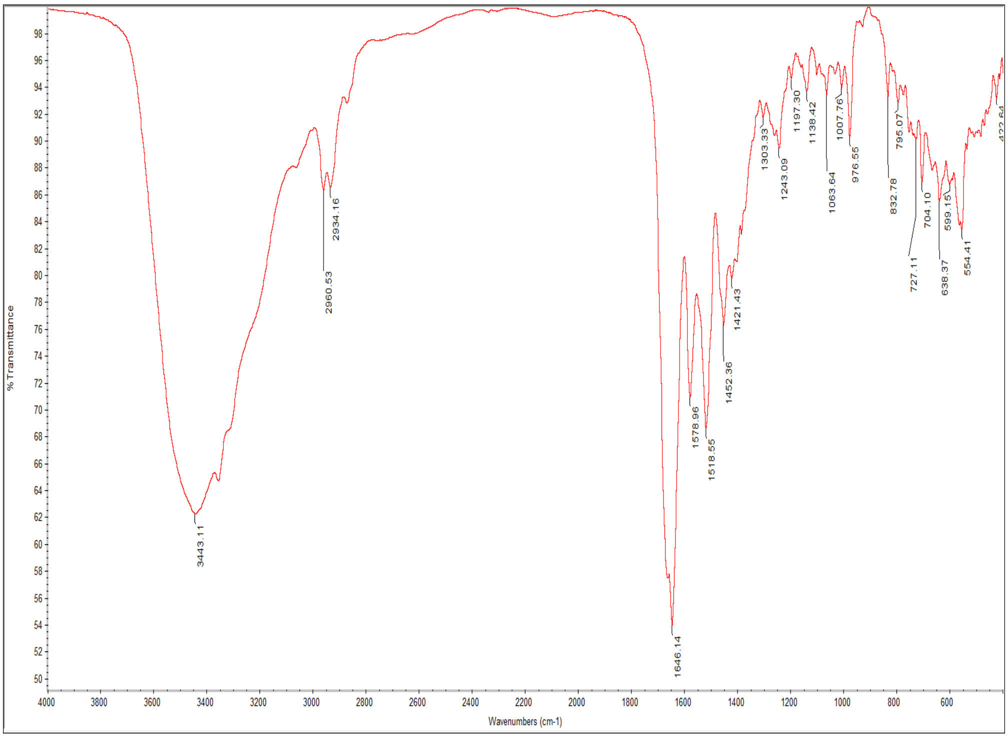

Figure S30. IR spectrum of Fe (III)-acremonpeptide E (**3**).

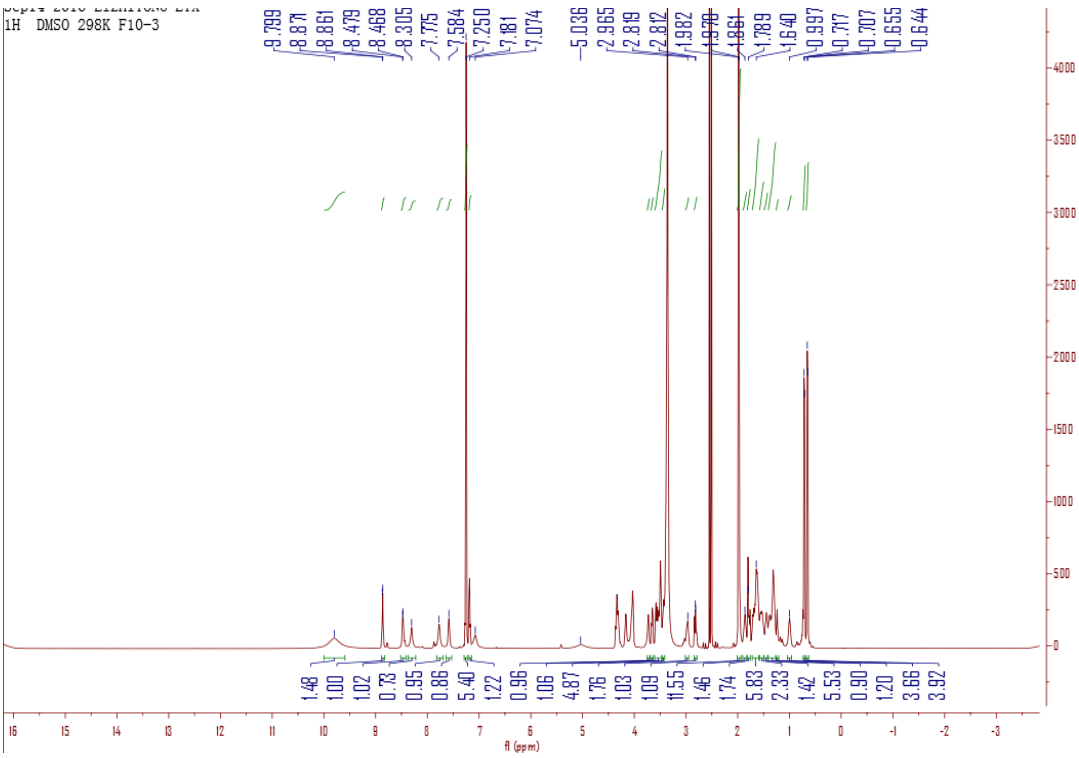

Figure S31. ^{1}H spectrum of acremonpeptide F (**4**) in DMSO-d_6 (600 MHz).

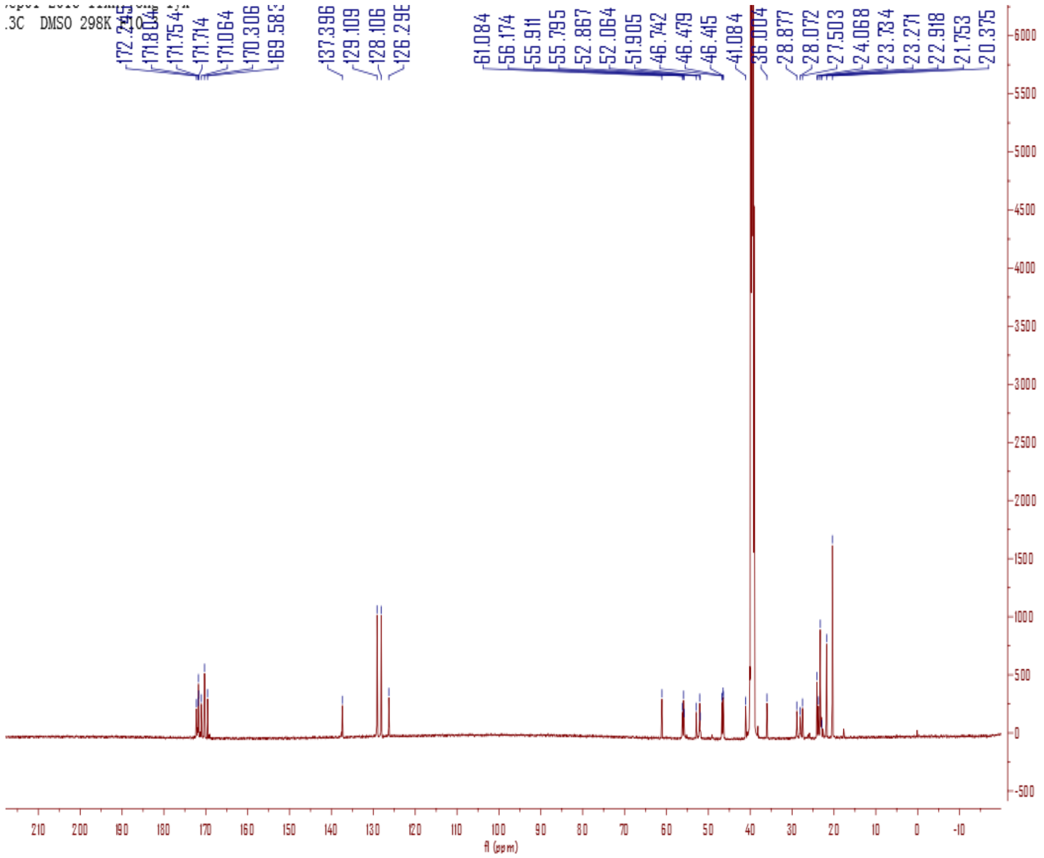

Figure S32. ^{13}C spectrum of acremonpeptide F (**4**) in DMSO-d_6 (150 MHz).

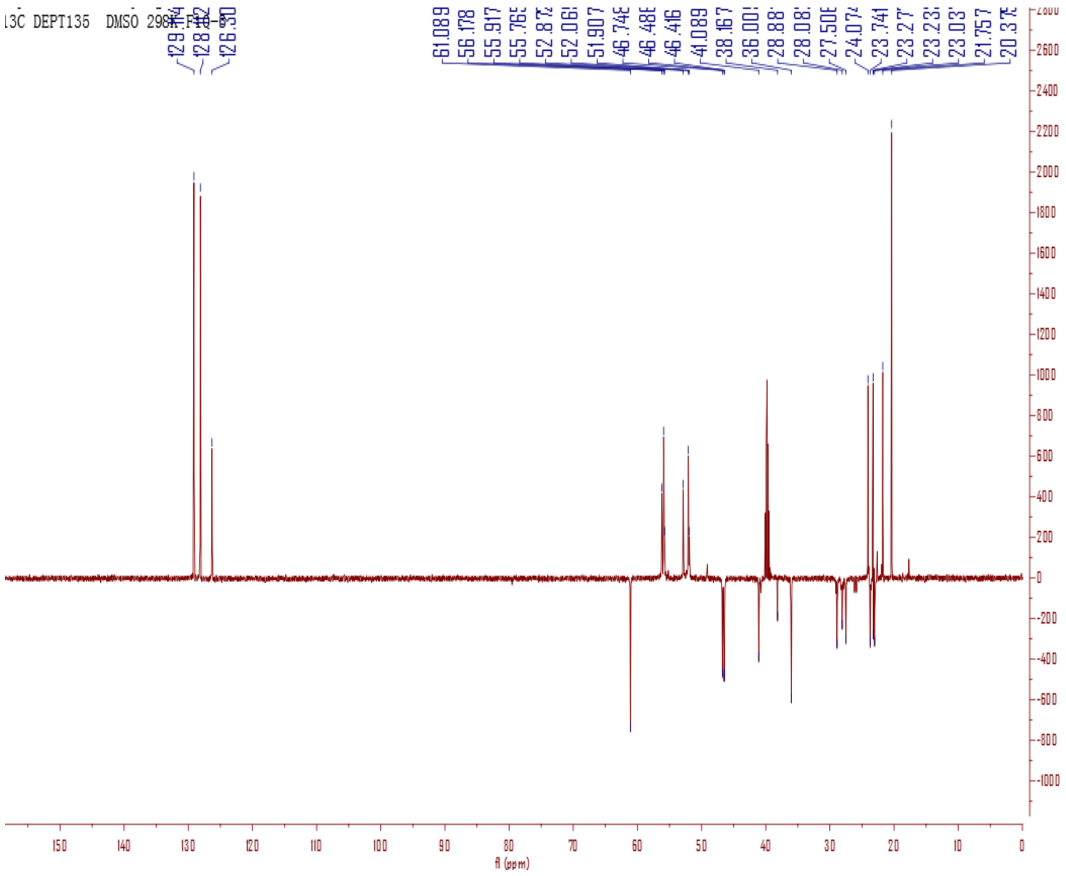

Figure S33. DEPT135 spectrum of acremonpeptide F (**4**) in DMSO-d_6 (150 MHz).

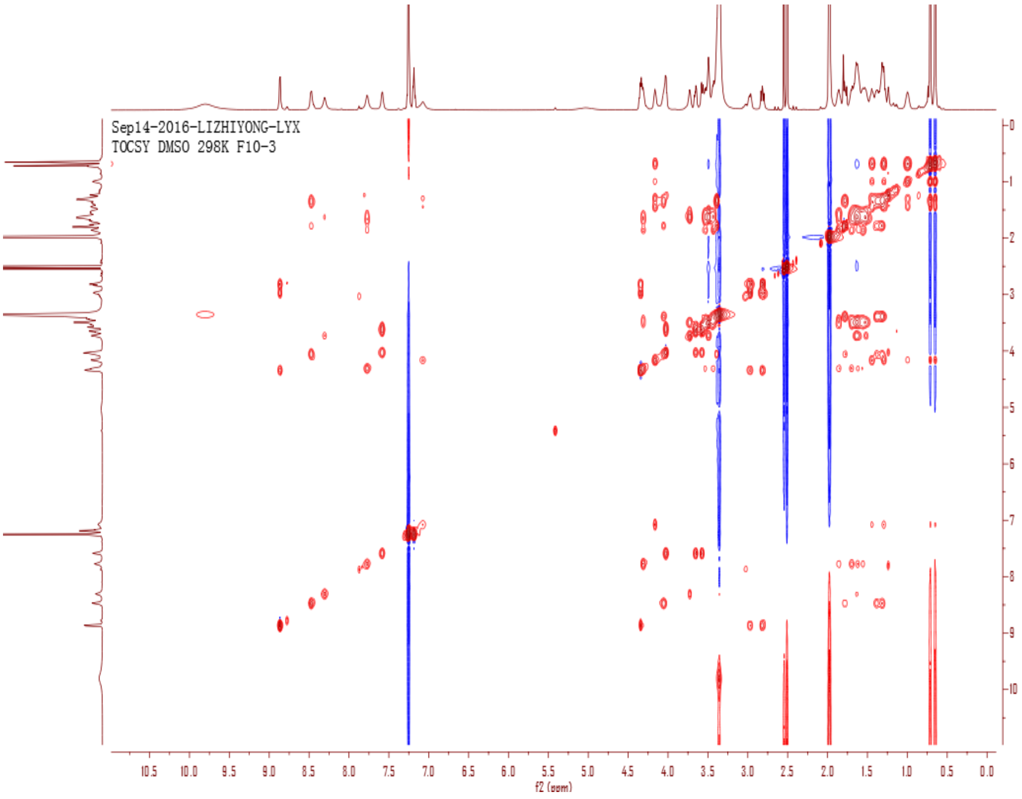

Figure S34. TOCSY spectrum of acremonpeptide F (**4**) in DMSO-d_6.

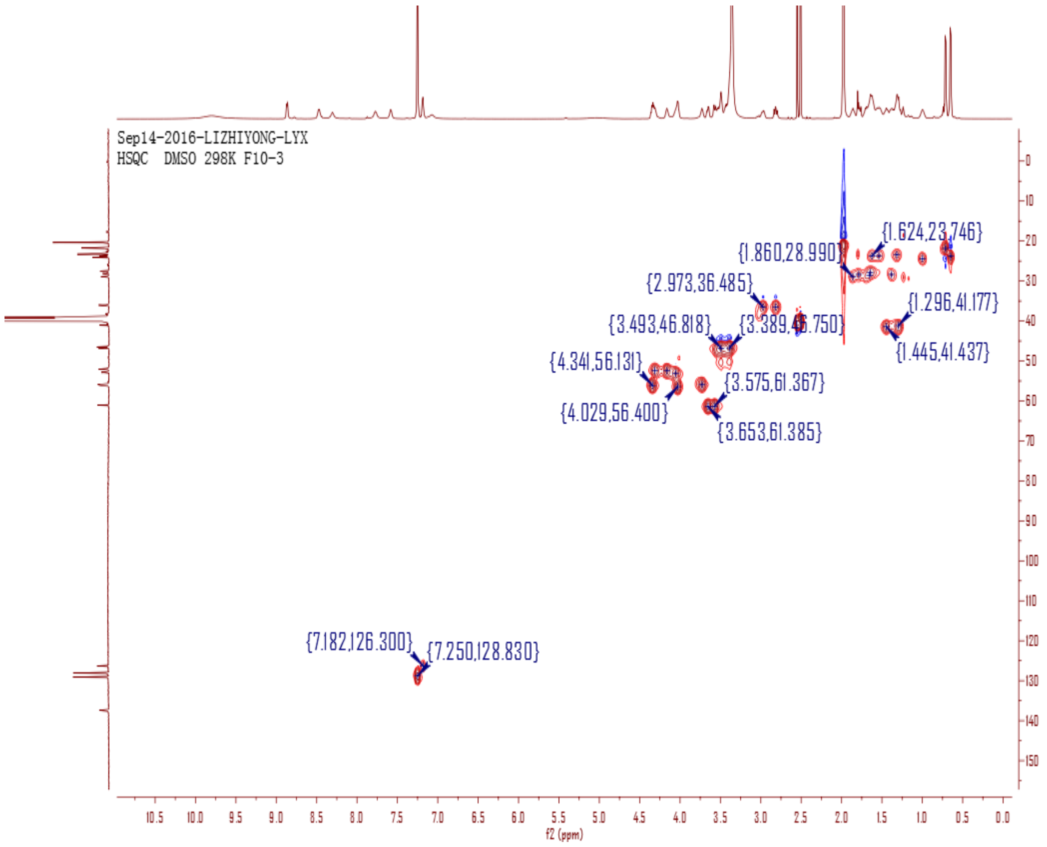

Figure S35. HSQC spectrum of acremonpeptide F (**4**) in DMSO-d_6.

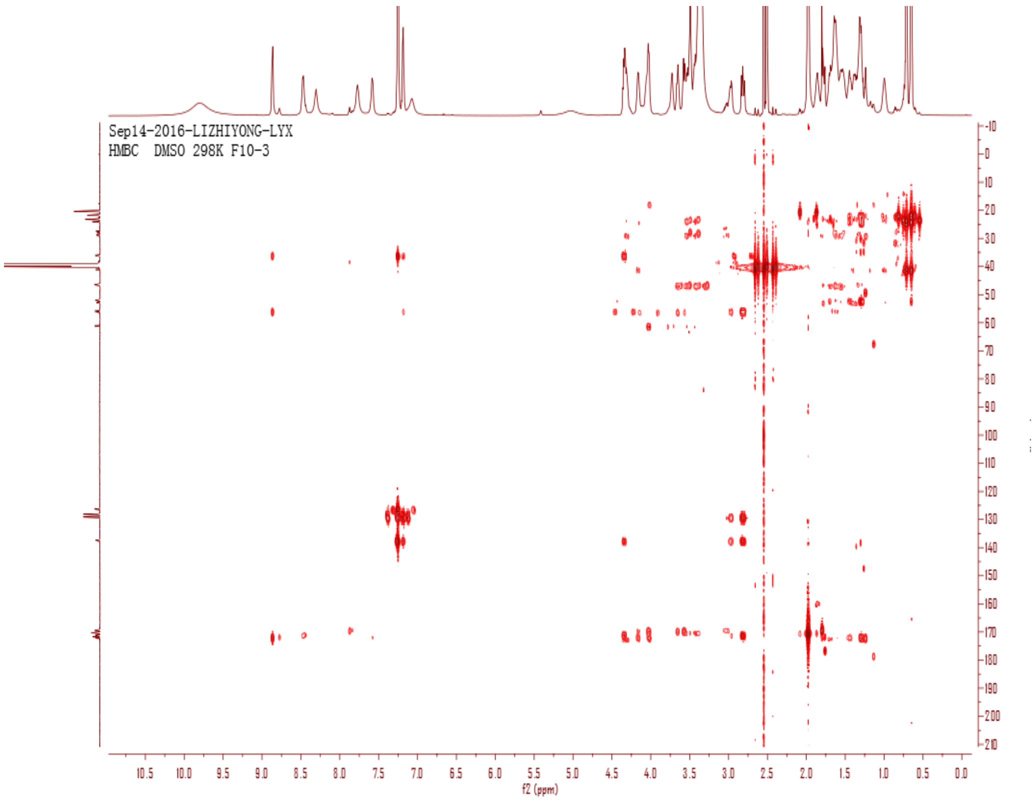

Figure S36. HMBC spectrum of acremonpeptide F (**4**) in DMSO-d_6.

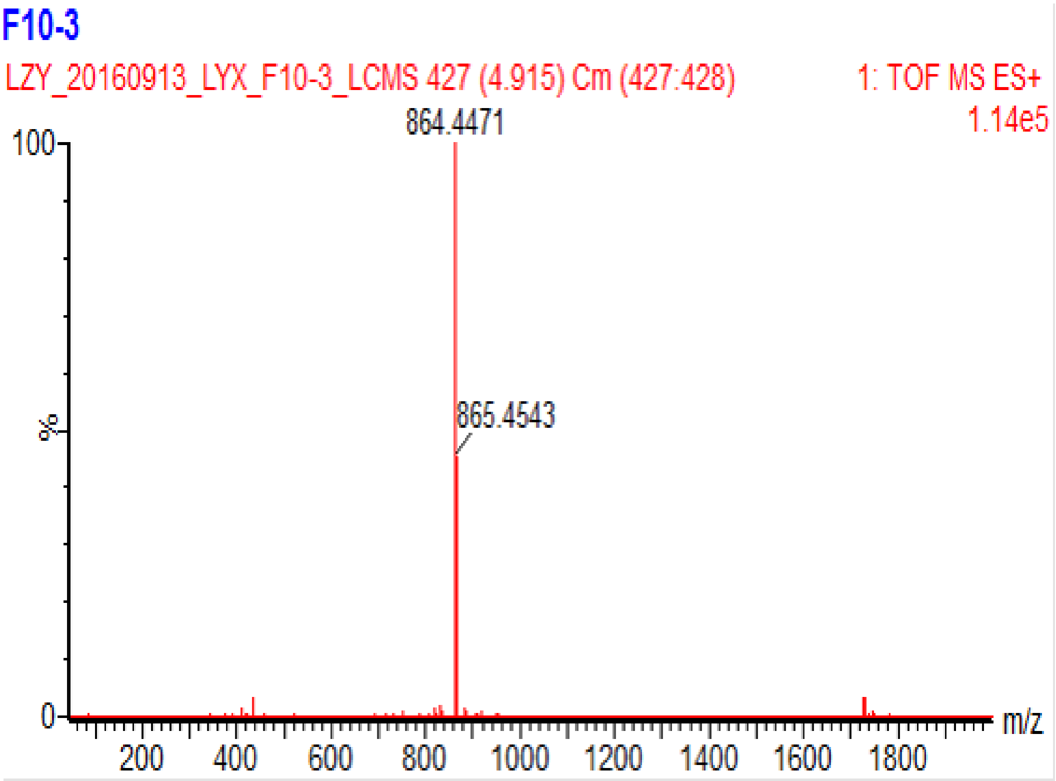

Figure S37. HRESIMS data of acremonpeptide F (**4**).

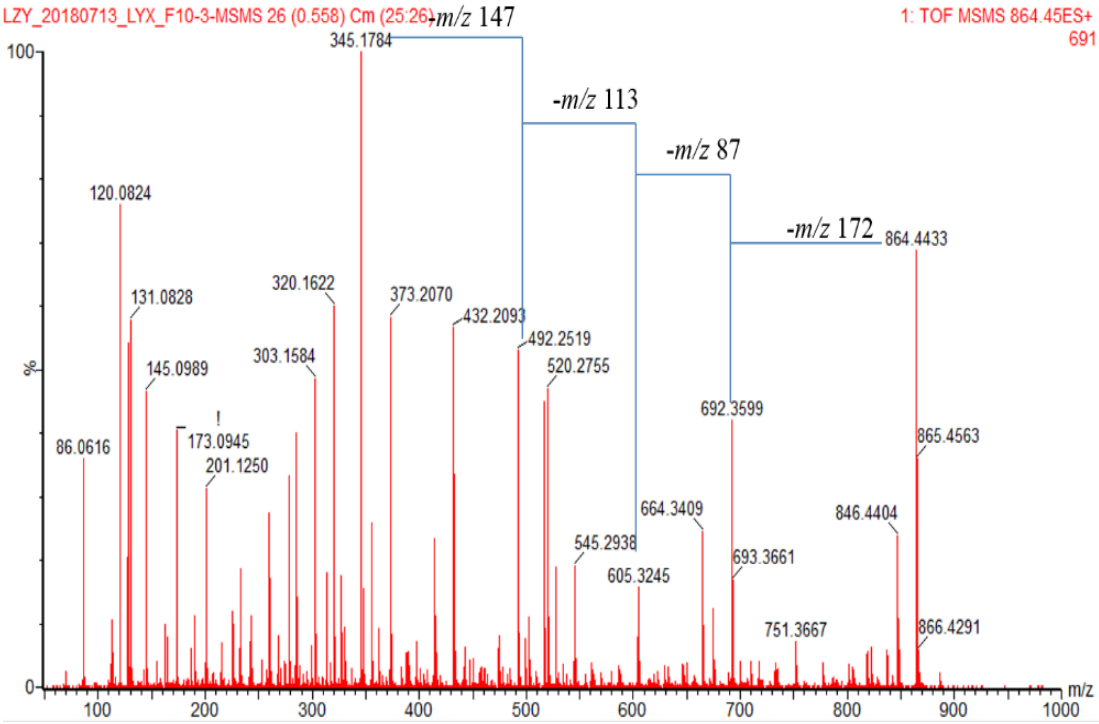

Figure S38. HRESIMS/MS fragmentation ions of acremonpeptide F (**4**).

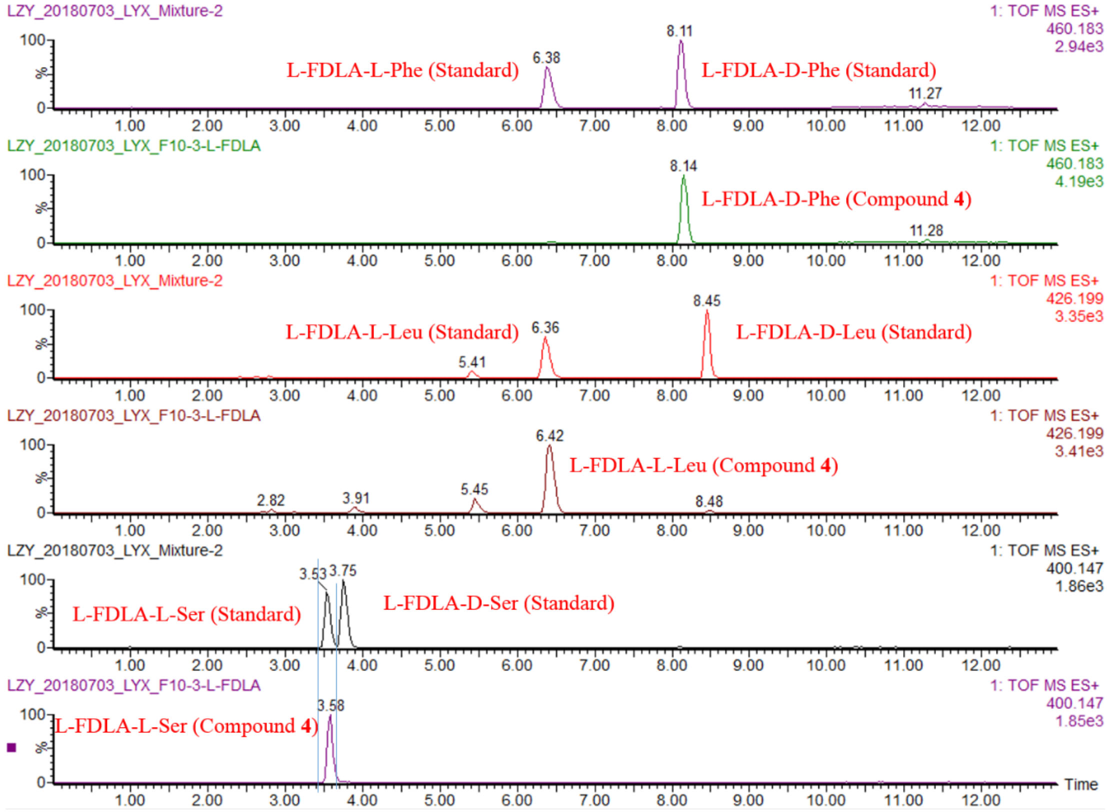

Figure S39. Mass chromatograms of the L-FDLA derivatives of standard amino acids and amino acids from acremonpeptide F (**4**).

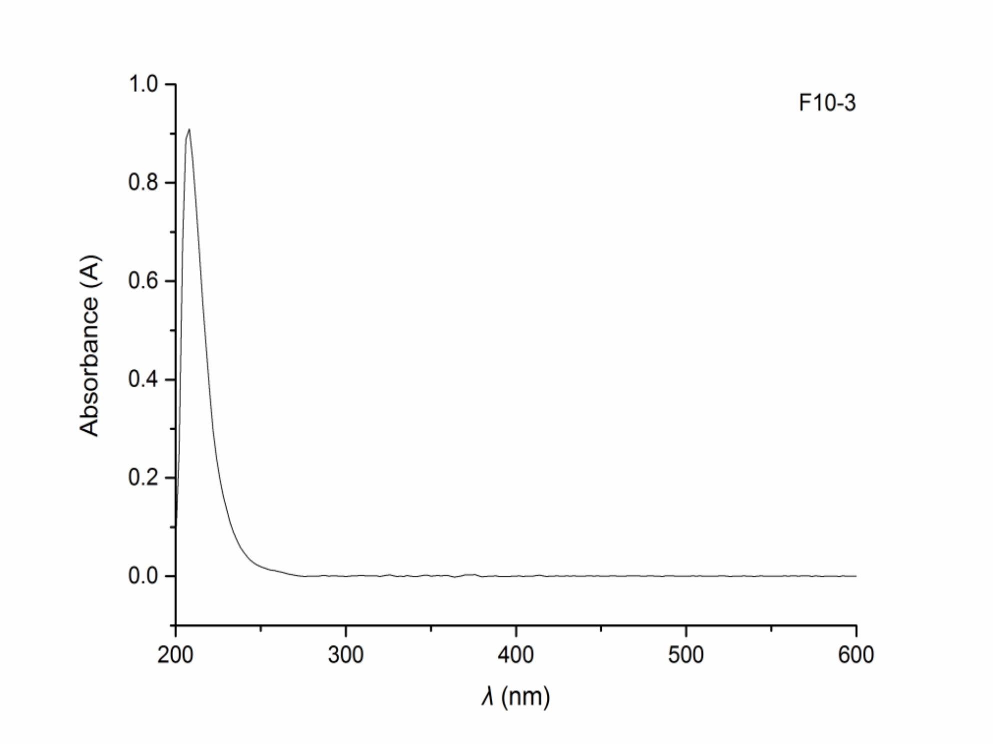

Figure S40. UV spectrum of acremonpeptide F (**4**) in MeOH.

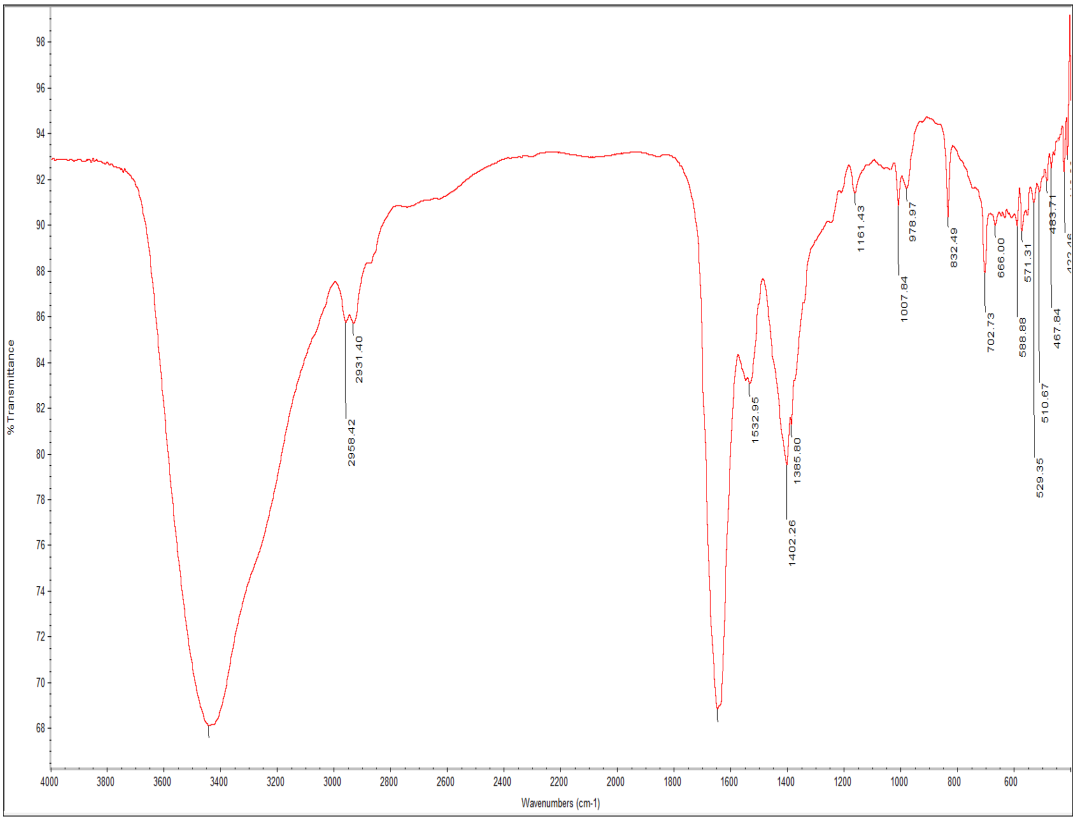

Figure S41. IR spectrum of acremonpeptide F (**4**).

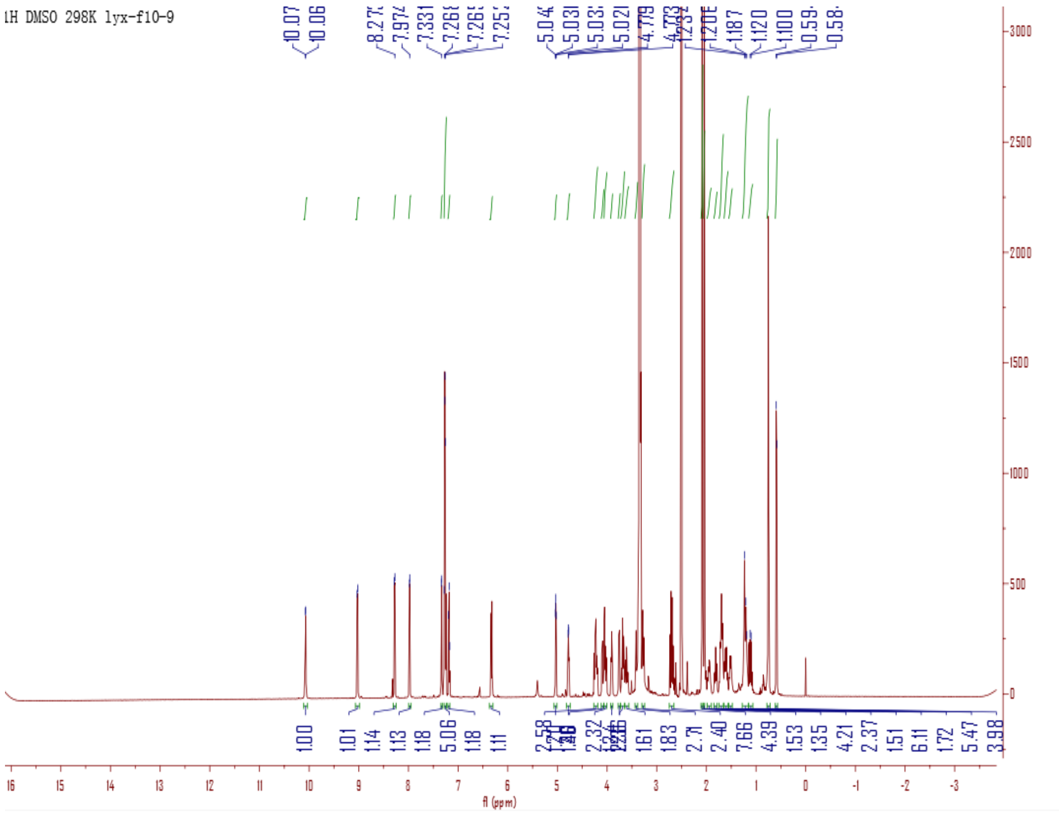

Figure S42. ^{1}H spectrum of Al (Ⅲ)-acremonpeptide F (**5**) in DMSO-d_6 (600 MHz).

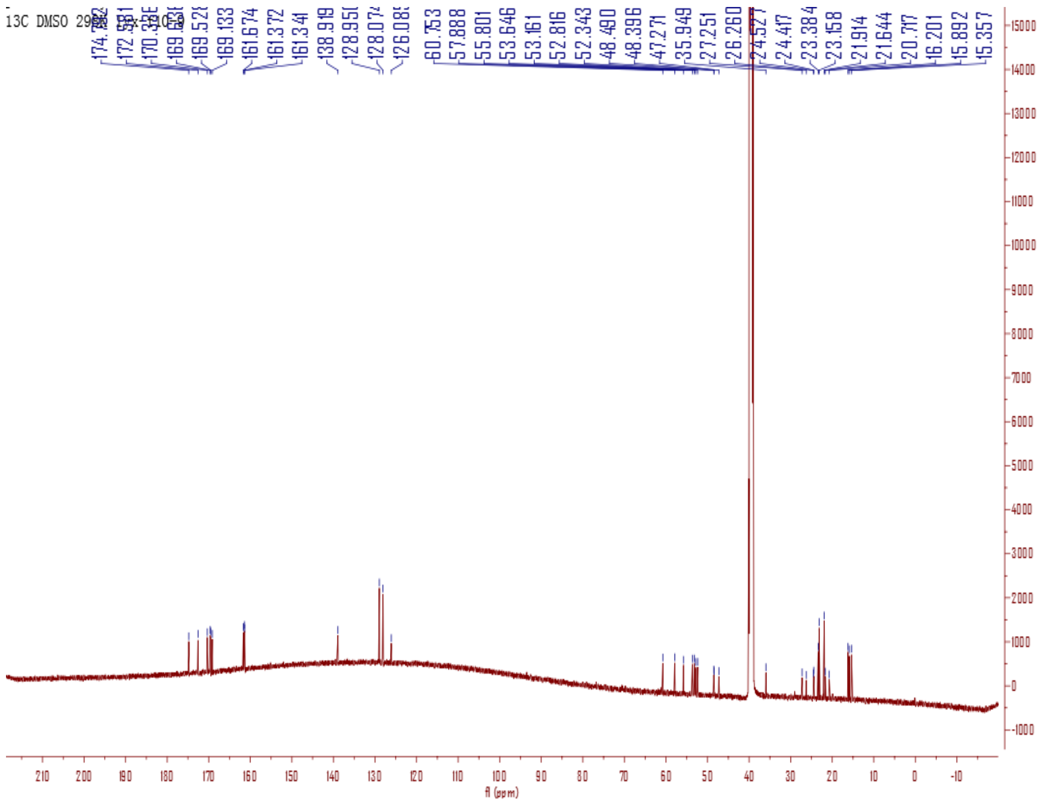

Figure S43. ^{13}C spectrum of Al (III)-acremonpeptide F (**5**) in DMSO-d_6 (150 MHz).

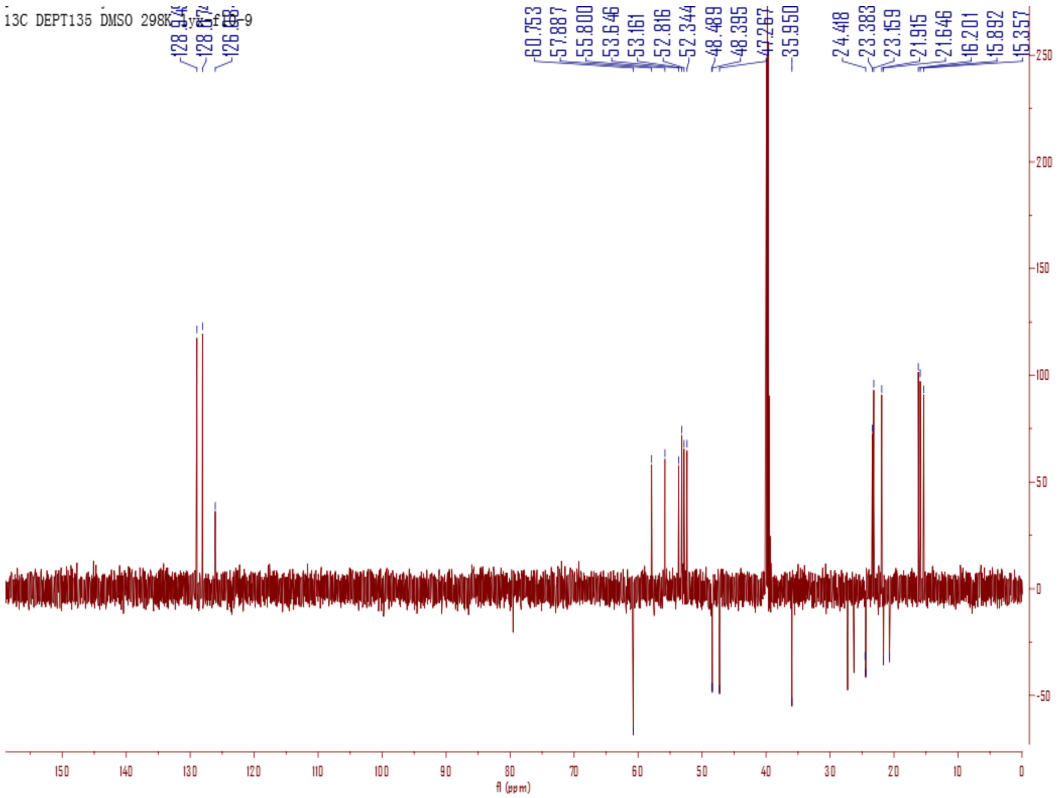

Figure S44. DEPT135 spectrum of Al (III)-acremonpeptide F (**5**) in DMSO-d_6 (150 MHz).

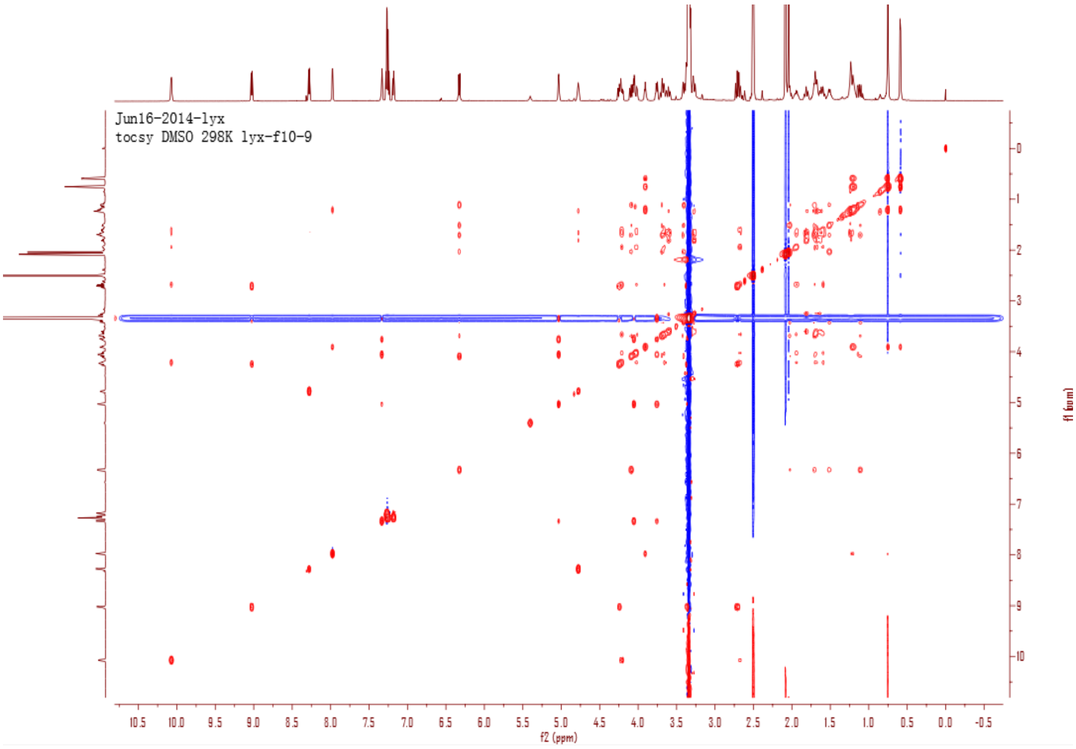

Figure S45. TOCSY spectrum of Al (III)-acremonpeptide F (**5**) in DMSO-d_6.

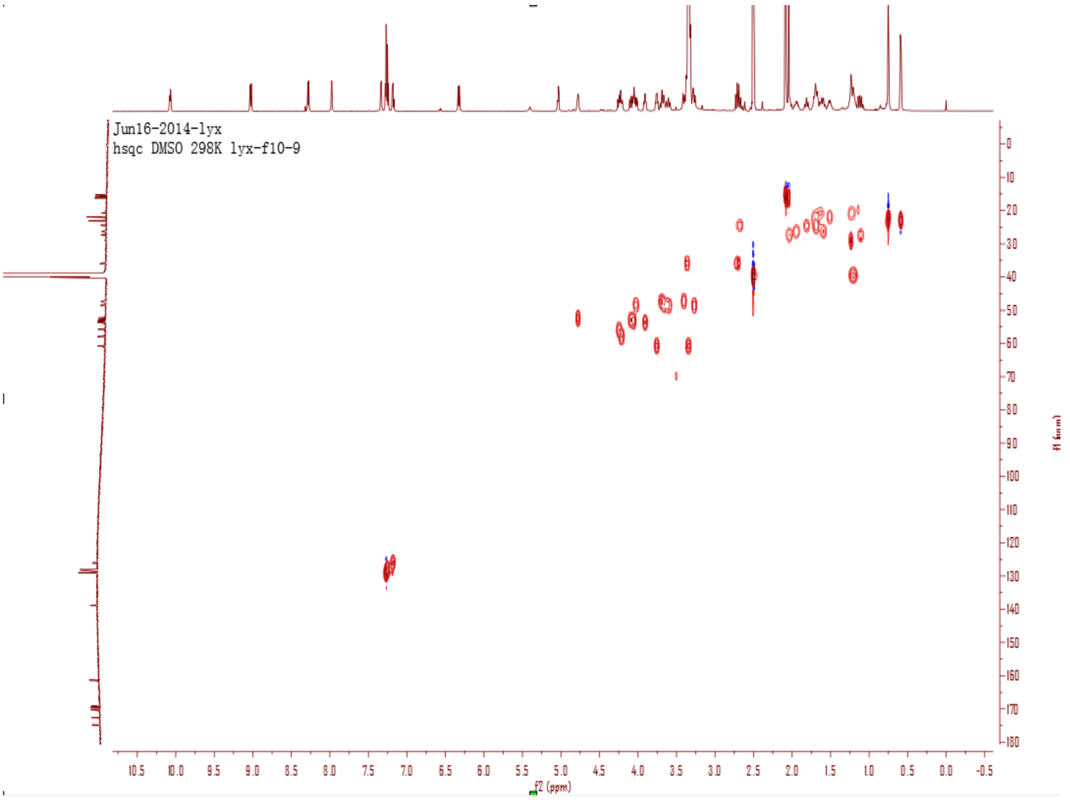

Figure S46. HSQC spectrum of Al (III)-acremonpeptide F (**5**) in DMSO-d_6.

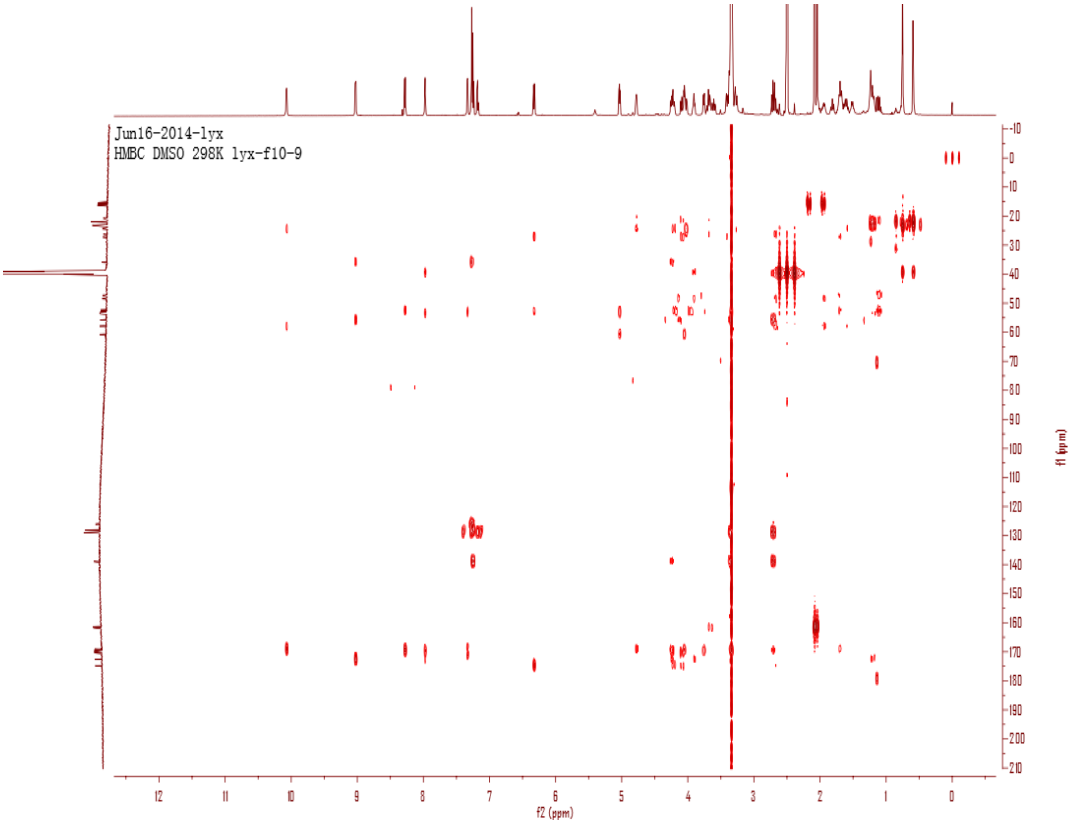

Figure S47. HMBC spectrum of Al (Ⅲ)-acremonpeptide F (**5**) in DMSO-d_6.

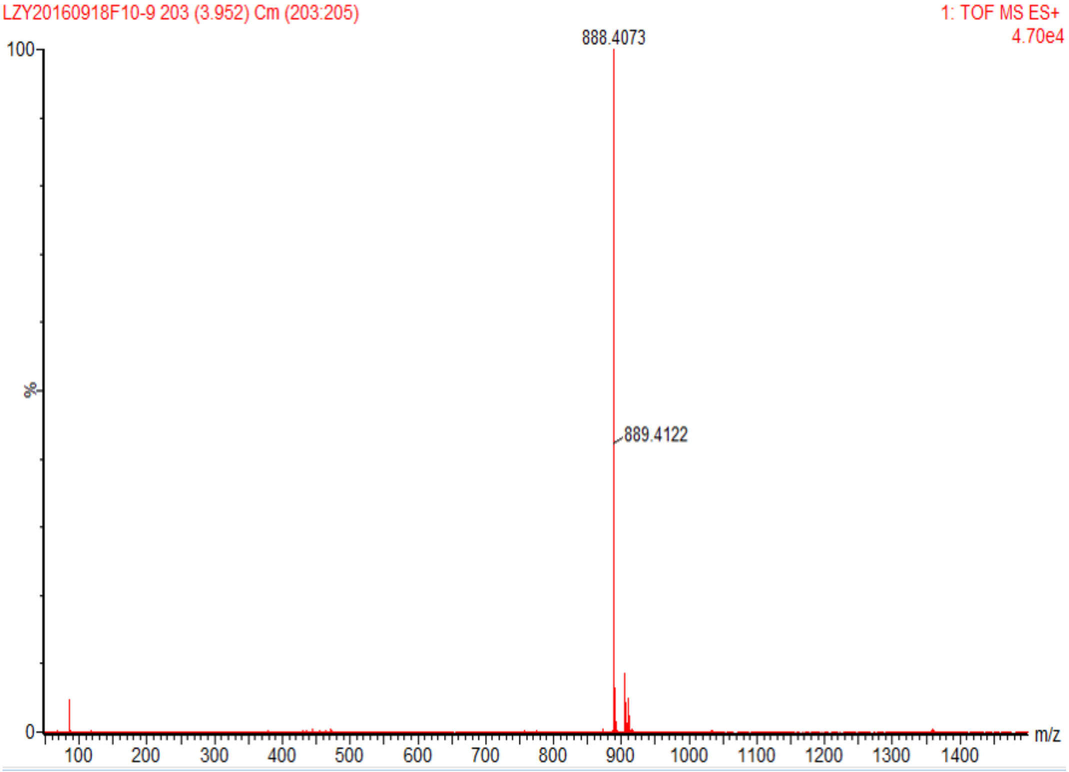

Figure S48. HRESIMS data of Al (Ⅲ)-acremonpeptide F (**5**).

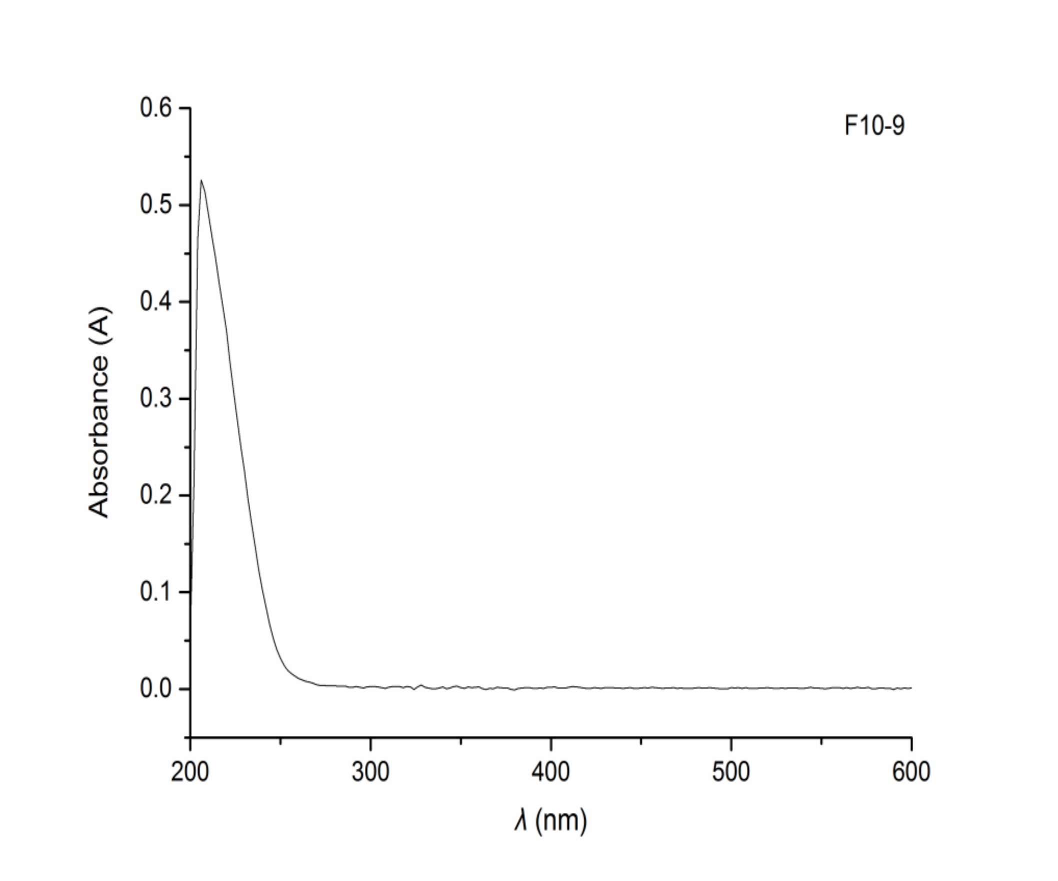

Figure S49. UV spectrum of Al (Ⅲ)-acremonpeptide F (**5**) in MeOH.

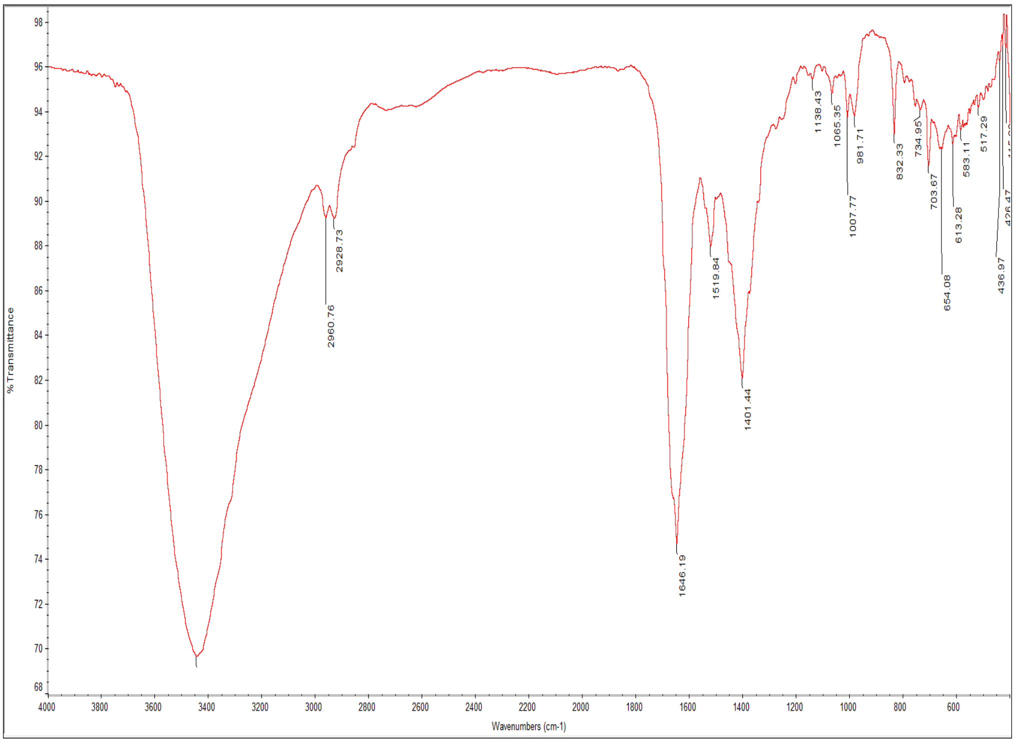

Figure S50. IR spectrum of Al (Ⅲ)-acremonpeptide F (**5**).

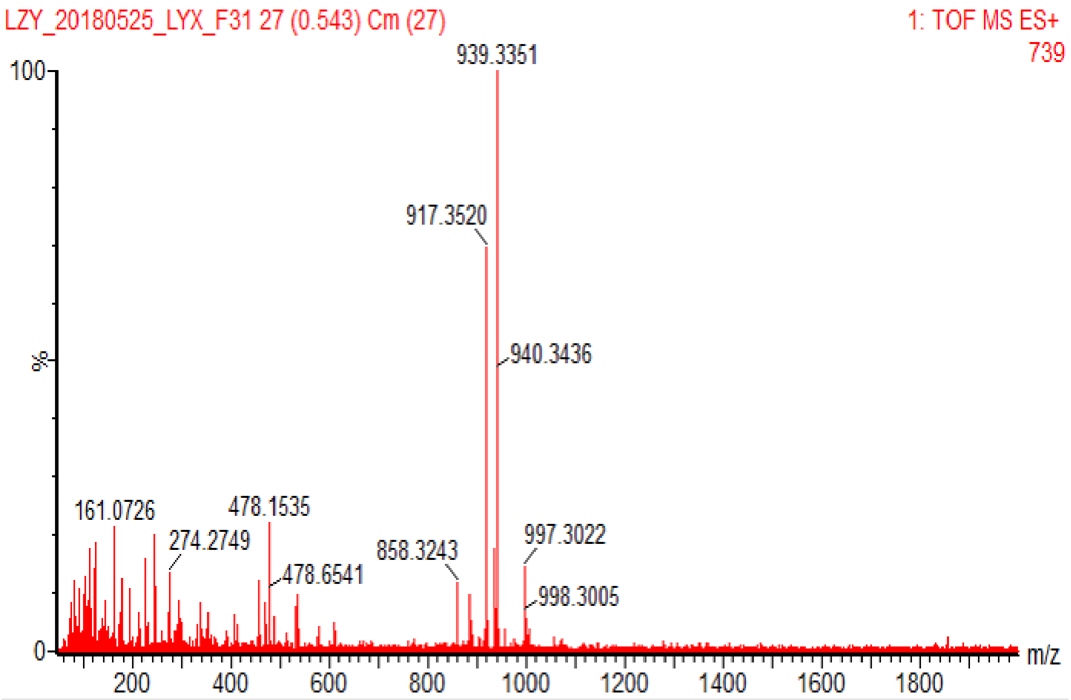

Figure S51. HRESIMS data of Fe (Ⅲ)-acremonpeptide F (**6**).

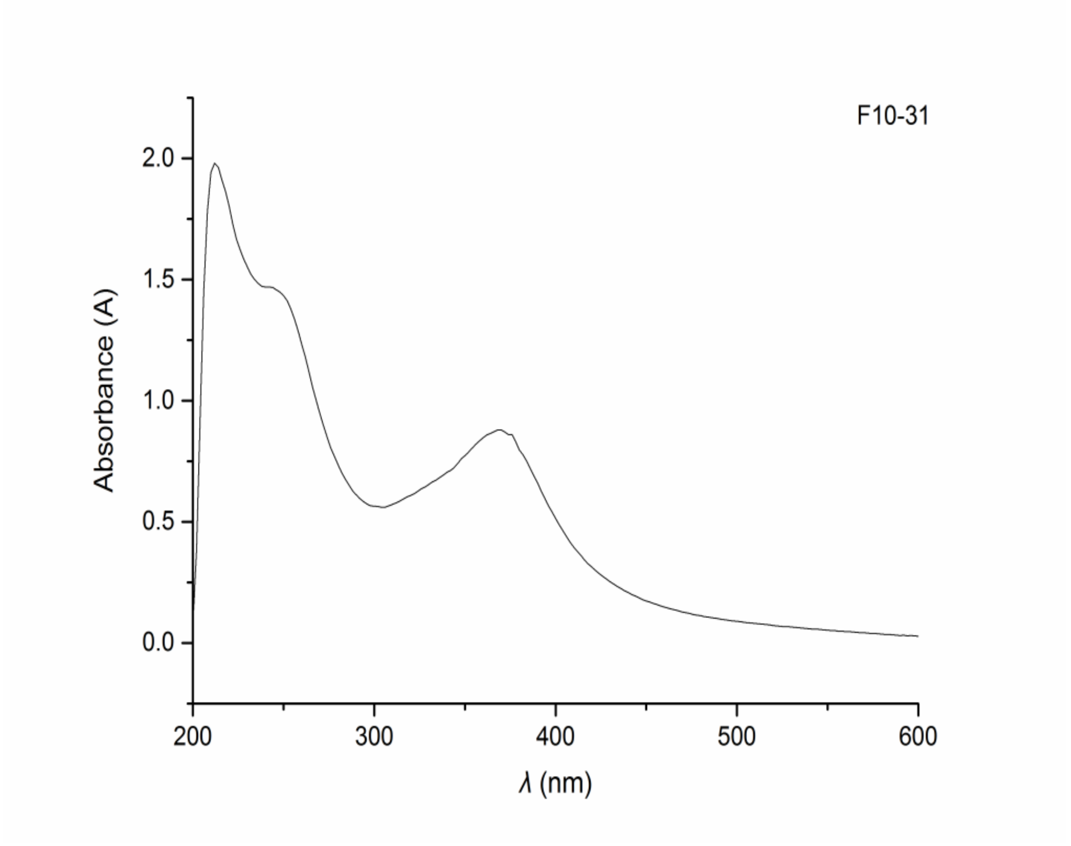

Figure S52. UV spectrum of Fe (Ⅲ)-acremonpeptide F (**6**) in MeOH.

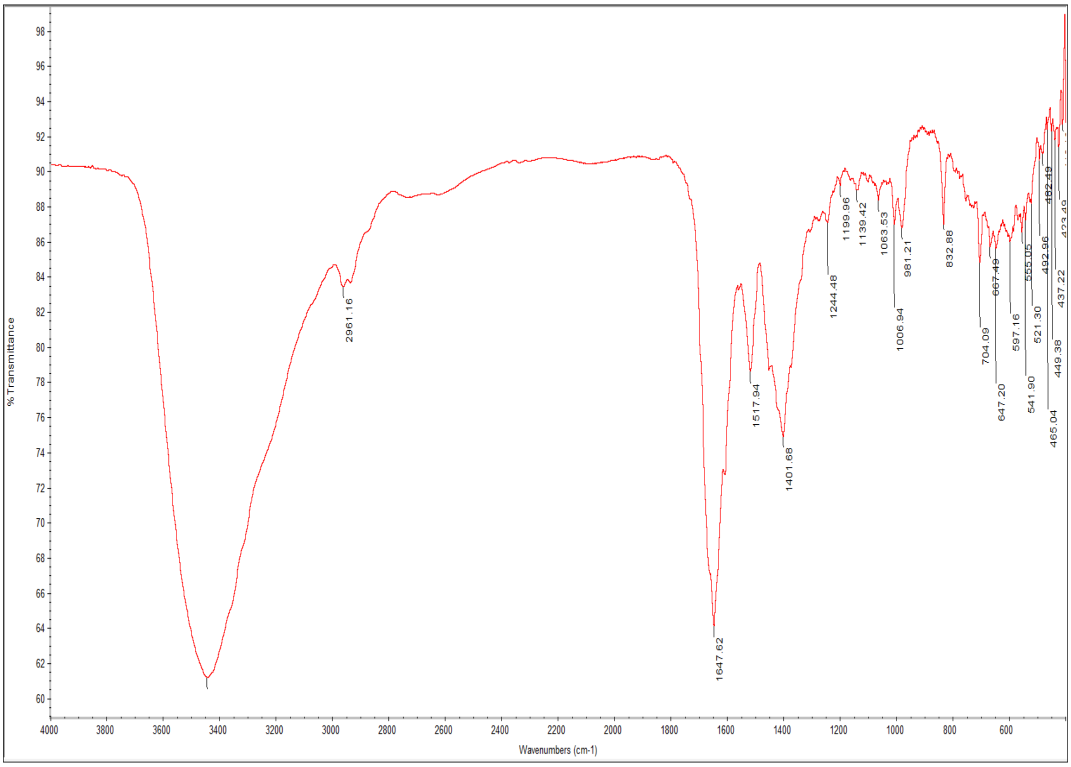

Figure S53. IR spectrum of Fe (III)-acremonpeptide F (**6**).

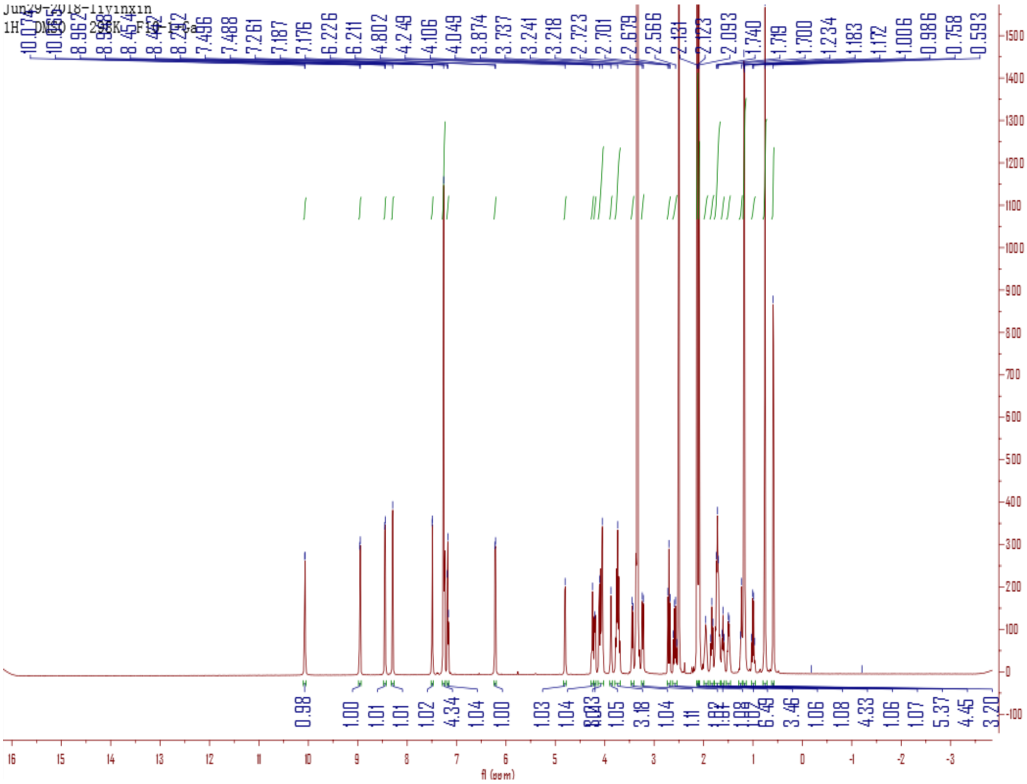

Figure S54. ^{1}H spectrum of Ga (III)-acremonpeptide E (**7**) in DMSO-d_6 (600 MHz).

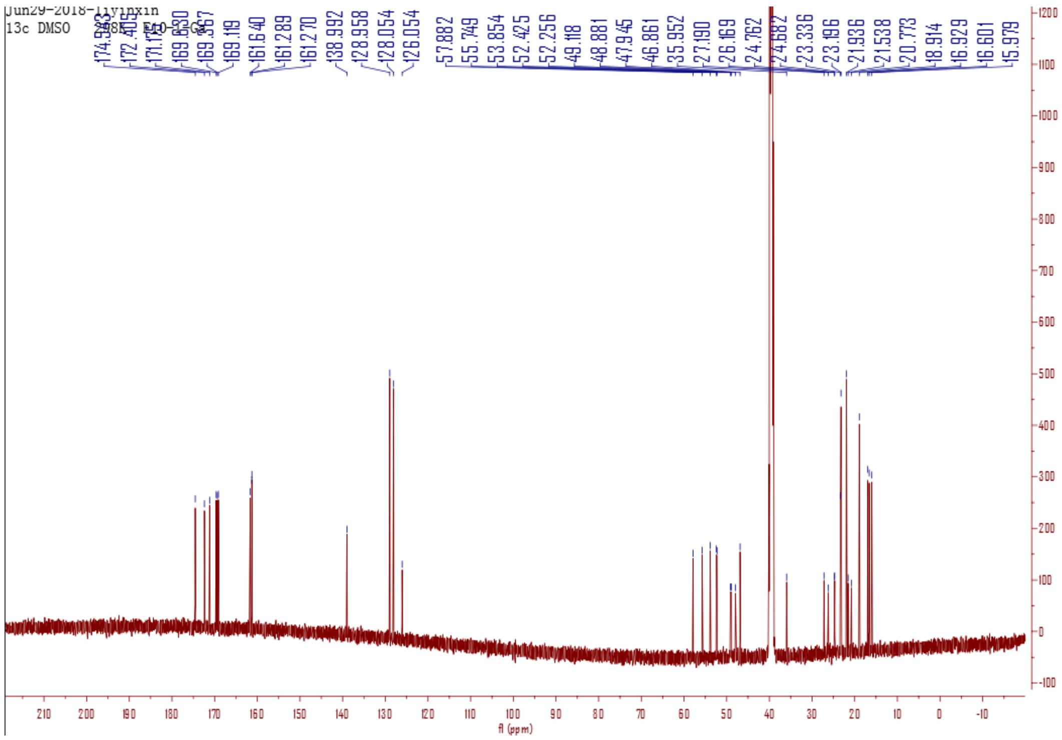

Figure S55. ^{13}C spectrum of Ga (III)-acremonpeptide E (**7**) in DMSO-d_6 (150 MHz).

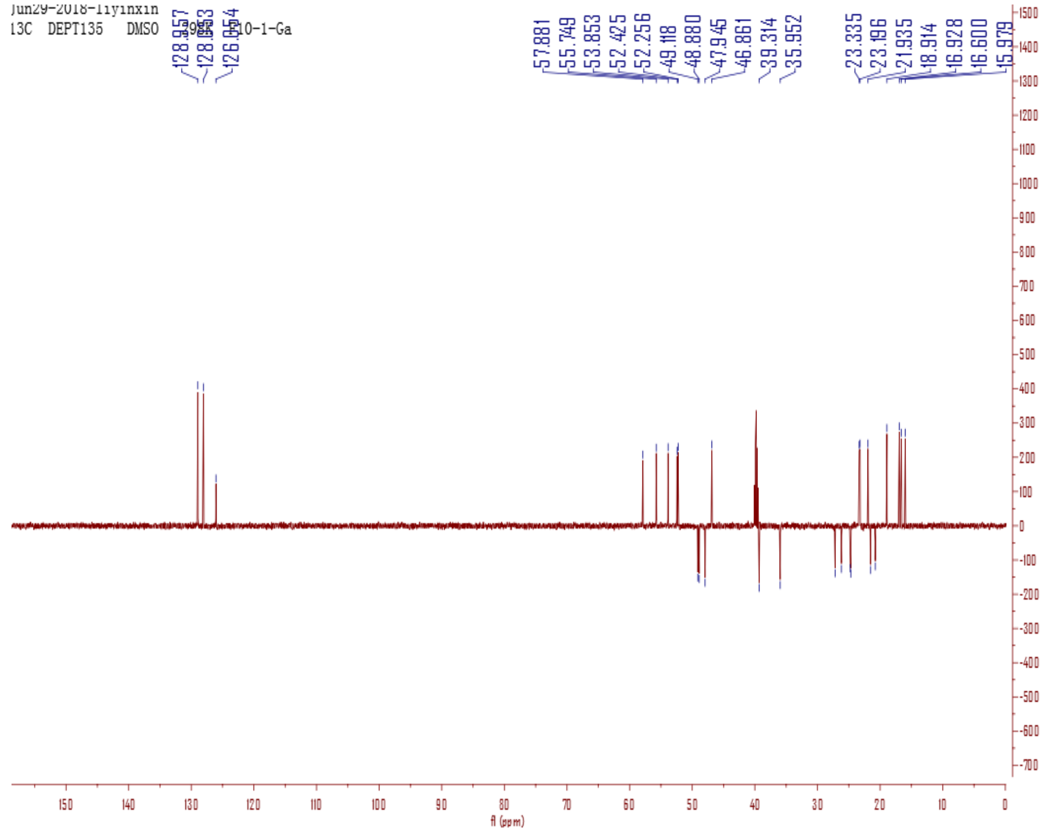

Figure S56. DEPT135 spectrum of Ga (III)-acremonpeptide E (**7**) in DMSO-d_6 (150 MHz).

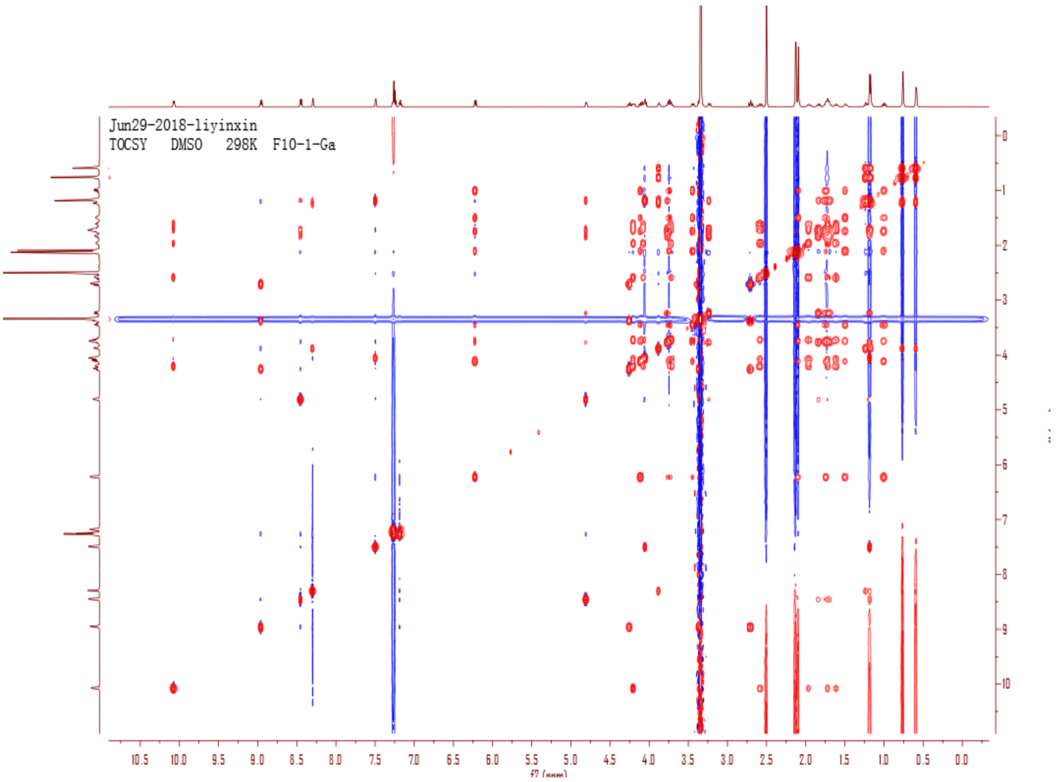

Figure S57. TOCSY spectrum of Ga (III)-acremonpeptide E (**7**) in DMSO-d_6

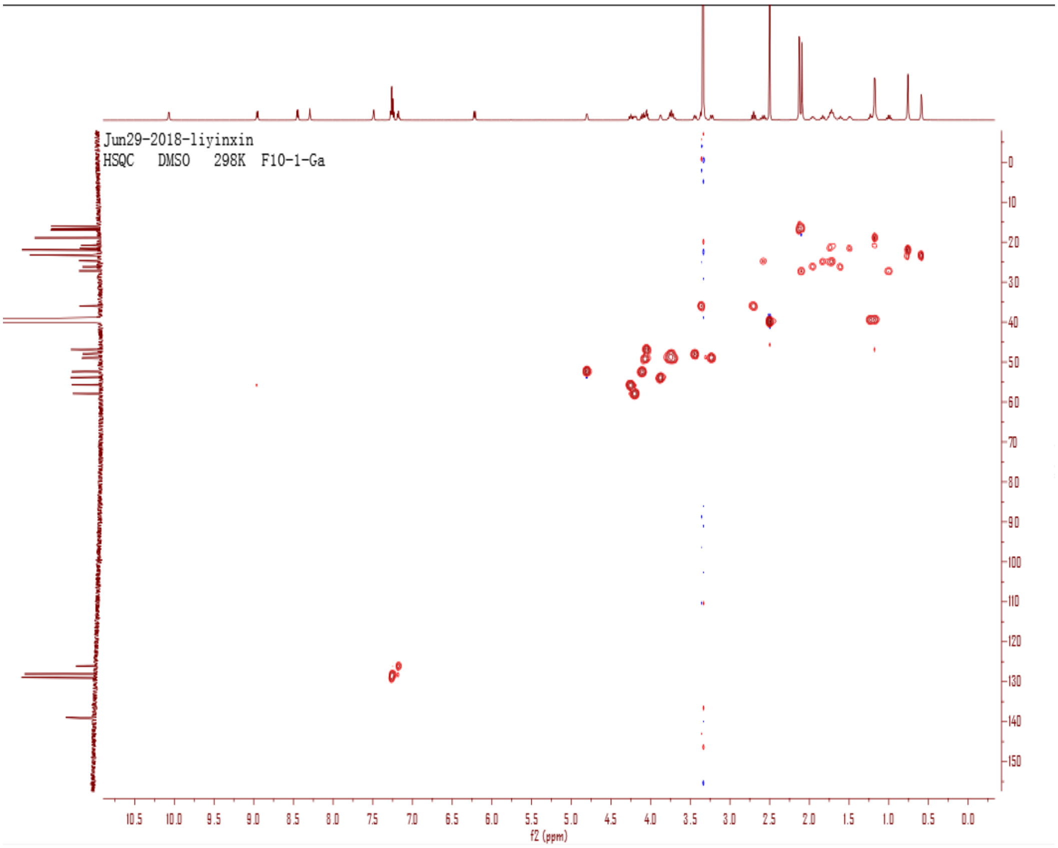

Figure S58. HSQC spectrum of Ga (III)-acremonpeptide E (**7**) in DMSO-d_6.

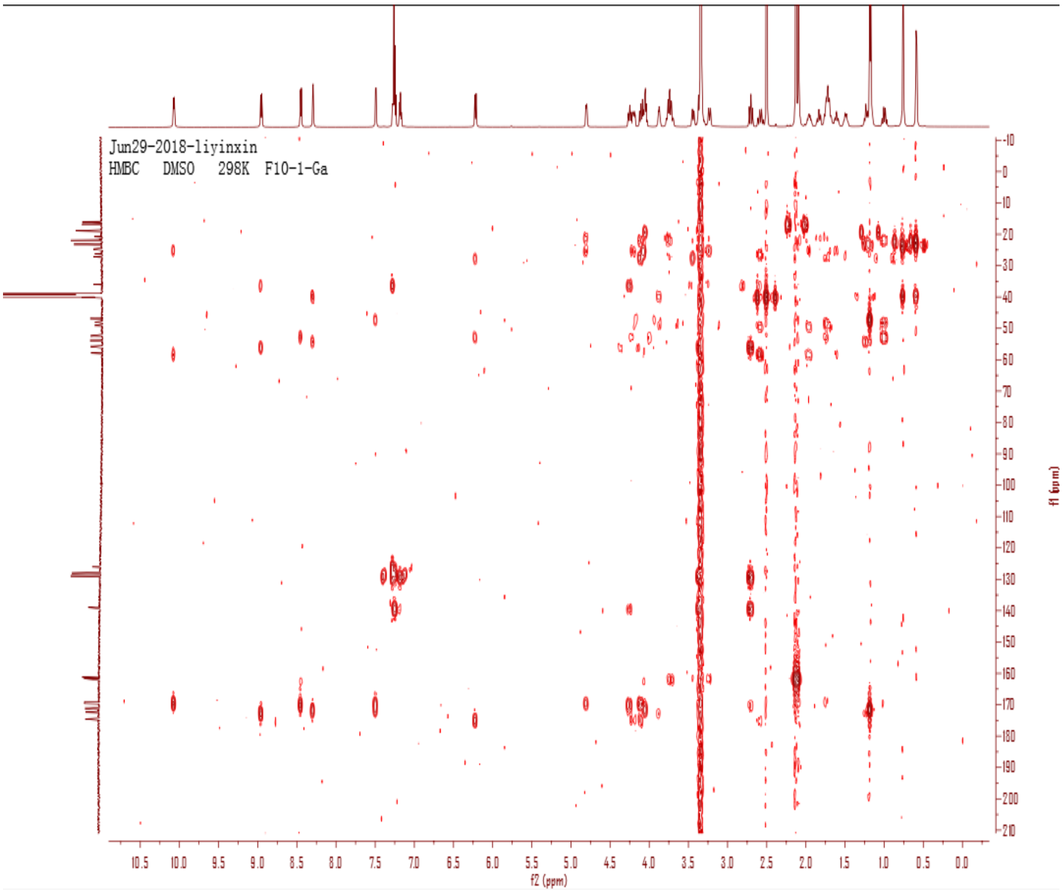

Figure S59. HMBC spectrum of Ga (III)-acremonpeptide E (**7**) in DMSO-d_6.

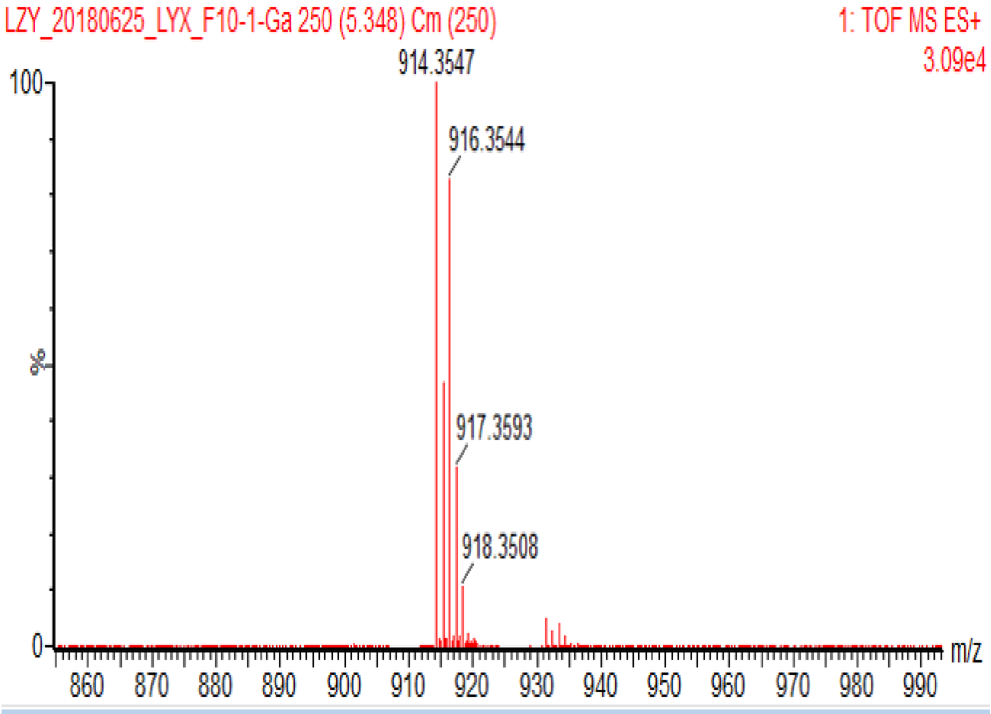

Figure S60. HRESIMS data of Ga (III)-acremonpeptide E (**7**).

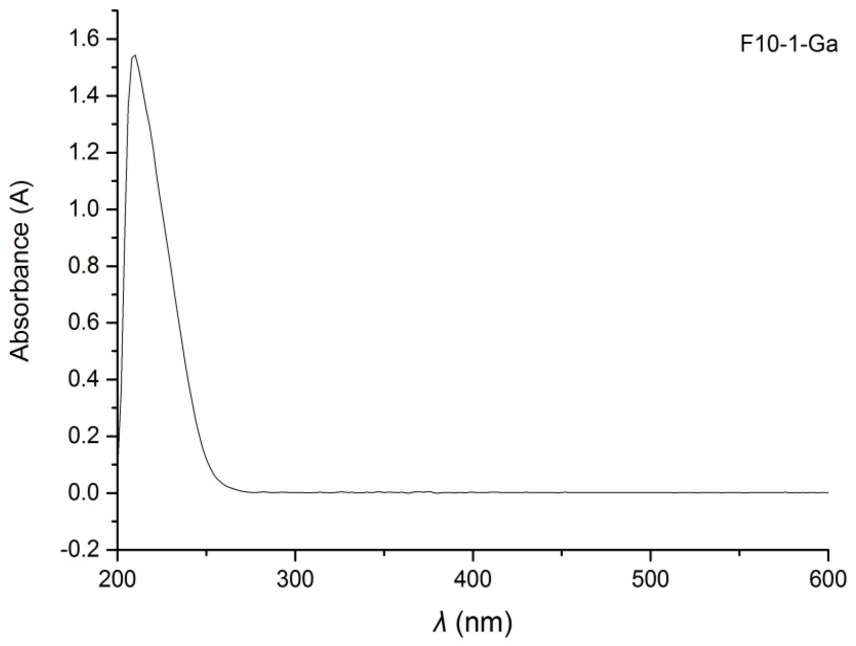

Figure S61. UV spectrum of Ga (III)-acremonpeptide E (**7**) in MeOH.

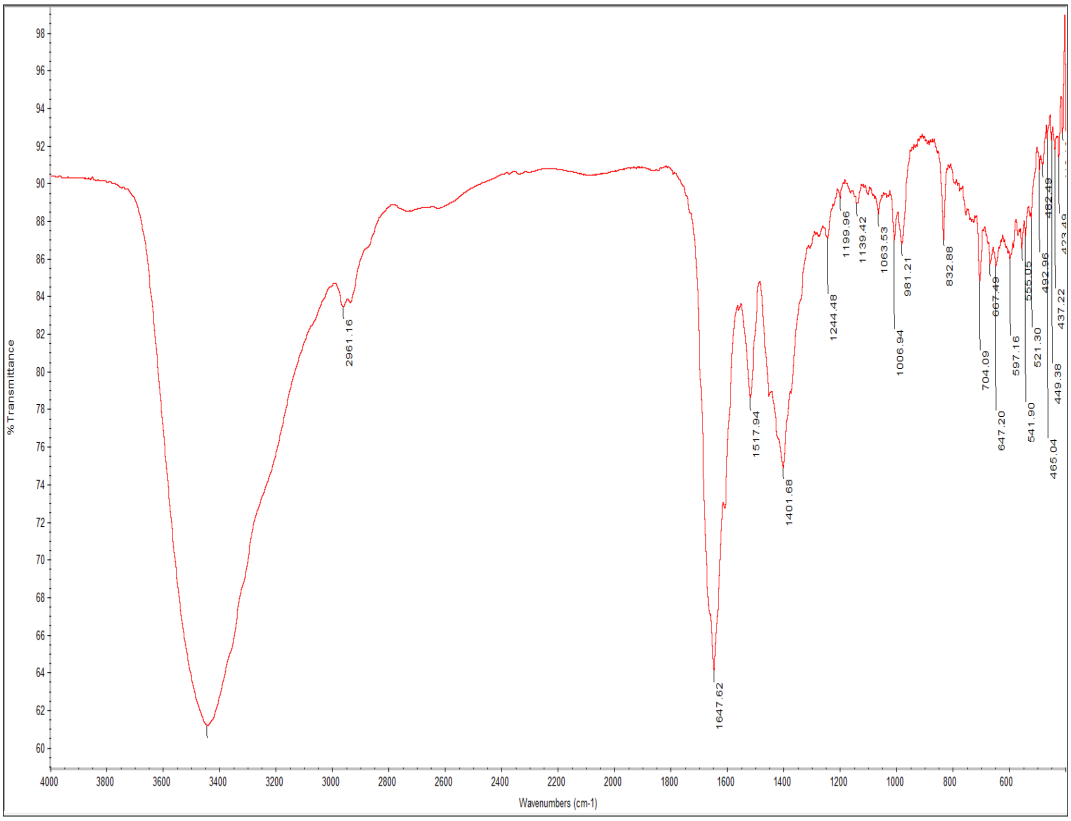

Figure S62. IR spectrum of Ga (III)-acremonpeptide E (**7**).

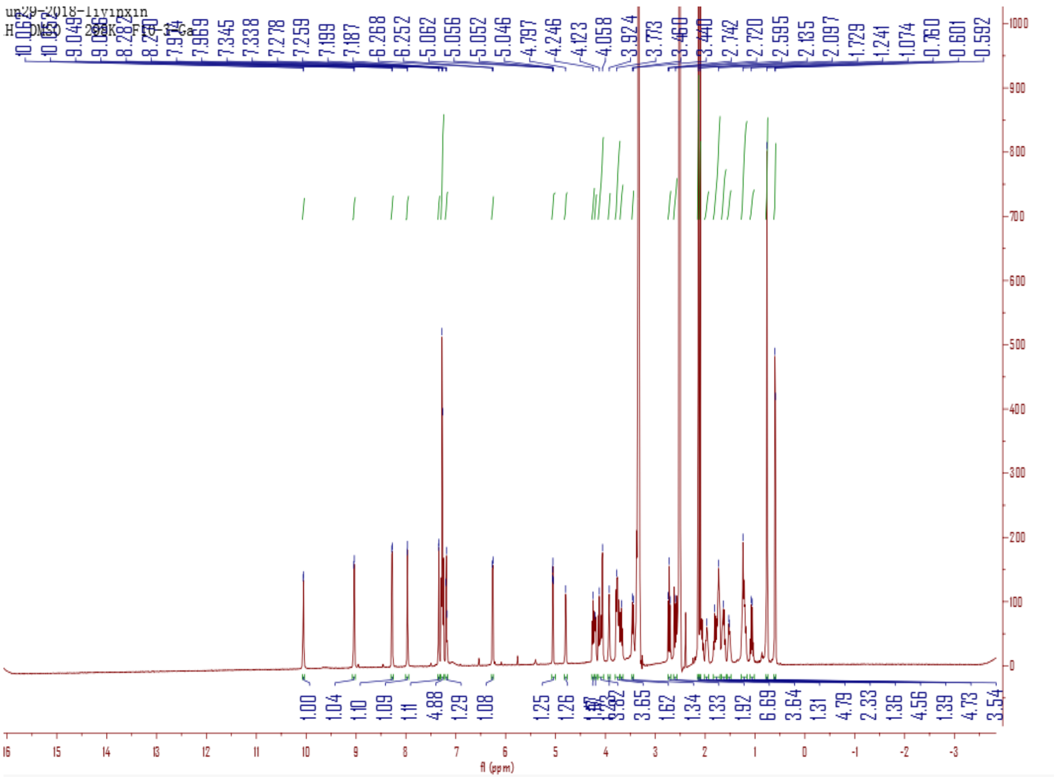

Figure S63. ^{1}H spectrum of Ga (III)-acremonpeptide F (**8**) in DMSO-d_6 (600 MHz).

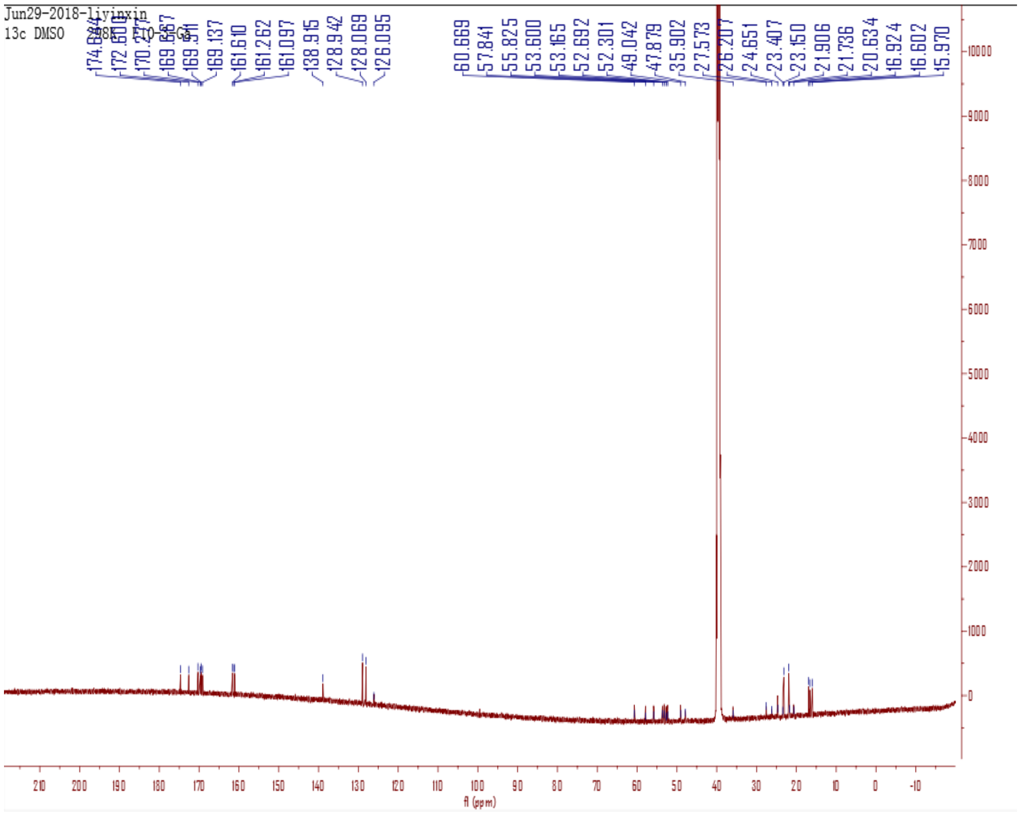

Figure S64. ^{13}C spectrum of Ga (III)-acremonpeptide F (**8**) in DMSO-d_6 (150 MHz).

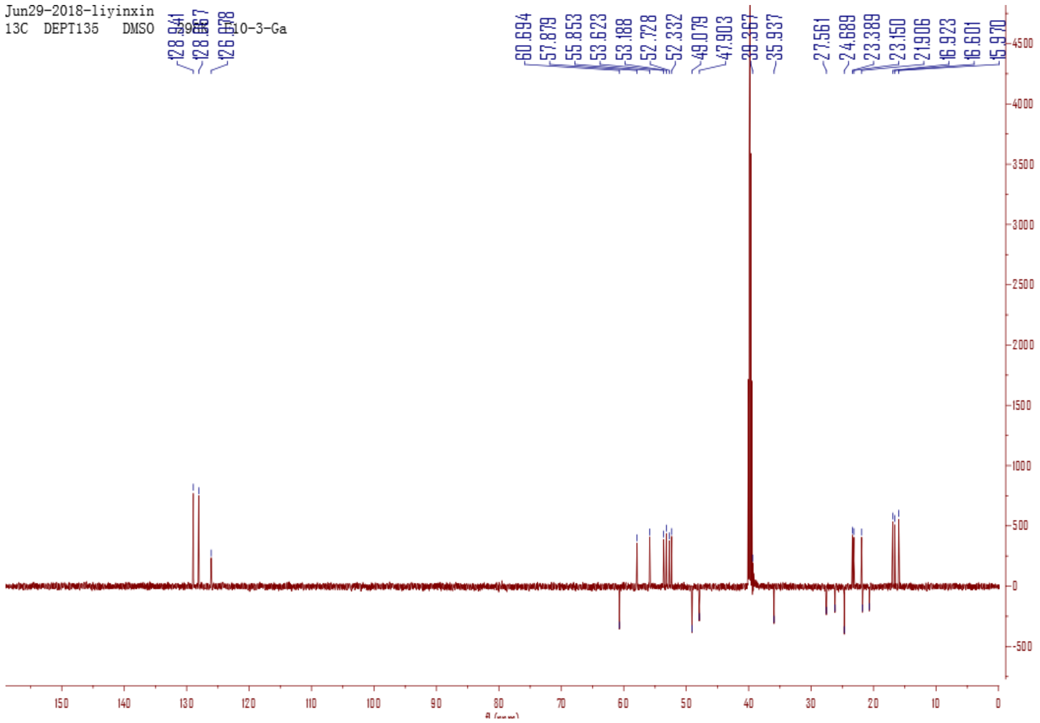

Figure S65. DEPT135 spectrum of Ga (III)-acremonpeptide F (**8**) in DMSO-d_6 (150 MHz).

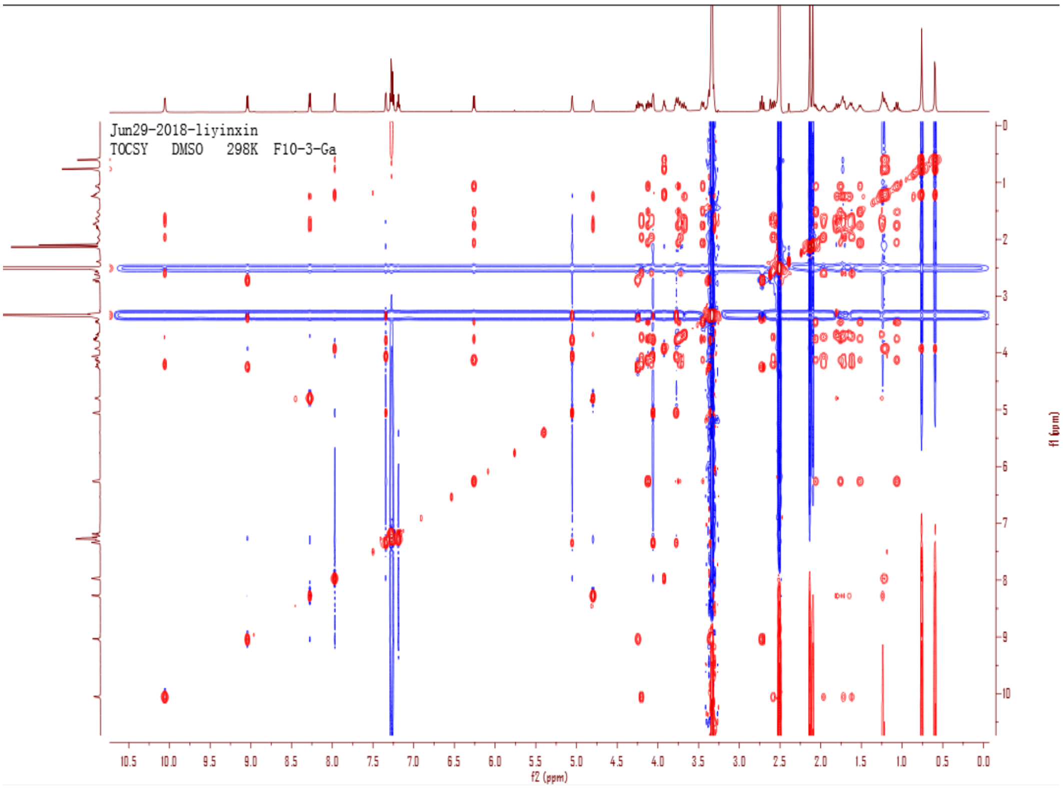

Figure S66. TOCSY spectrum of Ga (III)-acremonpeptide F (**8**) in DMSO-d_6.

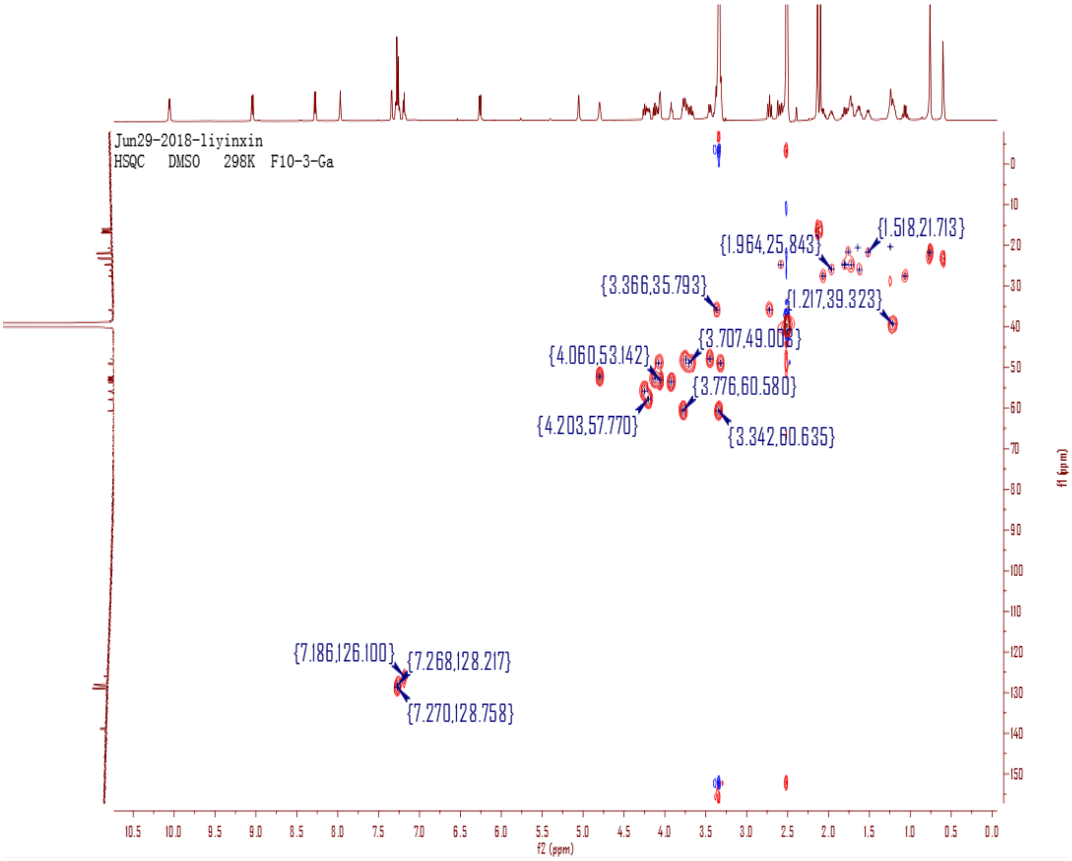

Figure S67. HSQC spectrum of Ga (III)-acremonpeptide F (**8**) in DMSO-d_6.

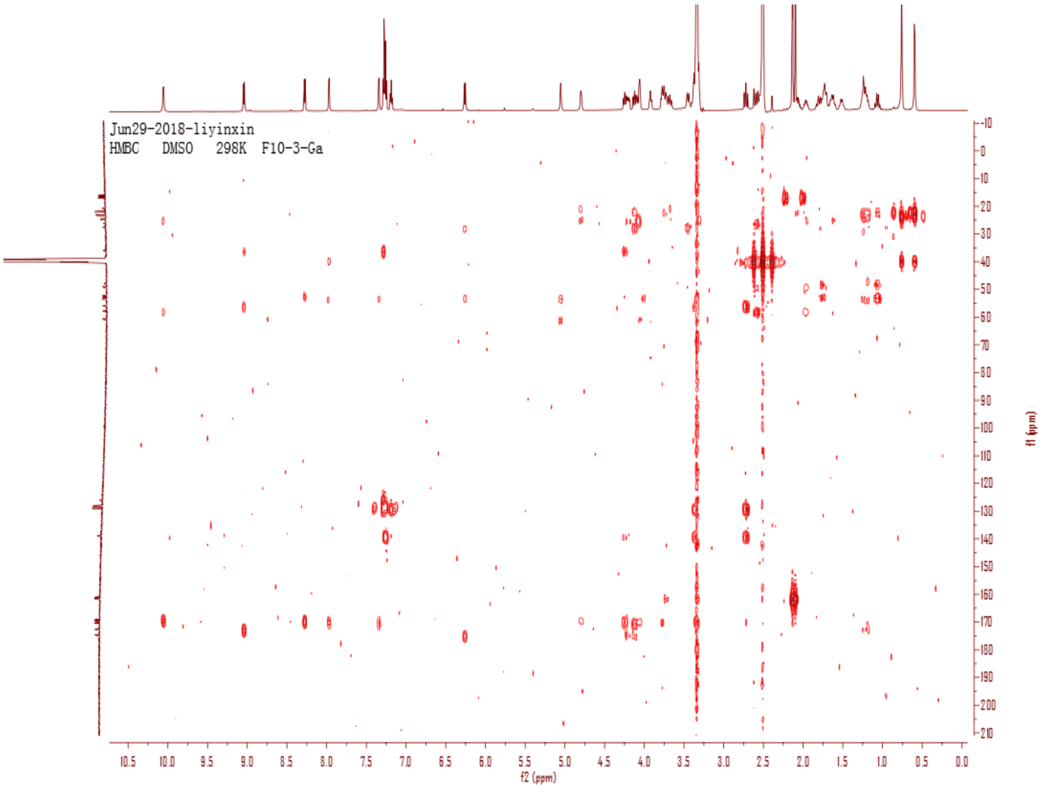

Figure S68. HMBC spectrum of Ga (III)-acremonpeptide F (**8**) in DMSO-d_6.

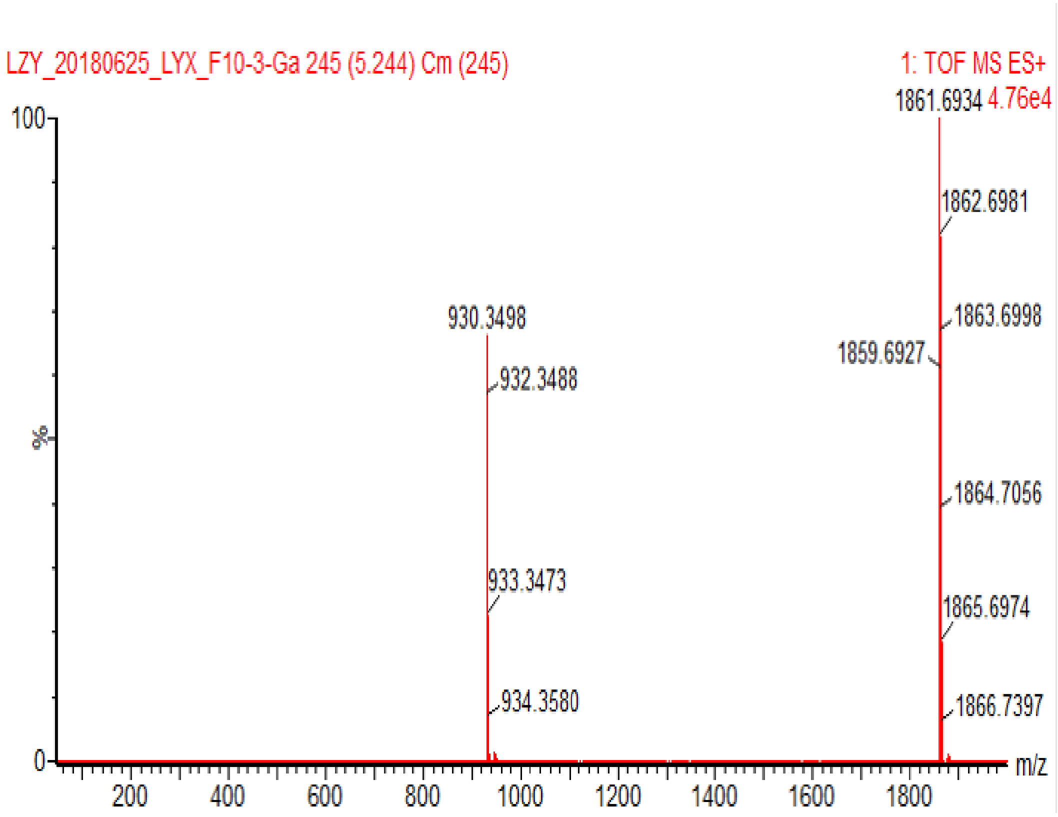

Figure S69. HRESIMS data of Ga (III)-acremonpeptide F (**8**).

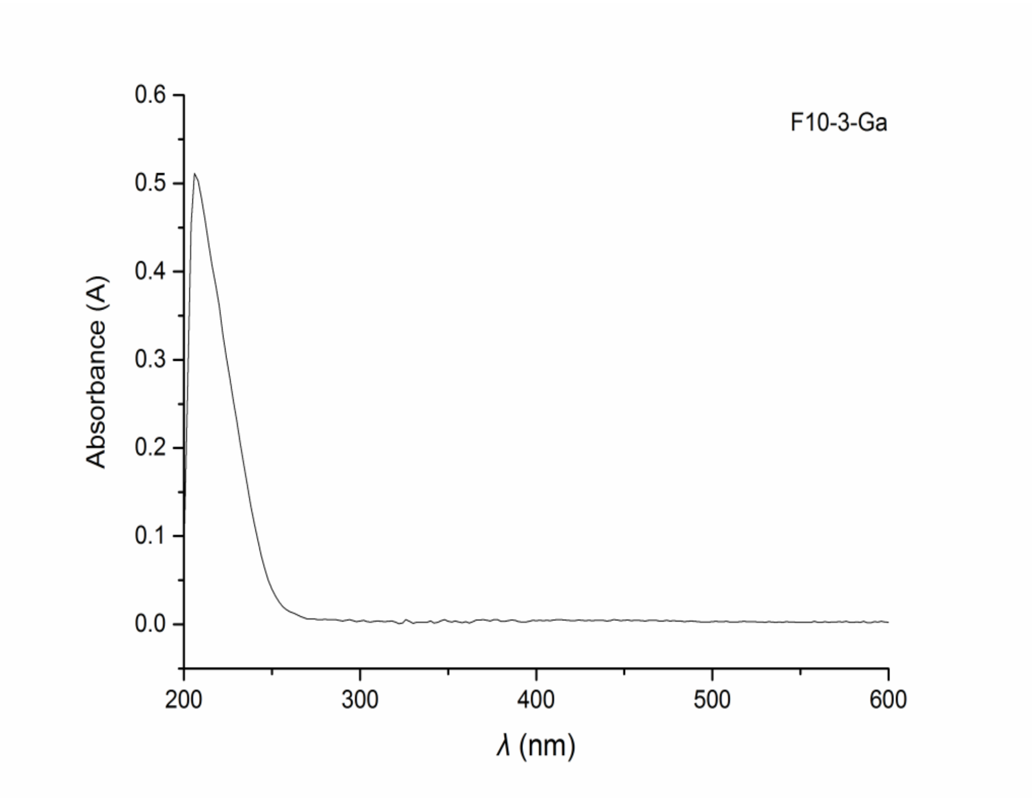

Figure S70. UV spectrum of Ga (III)-acremonpeptide F (**8**) in MeOH.

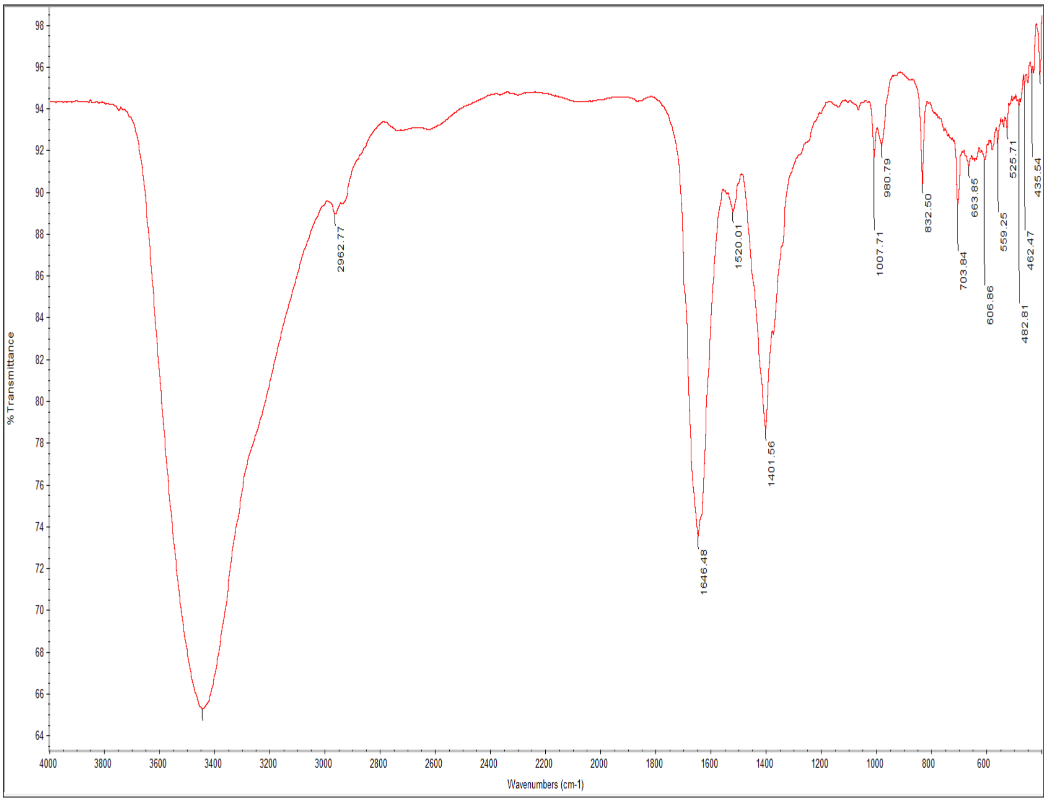

Figure S71. IR spectrum of Ga (III)-acremonpeptide F (**8**).

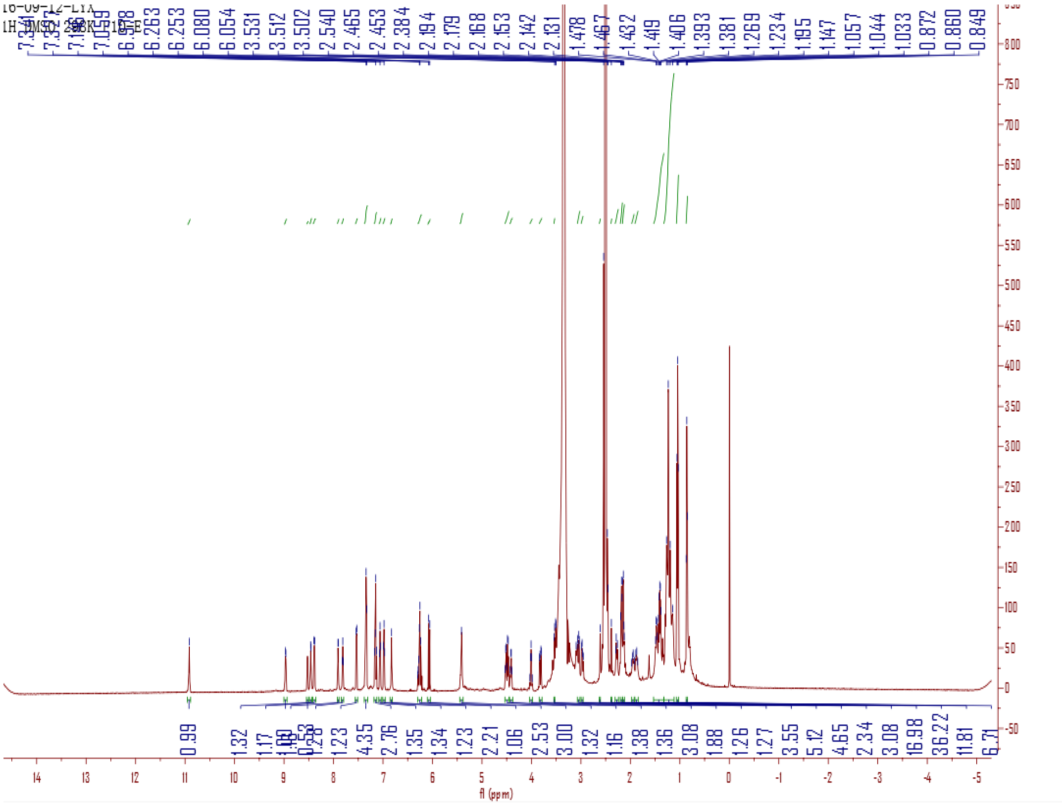

Figure S72. ^{1}H spectrum of aselacin D (**9**) in DMSO-d_6 (600 MHz).

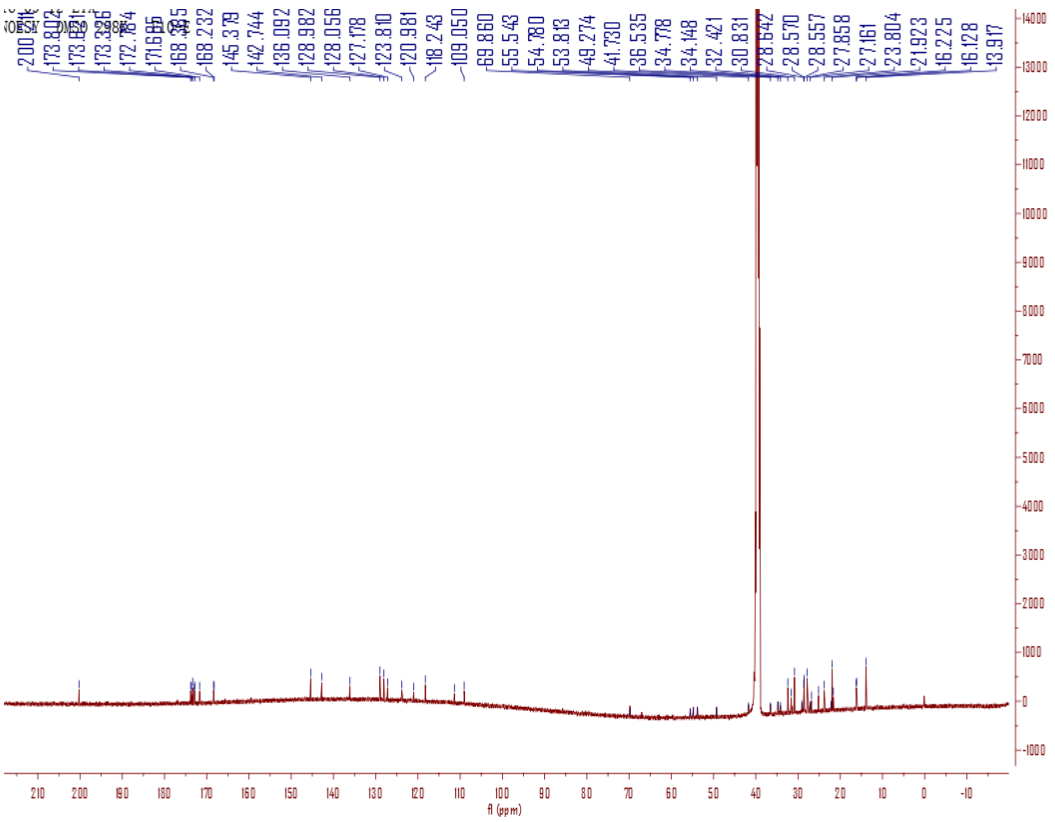

Figure S73. ^{13}C spectrum of aselacin D (**9**) in DMSO-d_6 (150 MHz).

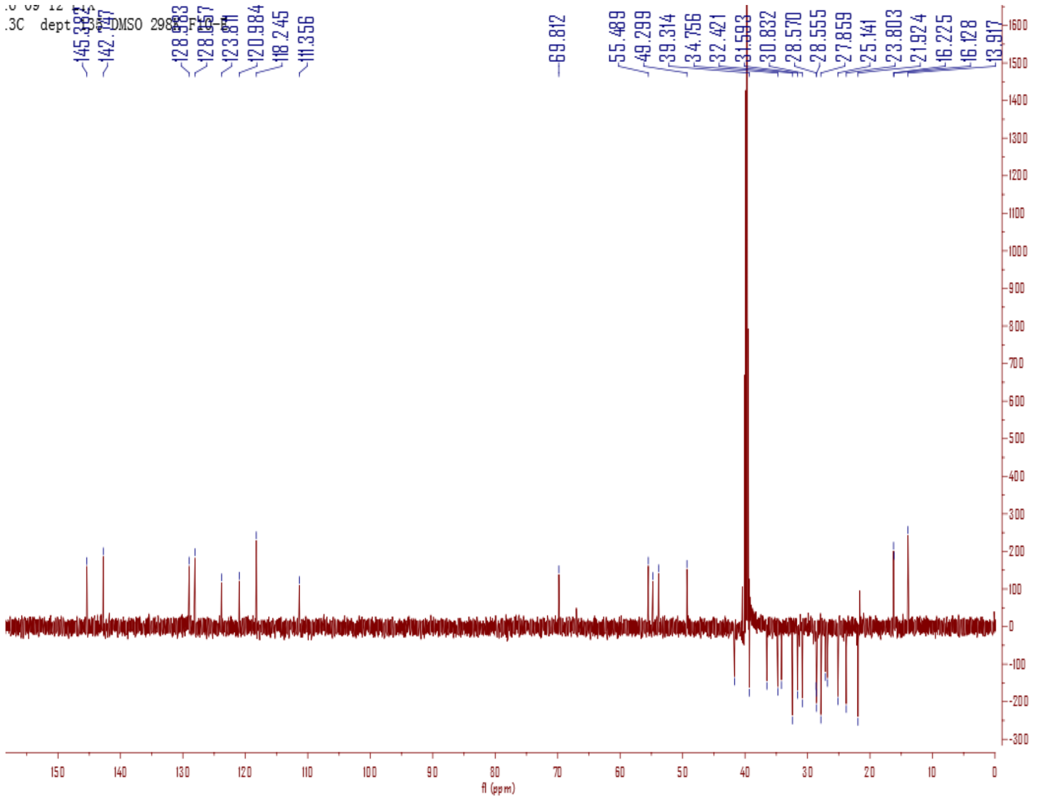

Figure S74. DEPT135 spectrum of aselacin D (**9**) in DMSO-d_6 (150 MHz).

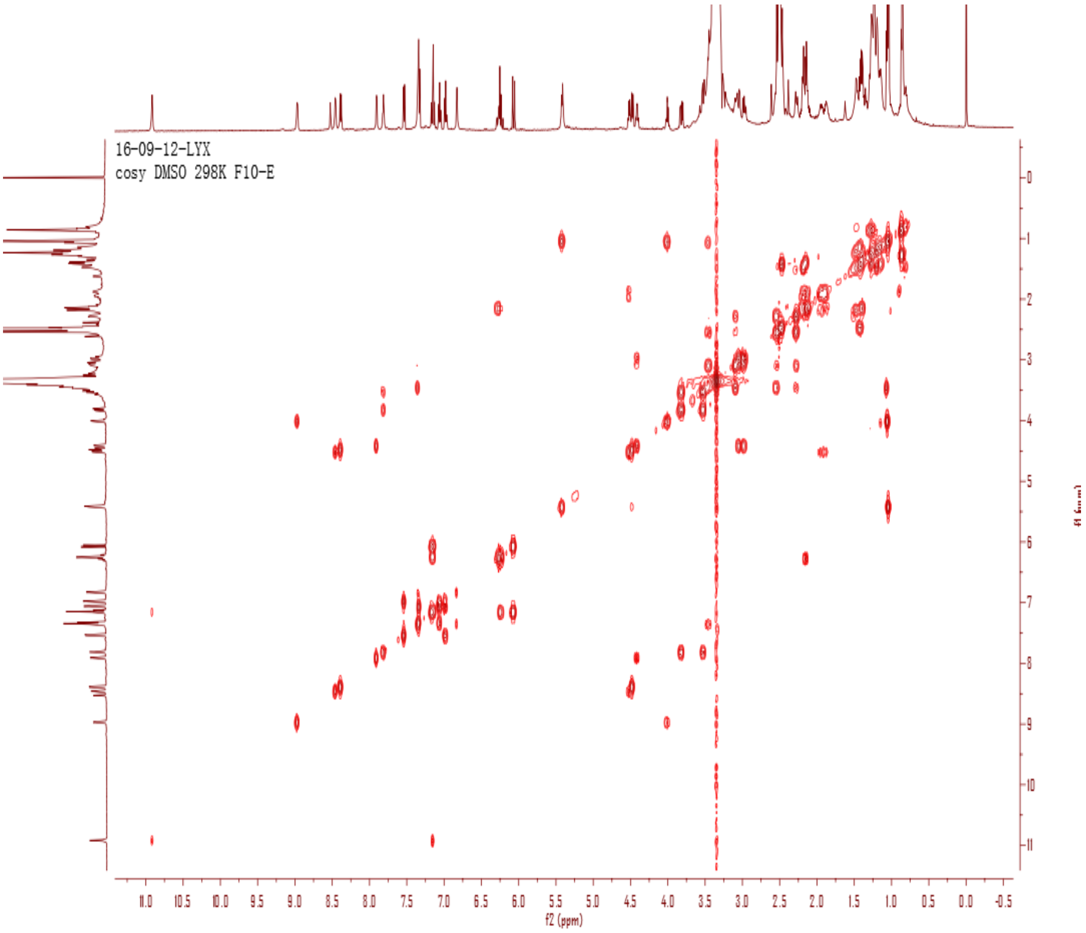

Figure S75. TOCSY spectrum of aselacin D (**9**) in DMSO-d_6

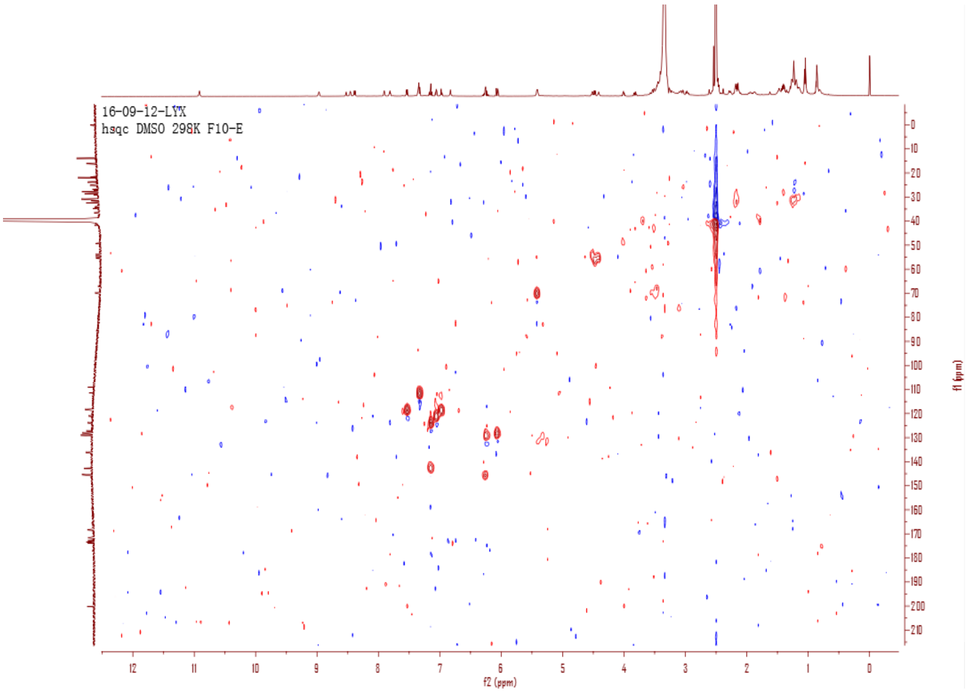

Figure S76. HSQC spectrum of aselacin D (**9**) in DMSO-d_6

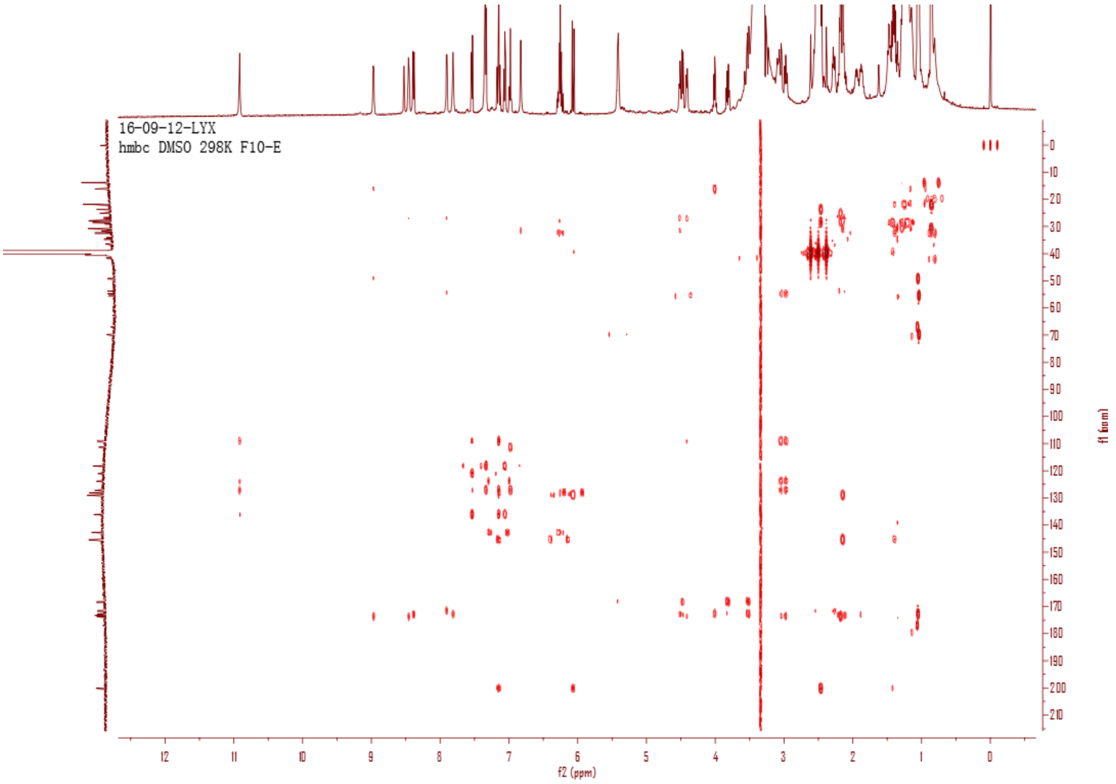

Figure S77. HMBC spectrum of aselacin D (**9**) in DMSO-d_6

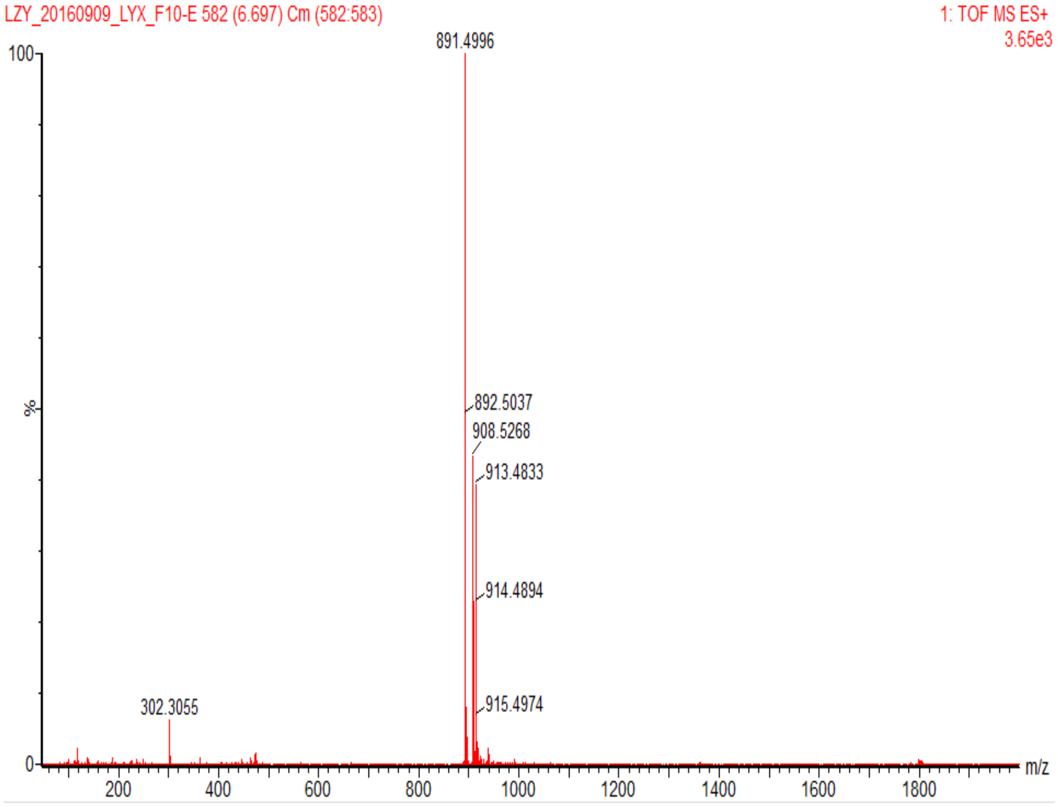

Figure S78. HRESIMS data of aselacin D (**9**)

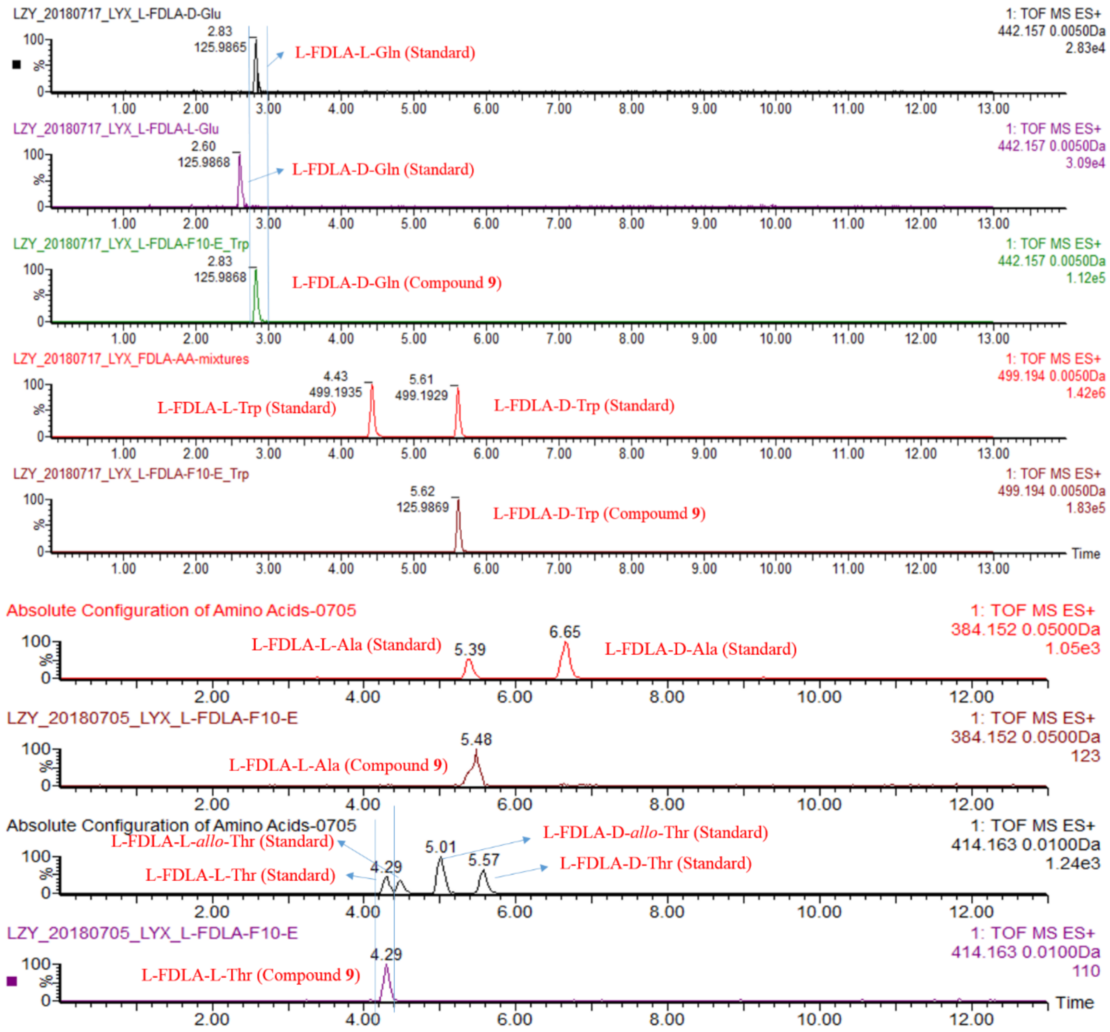

Figure S79. Mass chromatograms of the L-FDLA derivatives of standard amino acids and amino acids from aselacin D (**9**).

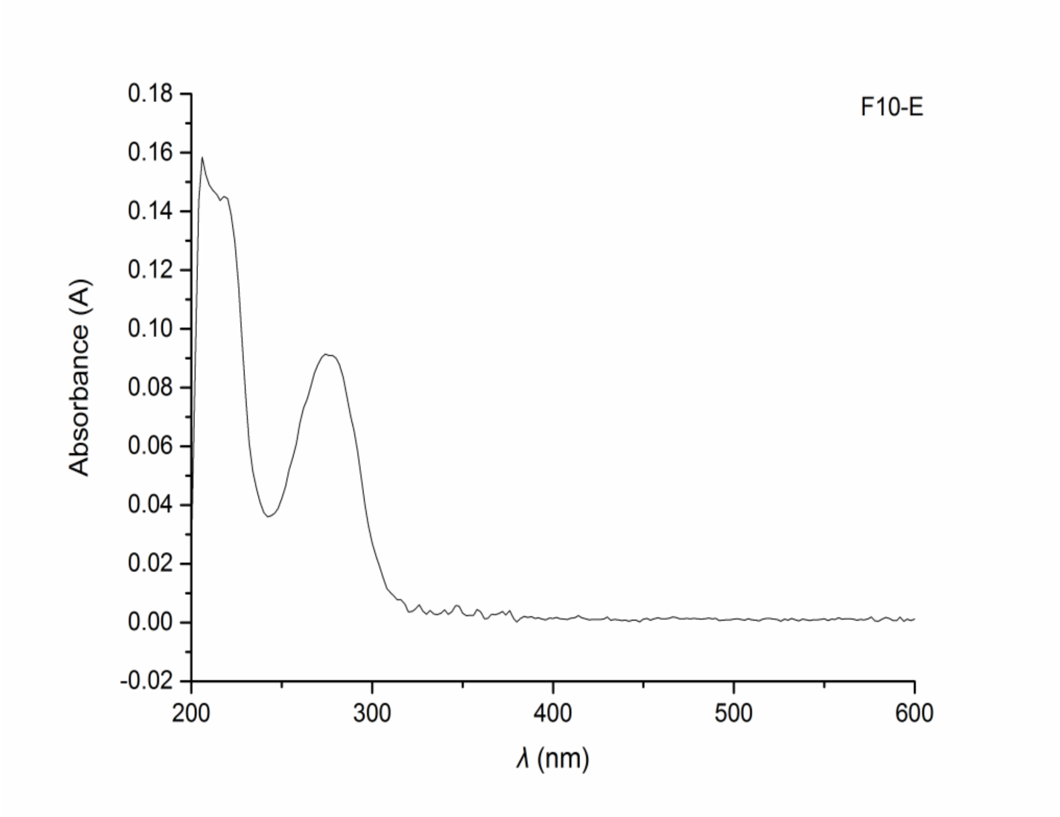

Figure S80. UV spectrum of aselacin D (**9**) in MeOH.

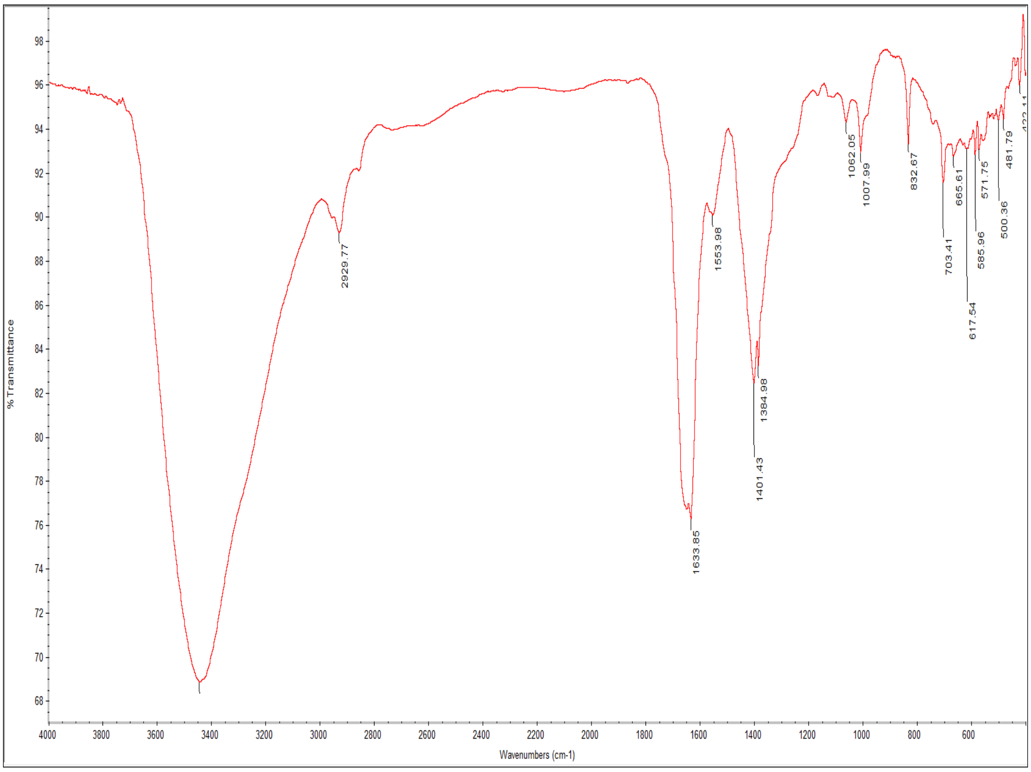

Figure S81. IR spectrum of aselacin D (**9**).
